# Remodeling of the Fission Yeast Cdc42 Cell-Polarity Module via the Sty1 p38 Stress-Activated Protein Kinase Pathway

**DOI:** 10.1016/j.cub.2016.08.048

**Published:** 2016-11-07

**Authors:** Delyan R. Mutavchiev, Marcin Leda, Kenneth E. Sawin

**Affiliations:** 1 Wellcome Trust Centre for Cell Biology, School of Biological Sciences, University of Edinburgh, Michael Swann Building, Max Born Crescent, Edinburgh EH9 3BF, UK; 2 SynthSys (Centre for Synthetic and Systems Biology), School of Biological Sciences, University of Edinburgh, C.H. Waddington Building, Max Born Crescent, Edinburgh EH9 3BF, UK

**Keywords:** cell polarity, Cdc42, MAP kinase, stress, actin, fission yeast, *Schizosaccharomyces pombe*, yeast, Sty1, latrunculin

## Abstract

The Rho family GTPase Cdc42 is a key regulator of eukaryotic cellular organization and cell polarity [1]. In the fission yeast *Schizosaccharomyces pombe*, active Cdc42 and associated effectors and regulators (the “Cdc42 polarity module”) coordinate polarized growth at cell tips by controlling the actin cytoskeleton and exocytosis [2, 3, 4]. Localization of the Cdc42 polarity module to cell tips is thus critical for its function. Here we show that the fission yeast stress-activated protein kinase Sty1, a homolog of mammalian p38 MAP kinase, regulates localization of the Cdc42 polarity module. In wild-type cells, treatment with latrunculin A, a drug that leads to actin depolymerization, induces dispersal of the Cdc42 module from cell tips and cessation of polarized growth [5, 6]. We show that latrunculin A treatment also activates the Sty1 MAP kinase pathway and, strikingly, we find that loss of Sty1 MAP kinase signaling prevents latrunculin A-induced dispersal of the Cdc42 module, allowing polarized growth even in complete absence of the actin cytoskeleton. Regulation of the Cdc42 module by Sty1 is independent of Sty1’s role in stress-induced gene expression. We also describe a system for activation of Sty1 kinase “on demand” in the absence of any external stress, and use this to show that Sty1 activation alone is sufficient to disperse the Cdc42 module from cell tips in otherwise unperturbed cells. During nitrogen-starvation-induced quiescence, inhibition of Sty1 converts non-growing, depolarized cells into growing, polarized cells. Our results place MAP kinase Sty1 as an important physiological regulator of the Cdc42 polarity module.

## Highlights

• LatA depolymerizes actin, disperses cell-polarity proteins, and activates MAPK Sty1
• There is no cell-polarity dispersal after LatA treatment in *sty1* and *wis1* mutants
• Sty1 activation alone is sufficient for cell-polarity dispersal
• Sty1 inactivation repolarizes quiescent cells

## Teaser

Mutavchiev et al. uncover a link between stress signaling and Cdc42-dependent cell polarity in fission yeast. They show that dispersal of the Cdc42 polarity module from growth sites after treatment with the actin drug latrunculin A depends on Sty1, a homolog of mammalian p38 MAP kinase. Activation of Sty1 alone is also sufficient for dispersal.

## Results and Discussion

Fluorescent-protein fusions with CRIB (Cdc42/Rac interactive binding motif)-containing domains of Cdc42 effectors are widely used as reporters of active (GTP-bound) Cdc42 localization in vivo in both budding and fission yeasts [7, 8, 9]. In fission yeast, after treatment with the actin monomer-binding drug latrunculin A (LatA), which leads to acute depolymerization of the actin cytoskeleton, CRIB-3xGFP disperses from cell tips and forms transient ectopic patches on cell sides, as does Cdc42 itself and other components of the Cdc42 polarity module [5, 6, 10]. Because actin filaments can play a role, for example, in vectorial transport of endomembrane-associated Cdc42 or its regulators to cell tips or in endocytic recycling of Cdc42 from the plasma membrane [4, 11, 12, 13], the most straightforward interpretation of this result has been that dispersal of the Cdc42 polarity module from cell tips is a direct consequence of the loss of actin filaments [5, 6].

However, several years ago, in the context of a putative “spindle orientation checkpoint” in fission yeast [14], it was briefly reported that treatment with latrunculin B, a compound related to LatA, leads to activation of the conserved mitogen-activated protein (MAP) kinase Sty1 (also known as Spc1/Phh1 [15, 16, 17]). Sty1 is the fission yeast homolog of budding yeast MAP kinase Hog1 and of mammalian stress-activated protein kinase (SAPK) p38 [18, 19, 20]. Although there is now considerable evidence against the concept of a spindle orientation checkpoint [21, 22, 23], the initial observation of Sty1 activation after latrunculin B treatment was never investigated further. We therefore decided to revisit the question of how the Cdc42 polarity module disperses from cell tips after LatA treatment, and whether Sty1 plays a role in this behavior.

### Latrunculin A Treatment Leads Both to CRIB Dispersal and to Sty1 MAP Kinase Activation

**Figure 1:**
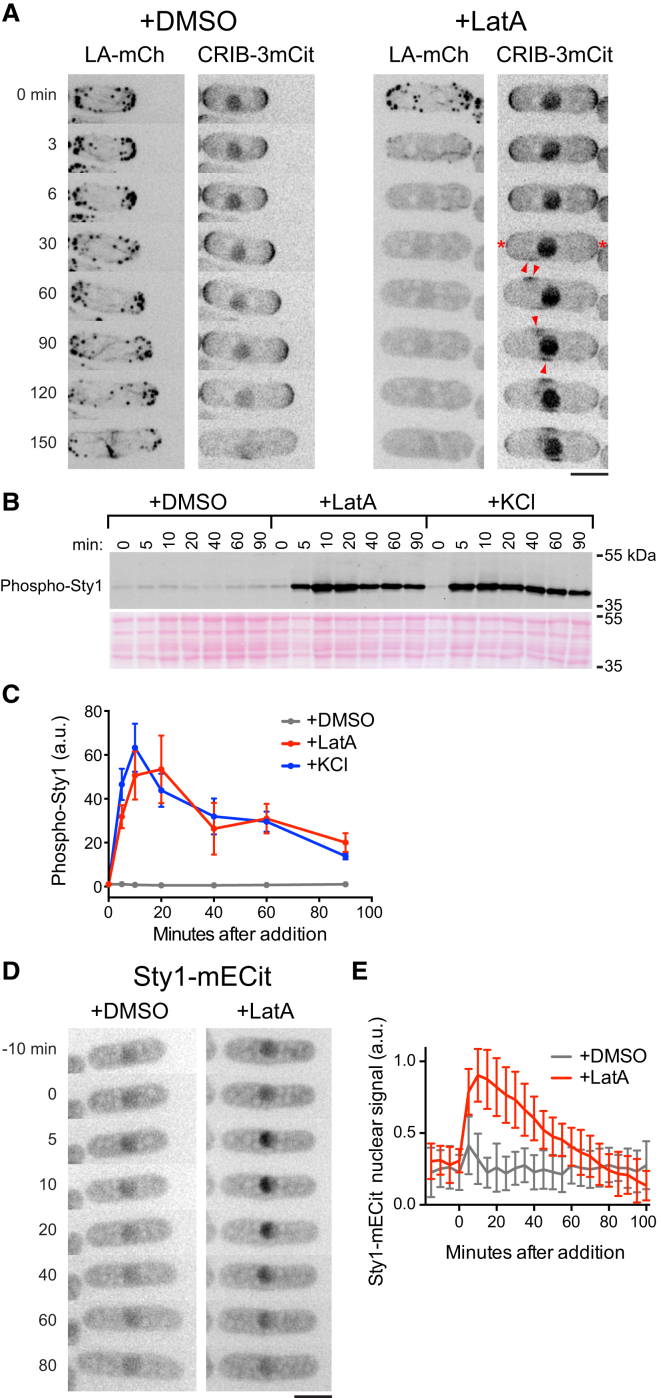
Latrunculin A Treatment Causes Both CRIB Dispersal from Cell Tips and Sty1 MAP Kinase Activation (A) Still images from movies of Lifeact-mCherry (LA-mCh) and CRIB-3xmCitrine (CRIB-3mCit) in wild-type cells after addition of DMSO or 50 μM latrunculin A (LatA). Asterisks indicate dispersal of CRIB from cell tips. Arrowheads indicate examples of ectopic CRIB patches after dispersal. Nuclear CRIB signal is unrelated to Cdc42 (see the Supplemental Experimental Procedures). (B) Anti-phospho-Sty1 western blot of wild-type cell extracts after addition of DMSO, 50 μM LatA, or 0.6 M KCl for the indicated times. Ponceau S stain of the same region of the blot is shown below. (C) Quantification of phospho-Sty1 (a.u.) from experiments of the type shown in (B). Mean values are from three independent experiments. Error bars indicate SEM. (D) Still images from movies of Sty1-mECitrine in wild-type cells after addition of DMSO or LatA. LatA addition leads to net import of Sty1-mECitrine into the nucleus. (E) Quantification of Sty1-mECitrine nuclear fluorescence after addition of DMSO or LatA. Values shown are mean relative intensity (a.u.) of nuclear fluorescence above cytoplasmic fluorescence. Error bars indicate SD. All times shown are relative to addition of DMSO, LatA, or KCl. Scale bars, 5 μm. See also Figure S1 and Movie S1.

We constructed a CRIB-3xmCitrine probe (referred to here simply as CRIB) for long-term time-lapse fluorescence imaging of the Cdc42 polarity module (see the Supplemental Experimental Procedures). Using this together with the F-actin reporter Lifeact-mCherry (here referred to as Lifeact) [24, 25], we confirmed that LatA treatment leads both to actin depolymerization and to CRIB dispersal from cell tips and ectopic patch formation on cell sides (Figures 1A and S1A; Movie S1) [5, 6]. In parallel experiments, the Cdc42 module scaffold protein Scd2 (Scd2-3xmCherry) also dispersed from cell tips after LatA treatment and colocalized with ectopic CRIB patches (Figure S1B) [6, 10]. CRIB dispersal from cell tips was considerably slower than actin depolymerization itself, suggesting that dispersal may not be a direct effect of actin depolymerization. In addition, rather than appearing as spontaneous random patches, ectopic CRIB patches generally moved in a concerted fashion away from cell tips, toward the cell center.

In parallel with these experiments, we demonstrated in two independent assays that LatA treatment leads to activation of Sty1 (Figures 1B–1E). As a component of the fission yeast SAPK pathway, Sty1 is normally activated through phosphorylation of Thr171 and Tyr173 by dual-specificity MAP kinase kinase (MAPKK) Wis1 after a variety of stresses, including hyperosmotic and oxidative stress [26, 27]. Western blotting revealed a strong increase in Sty1 phosphorylation after LatA treatment, comparable in amplitude and duration to that observed after a conventional hyperosmotic salt stress (Figures 1B and 1C). Phosphorylation of Sty1 by Wis1 leads to increased Sty1 in the nucleus, where Sty1 functions to promote stress-activated gene expression (although some Sty1 remains cytoplasmic) [26, 28]. We found that after LatA addition, Sty1-mECitrine accumulated in the nucleus with similar kinetics to those observed for Sty1 phosphorylation (Figures 1D and 1E). We conclude that LatA treatment leads to Sty1 activation at a level comparable to that seen after hyperosmotic salt stress.

### Sty1 Activity Is Necessary for CRIB Dispersal after LatA Treatment

**Figure 2:**
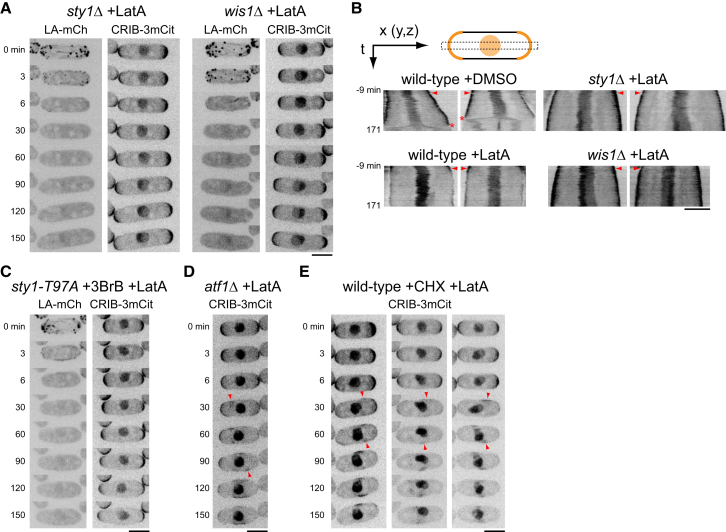
CRIB Dispersal by Latrunculin A Requires the Sty1 MAPK Pathway but Not Sty1-Dependent Gene Expression (A) Still images from movies of Lifeact-mCherry (LA-mCh) and CRIB-3xmCitrine (CRIB-3mCit) in *sty1Δ* and *wis1Δ* cells after addition of 50 μM latrunculin A (LatA). Although LatA depolymerizes the actin cytoskeleton, CRIB does not disperse, and cells continue to elongate. (B) Kymographs from movies of CRIB-3mCit showing rates of cell elongation in the indicated strains after addition of DMSO or LatA. The cartoon summarizes kymograph construction (see also Supplemental Experimental Procedures). The orange curves and circle represent CRIB-3mCit fluorescence at cell tips and in nucleus, respectively. The dashed box represents the region used for kymograph scans along the x-axis. Images used for kymograph analysis are z-projections, and kymograph scans measured average intensity values along a line that is five pixels wide on the y-axis (line width corresponds to height of dashed box); therefore, information from y- and z-dimensions is implicit in kymographs. Arrows indicate orientation of x and time (t) axes in kymographs. Left-hand panels in each pair of kymographs represent cells shown in Figures 1A and 2A and Movie S1. Right-hand panels show additional cells. Arrowheads indicate time of DMSO or LatA addition. Asterisks indicate disappearance of CRIB from cell tips in DMSO-treated cells due to cell division. (C) Still images from movies of LA-mCh and CRIB-3mCit in *sty1-T97A* cells pre-treated with 5 μM 3-BrB-PP1 (3BrB) for 10 min prior to addition of 50 μM LatA in the continued presence of 3BrB. (D) Still images from movies of CRIB-3mCit in *atf1Δ* cells after addition of 50 μM LatA. Arrowheads indicate examples of ectopic CRIB patches. (E) Still images from movies of CRIB-3mCit in wild-type cells pre-treated with 100 μg/mL cycloheximide (CHX) for 10 min prior to addition of 50 μM LatA in the continued presence of CHX. Arrowheads indicate examples of ectopic CRIB patches. CHX treatment causes contortions of nuclei to varying extents, and therefore multiple example cells are shown. All times shown are relative to addition of DMSO or LatA. Scale bars, 5 μm. See also Figures S1 and S2 and Movie S1.

We next asked whether Sty1 plays a role in LatA-induced CRIB dispersal. We imaged CRIB and Lifeact in *sty1Δ* cells and *wis1Δ* cells treated with LatA. Remarkably, in these cells, CRIB remained at cell tips for the duration of imaging (several hours), despite rapid and complete actin depolymerization (Figures 2A and S1A; Movie S1). Scd2 also remained at cell tips (Figure S1B). Moreover, cell elongation continued after actin depolymerization, unlike wild-type cells, in which elongation ceased immediately (Figures 2A and 2B; Movie S1). These results lead to several important conclusions. First, they demonstrate that the SAPK pathway is required for CRIB dispersal after LatA treatment. Second, and in contrast to interpretations of previous experiments [5, 6], they show that the actin cytoskeleton per se is not required for stability of the Cdc42 polarity module at cell tips. Finally, they show that cell elongation can occur in the complete absence of the actin cytoskeleton. Kymograph analysis revealed that cell elongation in LatA-treated *sty1Δ* and *wis1Δ* cells gradually declines over time (Figure 2B). This could be explained as follows: (1) in the initial period after LatA treatment, tip-localized active Cdc42 can drive cell elongation through positive regulation of exocytosis [29]; (2) however, after LatA treatment, membrane proteins involved in exocytosis would no longer be recycled by endocytic retrieval from the plasma membrane, because endocytosis in yeasts depends on the actin cytoskeleton [30]; and therefore (3) such proteins will eventually be depleted from cytoplasmic pools, ultimately leading to cessation of elongation.

Our results suggest a model in which activation of Sty1 by LatA treatment leads to dispersal of the Cdc42 polarity module from cell tips. An alternative view, at least in principle, could be that because Sty1 contributes to multiple cellular pathways [26], *sty1* deletion might lead to a long-term physiological adaptation that fundamentally alters behavior of the Cdc42 module, even prior to any stress (according to this view, LatA-induced activation of Sty1 would be purely coincidental). To rule out this possibility, we imaged CRIB and Lifeact in *sty1-T97A* cells, in which mutation of Thr97 within Sty1’s ATP-binding pocket allows kinase activity to be specifically inhibited by ATP-competitive analogs [31, 32] (*T97A* is the equivalent of an “*as2*” mutation). We treated *sty1-T97A* cells with the analog 3-BrB-PP1 (4-Amino-1-tert-butyl-3-(3-bromobenzyl)pyrazolo[3,4-d]pyrimidine) for less than 10 min, so that no long-term adaptation could occur, and then added LatA in the continued presence of 3-BrB-PP1. In these cells, LatA addition led to actin depolymerization but CRIB remained at cell tips, just as in *sty1Δ* and *wis1Δ* cells, and cells also continued to elongate (Figures 2C and S2A). Collectively, these results demonstrate that LatA-induced CRIB dispersal is not a passive process (e.g., a simple consequence of actin depolymerization) but rather an active process that depends on the SAPK pathway and Sty1 kinase activity. To our knowledge, this is the first indication of such regulation of the Cdc42 polarity module by a MAP kinase pathway.

The best-studied role of Sty1 in response to stress is in the regulation of gene expression, and a key Sty1 substrate is the conserved basic leucine zipper domain (bZIP) transcription factor Atf1 [26, 28, 33, 34, 35]. We found that LatA treatment in *atf1Δ* cells still led to CRIB dispersal (Figure 2D), suggesting that Sty1-dependent changes in gene expression are unlikely to be required for CRIB dispersal. To strengthen these findings, we pre-treated wild-type cells with cycloheximide to inhibit all protein synthesis prior to LatA addition and imaging. In these cells, LatA treatment still led to CRIB dispersal (Figures 2E and S2B). We conclude that the role of Sty1 in promoting CRIB dispersal is independent of stress-induced gene expression.

Polo kinase Plo1, a downstream target of the Sty1 SAPK pathway (phosphorylated on Ser402 after some, but not all, types of stress [36]), has been implicated in regulation of cell polarity [36, 37]. We used 3-BrB-PP1 together with analog-sensitive *plo1-as8* cells [37] as well as *plo1-402A* and *plo1-S402E* mutants [36] to test whether Plo1 is involved in LatA-induced CRIB dispersal. In all cases, LatA treatment led to CRIB dispersal (Figures S1C and S1D), suggesting that Plo1 is not a critical Sty1 target for CRIB dispersal.

### Sty1 Activation Is Sufficient for CRIB Dispersal in the Absence of External Stress

Thus far, our results show that Sty1 is activated by LatA treatment and that Sty1 activity is necessary for LatA-induced CRIB dispersal from cell tips. We next asked whether Sty1 activation alone (without LatA treatment) is sufficient to drive CRIB dispersal.

To test this, we developed a system to rapidly switch on Sty1 activity in vivo in the absence of any external stress. We combined a constitutively active MAPKK allele (*wis1-DD* [38]) with the analog-sensitive *sty1-T97A* allele, together with deletion of two genes encoding tyrosine phosphatases, *pyp1+* and *pyp2+*, which normally attenuate Sty1 MAPK signaling via dephosphorylation of Sty1 Tyr173 [15, 16] (see the Supplemental Experimental Procedures). For simplicity, we will refer to this combination of mutations as *SISA* (stress-independent Sty1 activation). We reasoned that in *SISA* cells grown in the presence of 3-BrB-PP1, Sty1 will be “poised” to be active (because it is phosphorylated by constitutively active Wis1-DD) but nevertheless inhibited by 3-BrB-PP1. Accordingly, upon removal of 3-BrB-PP1, Sty1 activity should rapidly increase.

**Figure 3:**
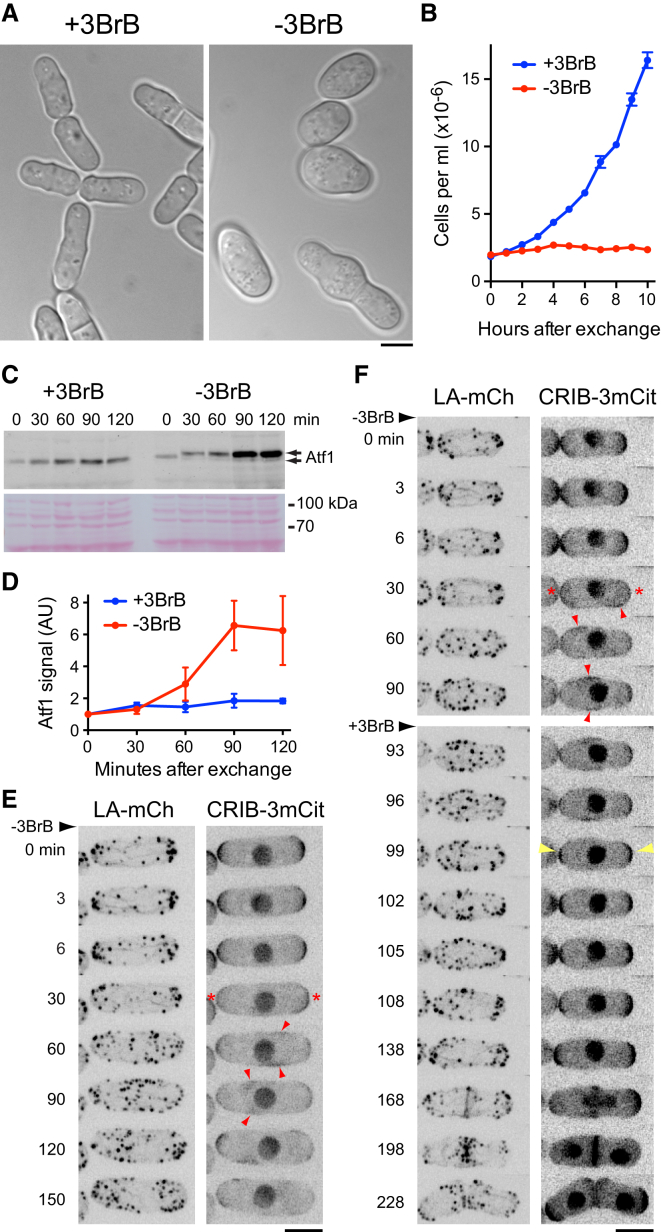
Sty1 Activation Drives CRIB Dispersal in the Absence of Any External Stress (A) DIC images of *SISA* (*wis1-DD sty1-T97A pyp1Δ pyp2Δ*) cells grown in the presence of 3-BrB-PP1 (+3BrB) and 24 hr after 3-BrB-PP1 removal (−3BrB). (B) Cell-number growth curves of *SISA* cells in the presence of 3-BrB-PP1 and after 3-BrB-PP1 removal. Error bars indicate SEM. (C) Anti-Atf1 western blot of extracts from *SISA* cells in the presence of 3-BrB-PP1 (+3BrB) and after removal (−3BrB). Times are relative to initiation of 3-BrB-PP1 removal. After removal, there is a discrete migration shift of Atf1 from 30 min onward, as well as increased levels of Atf1, especially at 90 and 120 min. Ponceau S stain of the same region of the blot is shown below. (D) Quantification of Atf1 levels in *SISA* cells from experiments of the type shown in (C). Mean values are from three independent experiments. Error bars indicate SEM. (E) Still images from movies of Lifeact-mCherry (LA-mCh) and CRIB-3xmCitrine (CRIB-3mCit) in *SISA* cells after 3-BrB-PP1 removal. Asterisks indicate CRIB dispersal from cell tips. Arrowheads indicate ectopic CRIB patches. Times are relative to initiation of removal. (F) Still images from movies of LA-mCh and CRIB-3mCit in *SISA* cells after 3-BrB-PP1 removal and subsequent 3-BrB-PP1 re-addition 90 min later. Asterisks indicate CRIB dispersal from cell tips. Red arrowheads indicate ectopic CRIB patches. Yellow arrowheads indicate recovery of CRIB to cell tips upon 3-BrB-PP1 re-addition. Times are relative to initiation of removal. Scale bars, 5 μm. See also Figure S3 and Movie S2.

*SISA* cells displayed normal growth and morphology when grown in 5 μM 3-BrB-PP1 but stopped dividing upon removal of 3-BrB-PP1, eventually becoming large and swollen in the middle (Figures 3A and 3B). *SISA* cells were also unable to form colonies on solid media lacking 3-BrB-PP1. To confirm that these phenotypes were associated with increased Sty1 activity, we assayed Atf1 in *SISA* cells by western blotting. Removal of 3-BrB-PP1 led to a small but reproducible shift in Atf1 migration on SDS-PAGE, as well as significantly increased Atf1 levels, matching what occurs after stress-induced Sty1 activation (Figures 3C and 3D) [34, 39, 40].

We next imaged CRIB and Lifeact in *SISA* cells (Figures 3E and 3F; Movie S2). In the presence of 3-BrB-PP1, CRIB and Lifeact distributions were indistinguishable from wild-type cells. However, upon removal of 3-BrB-PP1, CRIB dispersed from cell tips within ∼30 min, and ectopic CRIB patches appeared on cell sides, as in LatA-treated wild-type cells. Other components of the Cdc42 polarity module also dispersed from cell tips and formed ectopic patches (Figures S3A and S3B), and concomitantly the actin cytoskeleton became depolarized (but not depolymerized; Figures 3E and S3C). These results indicate that an acute increase in Sty1 activity is sufficient to promote dispersal of the Cdc42 polarity module from cell tips, even in the absence of any external stress. In conjunction with our previous observations, this provides extremely strong evidence that the Cdc42 polarity module is specifically regulated by the Sty1 MAP kinase pathway.

We also used *SISA* cells to investigate how Sty1 inactivation affects CRIB localization in cells containing already-dispersed CRIB. Sty1 was activated by 3-BrB-PP1 removal and then inactivated 90 min later by re-addition of 3-BrB-PP1 (Figure 3F; Movie S3). Strikingly, upon re-addition of 3-BrB-PP1, the dispersed CRIB rapidly returned to cell tips, and this preceded the return of actin to cell tips. This suggests that key polarity landmarks are retained at cell tips after Sty1-activated CRIB dispersal and that, upon Sty1 inactivation, the Cdc42 polarity module can follow these cues independent of the polarization of the actin cytoskeleton.

### Inhibition of Sty1 during Quiescence Leads to Cell Repolarization

**Figure 4:**
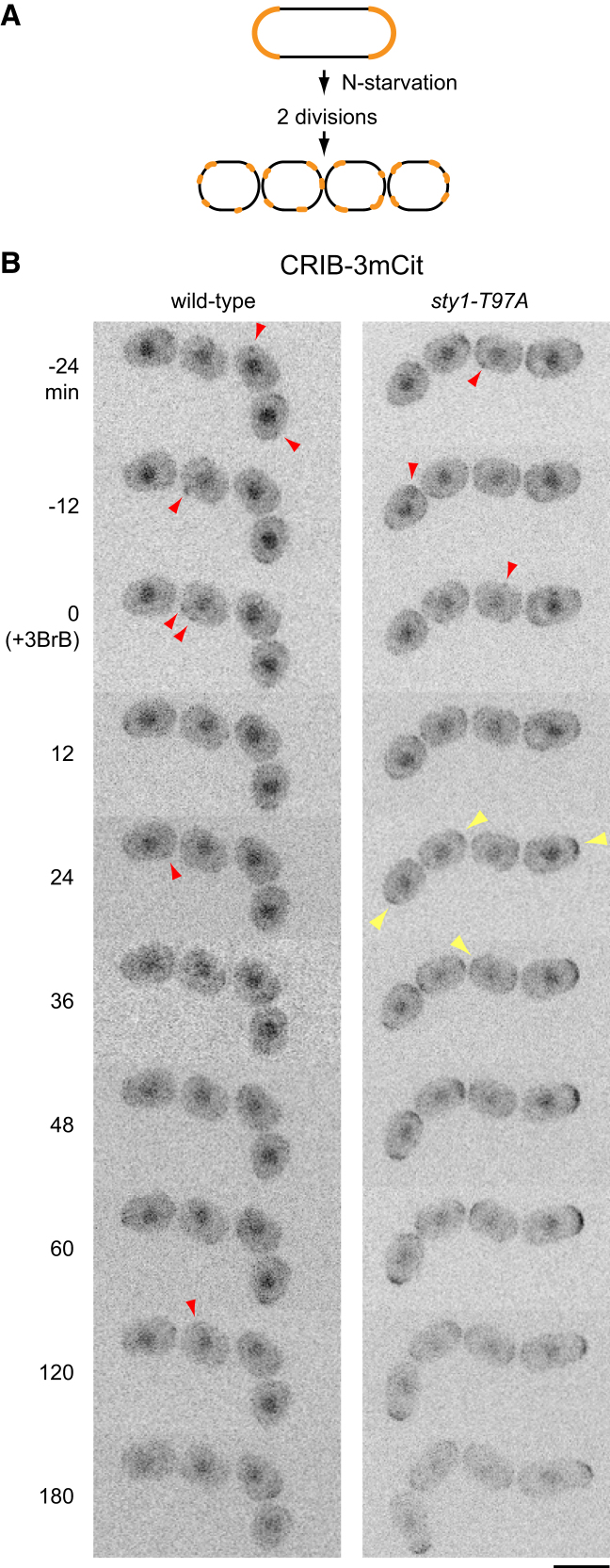
Inhibition of Sty1 Leads to Repolarization of Quiescent Cells (A) Cartoon of cell divisions after nitrogen starvation (N starvation) in heterothallic cells. Upon N starvation, cells divide twice en route to quiescence, with relatively little elongation during interphase. This produces a chain of four small granddaughter cells that lack any overall polarization. Cell-polarity machinery (e.g., CRIB-3xmCitrine) is shown in orange. (B) Still images from movies of CRIB-3xmCitrine (CRIB-3mCit) in N-starved wild-type and *sty1-T97A* cells before and after addition of 3-BrB-PP1 (3BrB). Chains of four granddaughter cells are shown. Red arrowheads indicate ectopic patches of CRIB-3mCit. Yellow arrowheads indicate repolarization of CRIB-3mCit. Note the elongation of *sty1-T97A* cells after 3BrB-induced repolarization, and the absence of repolarization/elongation in wild-type cells. Times shown are relative to 3BrB addition; at 0 min, cells have already been N starved for 11 hr, including 1 hr of imaging. Scale bar, 5 μm. See also Movie S4, in which ectopic CRIB patches are more apparent.

The experiments described thus far involve activation of Sty1 either via external stress (LatA) or by use of *SISA* cells. We therefore investigated a role for Sty1 in cell polarity in an additional physiological setting, nitrogen starvation (N starvation), which is a prerequisite to mating and meiosis in fission yeast [41, 42]. Upon N starvation, cells normally divide twice in succession without significant interphase cell elongation, leading to the generation of four small cells (Figure 4A). Homothallic (self-mating) cells then mate, whereas heterothallic cells in the absence of a mating partner enter a long-term, quiescent G0-like state [43] in which the Cdc42 polarity module appears to become depolarized [44]. Because *sty1Δ* mutants are partially sterile [17, 34], we hypothesized that Sty1 may be involved in maintaining this depolarized state, which might be important for an exploratory phase of cell polarity when mating partners are present [44].

We imaged CRIB in N-starved heterothallic wild-type and *sty1-T97A* cells before and after addition of 3-BrB-PP1 (Figure 4B; Movie S4). In the absence of 3-BrB-PP1, all cells showed disperse, dynamic patches of CRIB, and these patches remained dynamic, without any cell elongation, for the duration of imaging (4 hr; Movie S4). This dynamic behavior was unchanged by addition of 3-BrB-PP1 to wild-type cells. However, after 3-BrB-PP1 addition to *sty1-T97A* cells, CRIB rapidly localized to cell tips, and cells resumed elongation, even though this is futile, as *sty1Δ* cells rapidly lose viability upon N starvation [15, 16, 17].

The recovery of CRIB to cell tips and resumption of cell elongation upon Sty1 inhibition demonstrate that Sty1 activity is critical for maintaining a non-polarized Cdc42 module in N-starved quiescent cells. Although many mutants in the SAPK pathway have defects in mating and meiosis, this result may help to explain why *sty1Δ* and *wis1Δ* mutants in particular continue to elongate upon N starvation, unlike other mutants in the pathway [45, 46].

### The Fission Yeast SAPK Pathway in Regulation of Cell Polarity

The fission yeast SAPK pathway is activated by a wide variety of stresses, including hyperosmotic stress, oxidative stress, temperature stress, nutritional stress, heavy metals, and hypergravity [26, 27]. Our demonstration that Sty1 activation is sufficient for CRIB dispersal independent of any external stress is particularly important because each type of SAPK-activating stress is likely to have additional type-specific effects on cell physiology. With the exception of oxidative stress, in essentially all cases the route from initial stress to Sty1 activation is poorly understood [27]. Currently, it is not clear exactly how LatA treatment leads to Sty1 activation. One interesting possibility is that LatA treatment provokes a specific “actin stress response” that signals to the SAPK pathway; this certainly merits further investigation, as it would suggest the possibility of a checkpoint that disrupts cell polarity in response to cytoskeleton depolymerization. Alternatively, the pathway from LatA treatment to Sty1 activation could involve additional, non-actin targets of LatA, independent of actin depolymerization, although this would not affect our conclusions concerning Sty1 regulation of the Cdc42 polarity module. Future work will help to illuminate how LatA activates the SAPK pathway.

The system of SAPK-dependent cell-polarity regulation in fission yeast described here does not appear to have a direct counterpart in budding yeast. Hog1, the budding yeast homolog of Sty1 and mammalian p38, is strongly activated by osmotic stress but only weakly/moderately by several other stresses (and with significantly different kinetics [19]). Consistent with this, LatA treatment does not activate Hog1 [47]. In addition, in spite of extensive genetic and biochemical analysis over many years, there is currently no evidence that the Hog1 pathway regulates the Cdc42 polarity module, mating efficiency, or responses to nutrient deprivation [18, 19]. Treatment of budding yeast with the related compound latrunculin B has been shown to activate a different MAP kinase, Slt2 (also known as Mpk1 [48]), but it is not clear what relation, if any, this system bears to our work. Both the kinetics and other qualitative aspects of Slt2 activation after latrunculin B treatment [48] are markedly different from what we observe for Sty1 after LatA treatment. Moreover, Slt2 is part of the cell-integrity MAPK pathway—a different pathway altogether from the SAPK pathway—and rather than regulating cell polarity, Slt2 promotes G2 cell-cycle arrest. We speculate that in spite of a largely conserved “parts list” for MAPK signaling and cell-polarity regulation in budding and fission yeast, the different modes of polarized growth specific to each yeast [3] have led to different wiring patterns for MAP kinase activation and downstream targets. Further understanding of SAPK-dependent control of cell polarity in fission yeast may help to determine whether similar control exists in metazoan cells. An additional consequence of our results is that, for some cell types, it may be necessary to re-evaluate experiments that use LatA to investigate how the actin cytoskeleton contributes to various cellular functions, because observed effects may be the result of a stress response rather than actin depolymerization per se.

Although the molecular mechanisms by which Sty1 regulates the Cdc42 polarity module remain to be elucidated, two observations in particular provide some insight. First, although Sty1 activation leads to dispersal of CRIB and other components of the Cdc42 module from cell tips, these nevertheless remain largely concentrated in patches on the plasma membrane [5]. This indicates that the Cdc42 module is at least partially intact under these conditions, albeit misplaced and more dynamic. Second, in our experiments in which 3-BrB-PP1 is first removed from *SISA* cells and then later added back, the recovery of CRIB to cell tips is very rapid, occurring within a few minutes. Collectively, these results suggest that Sty1 may act to negatively regulate the coupling of the Cdc42 module to cell-polarity landmarks [3], some of which may remain at cell tips under stress conditions. Further confirmation of this view will rely on the identification of the proteins involved in coupling, together with an understanding of how they are directly or indirectly regulated by Sty1.

## Experimental Procedures

Standard fission yeast genetics and imaging techniques were used throughout. Detailed descriptions of strain construction, physiological experiments, and microscopy methods are provided in the Supplemental Experimental Procedures.

## Author Contributions

D.R.M. and K.E.S. designed the experiments, D.R.M. performed the experiments; M.L. created analytical tools and performed the analysis; and D.R.M. and K.E.S. wrote the paper.

## Supplemental Information

Document S1. Supplemental Experimental Procedures and Figures S1–S3

Movie S1. CRIB Dispersal after Latrunculin A-Induced Actin Depolymerization Requires the Sty1 MAP Kinase Pathway Lifeact-mCherry and CRIB-3xmCitrine in wild-type cells after addition of DMSO, and in wild-type, *sty1*Δ, and *wis1*Δ cells after addition of 50 μM latrunculin A (LatA). Cells correspond to those shown in Figures 1A and 2A. In wild-type cells, actin depolymerization by LatA leads to CRIB dispersal from cell tips and formation of ectopic CRIB patches on cell sides. In *sty1*Δ and *wis1*Δ cells, CRIB remains at cell tips in spite of actin depolymerization, and *sty1*Δ and *wis1*Δ cells continue to elongate. Images are at 3-min intervals; total elapsed time is 180 min; time compression is 4,500×.

Movie S2. Stress-Independent Activation of Sty1 Leads to CRIB Dispersal and Cell Depolarization Lifeact-mCherry and CRIB-3xmCitrine in *SISA* cells after 3-BrB-PP1 removal, which leads to Sty1 activation in the absence of external stress. The cell corresponds to that shown in Figure 3E. The timing of 3-BrB-PP1 removal is as indicated; the first few frames shown are prior to removal. Upon 3-BrB-PP1 removal, CRIB disperses from cell tips and forms ectopic patches on cell sides, actin becomes depolarized, but not depolymerized, and cell elongation ceases. Images are at 3-min intervals; total elapsed time is 180 min; time compression is 4,500×.

Movie S3. Rapid Restoration of Cell Polarity by Inhibition of Sty1 in Sty1-Activated *SISA* Cells Lifeact-mCherry and CRIB-3xmCitrine in *SISA* cells after 3-BrB-PP1 removal, followed by 3-BrB-PP1 re-addition 90 min later. The cell corresponds to that shown in Figure 3F. The timing of 3-BrB-PP1 removal and re-addition is as indicated; the first few frames shown are prior to removal. Just before 3-BrBPP1 re-addition, the movie pauses to show the state of CRIB and actin prior to re-addition. As in Movie S2, 3-BrB-PP1 removal leads to dispersal of CRIB from cell tips, ectopic CRIB patch formation, depolarized actin cytoskeleton, and cessation of cell elongation. 3-BrB-PP1 re-addition quickly reverses all of these effects, with the return of CRIB to cell tips preceding repolarization of the actin cytoskeleton. Images are at 3-min intervals; total elapsed time is 180 min; time compression is 4,500×.

Movie S4. Inhibition of Sty1 Causes Repolarization of Depolarized, Quiescent Cells CRIB-3xmCitrine in nitrogen-starved wild-type and *sty1-T97A* cells in the absence of 3-BrB-PP1 and after addition of 3-BrB-PP1. Chains of four granddaughter cells are shown. The timing of the addition of 3-BrB-PP1 (or mock treatment) is as indicated. At this time, cells have already been nitrogen starved for 11 hr. 3-BrB-PP1 addition leads to repolarization of CRIB to cell tips and cell elongation in *sty1-T97A* cells, but not in wild-type cells. Mock treatment maintains the depolarized state in both. Images are at 6-min intervals; total elapsed time is 240 min; time compression is 9,000×.

Document S2. Article plus Supplemental Information

## References

[1] Etienne-Manneville S. (2004). Cdc42—the centre of polarity. J. Cell Sci..

[2] Rincón S.A., Estravís M., Pérez P. (2014). Cdc42 regulates polarized growth and cell integrity in fission yeast. Biochem. Soc. Trans..

[3] Martin S.G., Arkowitz R.A. (2014). Cell polarization in budding and fission yeasts. FEMS Microbiol. Rev..

[4] Martin S.G. (2015). Spontaneous cell polarization: feedback control of Cdc42 GTPase breaks cellular symmetry. Bioessays.

[5] Bendezú F.O., Martin S.G. (2011). Actin cables and the exocyst form two independent morphogenesis pathways in the fission yeast. Mol. Biol. Cell.

[6] Bendezú F.O., Vincenzetti V., Vavylonis D., Wyss R., Vogel H., Martin S.G. (2015). Spontaneous Cdc42 polarization independent of GDI-mediated extraction and actin-based trafficking. PLoS Biol..

[7] Jaquenoud M., Peter M. (2000). Gic2p may link activated Cdc42p to components involved in actin polarization, including Bni1p and Bud6p (Aip3p). Mol. Cell. Biol..

[8] Tong Z., Gao X.D., Howell A.S., Bose I., Lew D.J., Bi E. (2007). Adjacent positioning of cellular structures enabled by a Cdc42 GTPase-activating protein-mediated zone of inhibition. J. Cell Biol..

[9] Tatebe H., Nakano K., Maximo R., Shiozaki K. (2008). Pom1 DYRK regulates localization of the Rga4 GAP to ensure bipolar activation of Cdc42 in fission yeast. Curr. Biol..

[10] Kelly F.D., Nurse P. (2011). Spatial control of Cdc42 activation determines cell width in fission yeast. Mol. Biol. Cell.

[11] Wedlich-Soldner R., Altschuler S., Wu L., Li R. (2003). Spontaneous cell polarization through actomyosin-based delivery of the Cdc42 GTPase. Science.

[12] Marco E., Wedlich-Soldner R., Li R., Altschuler S.J., Wu L.F. (2007). Endocytosis optimizes the dynamic localization of membrane proteins that regulate cortical polarity. Cell.

[13] Orlando K., Sun X., Zhang J., Lu T., Yokomizo L., Wang P., Guo W. (2011). Exo-endocytic trafficking and the septin-based diffusion barrier are required for the maintenance of Cdc42p polarization during budding yeast asymmetric growth. Mol. Biol. Cell.

[14] Gachet Y., Tournier S., Millar J.B., Hyams J.S. (2001). A MAP kinase-dependent actin checkpoint ensures proper spindle orientation in fission yeast. Nature.

[15] Millar J.B., Buck V., Wilkinson M.G. (1995). Pyp1 and Pyp2 PTPases dephosphorylate an osmosensing MAP kinase controlling cell size at division in fission yeast. Genes Dev..

[16] Shiozaki K., Russell P. (1995). Cell-cycle control linked to extracellular environment by MAP kinase pathway in fission yeast. Nature.

[17] Kato T., Okazaki K., Murakami H., Stettler S., Fantes P.A., Okayama H. (1996). Stress signal, mediated by a Hog1-like MAP kinase, controls sexual development in fission yeast. FEBS Lett..

[18] Brewster J.L., Gustin M.C. (2014). Hog1: 20 years of discovery and impact. Sci. Signal..

[19] Saito H., Posas F. (2012). Response to hyperosmotic stress. Genetics.

[20] Cuadrado A., Nebreda A.R. (2010). Mechanisms and functions of p38 MAPK signalling. Biochem. J..

[21] Zimmerman S., Daga R.R., Chang F. (2004). Intra-nuclear microtubules and a mitotic spindle orientation checkpoint. Nat. Cell Biol..

[22] Sawin K.E., Lourenco P.C., Snaith H.A. (2004). Microtubule nucleation at non-spindle pole body microtubule-organizing centers requires fission yeast centrosomin-related protein mod20p. Curr. Biol..

[23] Meadows J.C., Millar J. (2008). Latrunculin A delays anaphase onset in fission yeast by disrupting an Ase1-independent pathway controlling mitotic spindle stability. Mol. Biol. Cell.

[24] Riedl J., Crevenna A.H., Kessenbrock K., Yu J.H., Neukirchen D., Bista M., Bradke F., Jenne D., Holak T.A., Werb Z. (2008). Lifeact: a versatile marker to visualize F-actin. Nat. Methods.

[25] Huang J., Huang Y., Yu H., Subramanian D., Padmanabhan A., Thadani R., Tao Y., Tang X., Wedlich-Soldner R., Balasubramanian M.K. (2012). Nonmedially assembled F-actin cables incorporate into the actomyosin ring in fission yeast. J. Cell Biol..

[26] Pérez P., Cansado J. (2010). Cell integrity signaling and response to stress in fission yeast. Curr. Protein Pept. Sci..

[27] Smith D.A., Morgan B.A., Quinn J. (2010). Stress signalling to fungal stress-activated protein kinase pathways. FEMS Microbiol. Lett..

[28] Gaits F., Degols G., Shiozaki K., Russell P. (1998). Phosphorylation and association with the transcription factor Atf1 regulate localization of Spc1/Sty1 stress-activated kinase in fission yeast. Genes Dev..

[29] He B., Guo W. (2009). The exocyst complex in polarized exocytosis. Curr. Opin. Cell Biol..

[30] Goode B.L., Eskin J.A., Wendland B. (2015). Actin and endocytosis in budding yeast. Genetics.

[31] Bishop A.C., Ubersax J.A., Petsch D.T., Matheos D.P., Gray N.S., Blethrow J., Shimizu E., Tsien J.Z., Schultz P.G., Rose M.D. (2000). A chemical switch for inhibitor-sensitive alleles of any protein kinase. Nature.

[32] Zuin A., Carmona M., Morales-Ivorra I., Gabrielli N., Vivancos A.P., Ayté J., Hidalgo E. (2010). Lifespan extension by calorie restriction relies on the Sty1 MAP kinase stress pathway. EMBO J..

[33] Wilkinson M.G., Samuels M., Takeda T., Toone W.M., Shieh J.C., Toda T., Millar J.B., Jones N. (1996). The Atf1 transcription factor is a target for the Sty1 stress-activated MAP kinase pathway in fission yeast. Genes Dev..

[34] Shiozaki K., Russell P. (1996). Conjugation, meiosis, and the osmotic stress response are regulated by Spc1 kinase through Atf1 transcription factor in fission yeast. Genes Dev..

[35] Kanoh J., Watanabe Y., Ohsugi M., Iino Y., Yamamoto M. (1996). *Schizosaccharomyces pombe* gad7^+^ encodes a phosphoprotein with a bZIP domain, which is required for proper G1 arrest and gene expression under nitrogen starvation. Genes Cells.

[36] Petersen J., Hagan I.M. (2005). Polo kinase links the stress pathway to cell cycle control and tip growth in fission yeast. Nature.

[37] Grallert A., Patel A., Tallada V.A., Chan K.Y., Bagley S., Krapp A., Simanis V., Hagan I.M. (2013). Centrosomal MPF triggers the mitotic and morphogenetic switches of fission yeast. Nat. Cell Biol..

[38] Shiozaki K., Shiozaki M., Russell P. (1998). Heat stress activates fission yeast Spc1/StyI MAPK by a MEKK-independent mechanism. Mol. Biol. Cell.

[39] Lawrence C.L., Maekawa H., Worthington J.L., Reiter W., Wilkinson C.R., Jones N. (2007). Regulation of *Schizosaccharomyces pombe* Atf1 protein levels by Sty1-mediated phosphorylation and heterodimerization with Pcr1. J. Biol. Chem..

[40] Sansó M., Gogol M., Ayté J., Seidel C., Hidalgo E. (2008). Transcription factors Pcr1 and Atf1 have distinct roles in stress- and Sty1-dependent gene regulation. Eukaryot. Cell.

[41] Yamamoto M., Imai Y., Watanabe Y., Pringle J.R., Broach J.R., Jones E.W. (1997). Mating and sporulation in *Schizosaccharomyces pombe*. The Molecular and Cellular Biology of the Yeast *Saccharomyces*, Volume 3: Cell Cycle and Cell Biology.

[42] Harigaya Y., Yamamoto M. (2007). Molecular mechanisms underlying the mitosis-meiosis decision. Chromosome Res..

[43] Su S.S., Tanaka Y., Samejima I., Tanaka K., Yanagida M. (1996). A nitrogen starvation-induced dormant G0 state in fission yeast: the establishment from uncommitted G1 state and its delay for return to proliferation. J. Cell Sci..

[44] Bendezú F.O., Martin S.G. (2013). Cdc42 explores the cell periphery for mate selection in fission yeast. Curr. Biol..

[45] Sajiki K., Hatanaka M., Nakamura T., Takeda K., Shimanuki M., Yoshida T., Hanyu Y., Hayashi T., Nakaseko Y., Yanagida M. (2009). Genetic control of cellular quiescence in *S. pombe*. J. Cell Sci..

[46] Yanagida M., Ikai N., Shimanuki M., Sajiki K. (2011). Nutrient limitations alter cell division control and chromosome segregation through growth-related kinases and phosphatases. Philos. Trans. R. Soc. Lond. B Biol. Sci..

[47] Reiser V., Raitt D.C., Saito H. (2003). Yeast osmosensor Sln1 and plant cytokinin receptor Cre1 respond to changes in turgor pressure. J. Cell Biol..

[48] Harrison J.C., Bardes E.S., Ohya Y., Lew D.J. (2001). A role for the Pkc1p/Mpk1p kinase cascade in the morphogenesis checkpoint. Nat. Cell Biol..

