# Supplementary material for: Remodeling of the Fission Yeast Cdc42 Cell-Polarity Module via the Sty1 p38 Stress-Activated Protein Kinase Pathway
Source: Curr Biol. 2016 Nov 7;26(21):2921–8. doi: 10.1016/j.cub.2016.08.048 (PMC5106388; doi:10.1016/j.cub.2016.08.048)
Supplement: Document S1. Supplemental Experimental Procedures and Figures S1–S3 [file mmc1.pdf]

Current Biology, Volume *26*

Supplemental Information

Remodeling of the Fission Yeast Cdc42 Cell-Polarity Module via the Sty1 p38 Stress-Activated Protein Kinase Pathway

Delyan R. Mutavchiev, Marcin Leda, and Kenneth E. Sawin

Figure S1

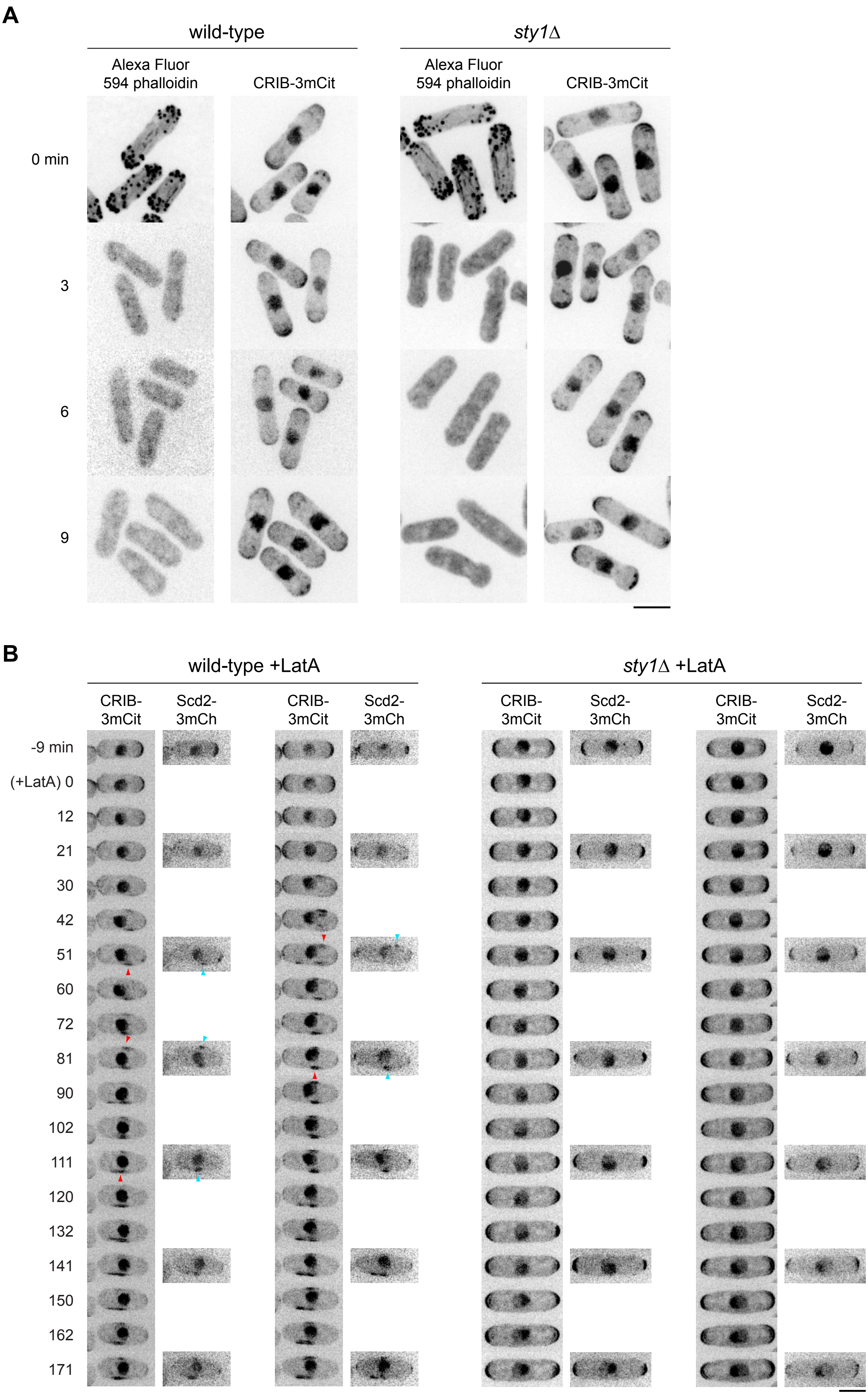
A
wild-type
sty1Δ
Alexa Fluor 594 phalloidin
CRIB-3mCit
0 min
3
6
9
B
wild-type +LatA
sty1Δ +LatA
CRIB-3mCit
Scd2-3mCh
-9 min
(+LatA) 0
12
21
30
42
51
60
72
81
90
102
111
120
132
141
150
162
171

Figure S1 (related to Figures 1 and 2). LatA-induced actin depolymerization and dispersal of the Cdc42 polarity module.

A. Latrunculin A (LatA)-induced actin depolymerization in wild-type and *sty1*Δ cells, shown by Alexa Fluor 594 phalloidin staining of fixed cells, rather than by Lifeact-mCherry in live cells (which is shown in Figures 1 and 2). CRIB-3xmCitrine (CRIB-3mCit) fluorescence in the same cells is also shown. Times shown are relative to addition of LatA. These control experiments demonstrate that the presence/absence of Lifeact-mCherry does not alter the extent or rate of actin depolymerization by LatA. Because fixation and staining methods are optimized for actin preservation [S1], CRIB localization and cell morphology may be slightly altered relative to live cells.

B. Still images from movies of CRIB-3mCit and the Cdc42 polarity module scaffold protein Scd2-3xmCherry (Scd2-3mCh) in two wild-type cells and two *sty1*Δ cells after addition of 50 μM LatA. Times shown are relative to addition of LatA. Red and blue arrowheads indicate examples of ectopic patches of CRIB and Scd2, respectively, which co-localize. Together with previous work showing similar dispersal and patches for Scd2-3xGFP and for Cdc42 itself in response to LatA treatment [S2, S3], this co-localization demonstrates that CRIB dispersal and patches indicates re-localization of the Cdc42 polarity module at large rather than a specific disruption of the interaction between CRIB and the Cdc42 module. Because Scd2-3mCh is extremely faint and sensitive to photobleaching, it was imaged only at 30 min intervals, and Z-series in both channels (i.e. CRIB-3mCit and Scd2-3mCh) were acquired at 1 μm intervals.

Scale bars, 5 μm.

Figure S2

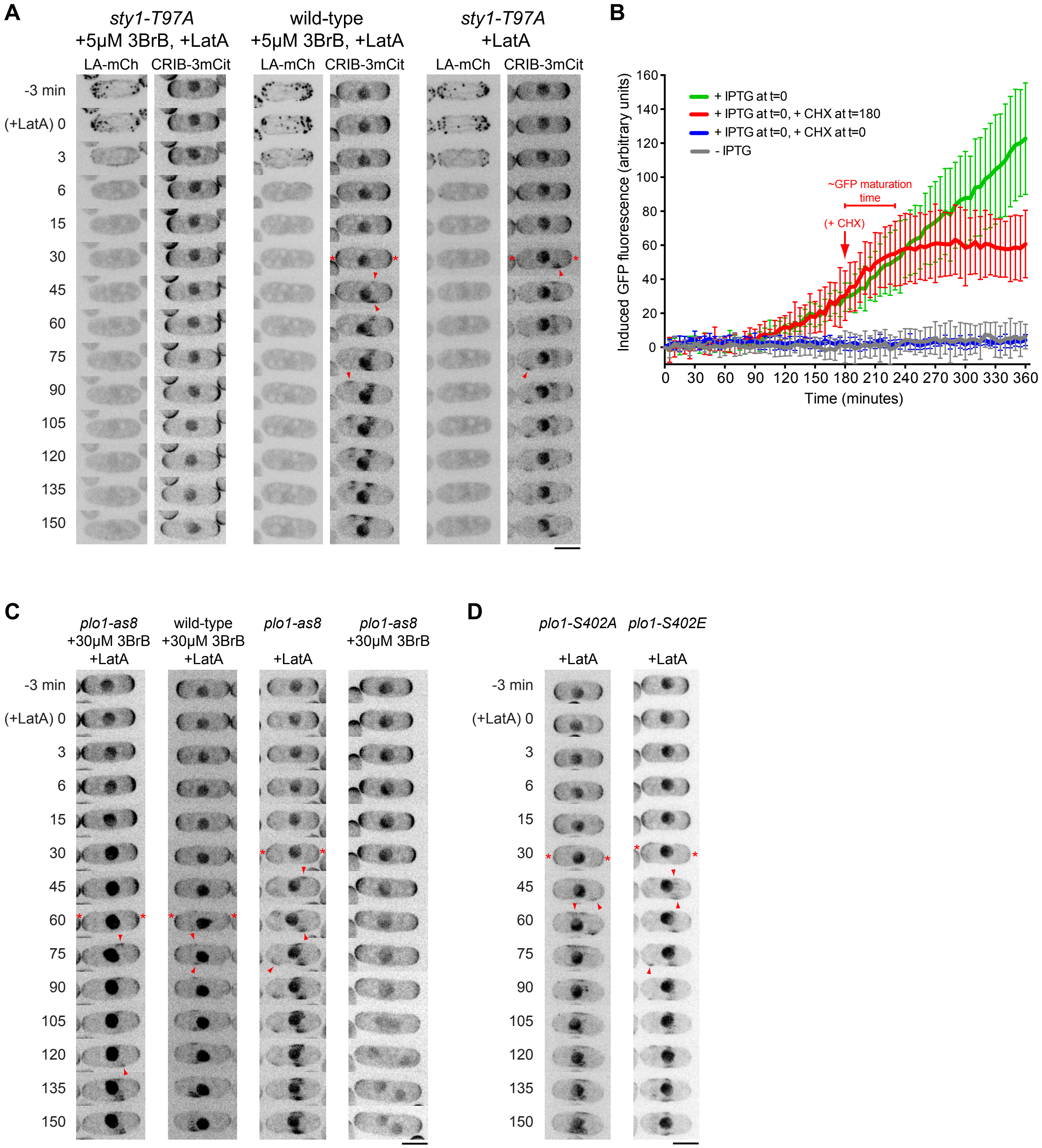

A
sty1-T97A +5μM 3BrB, +LatA
wild-type +5μM 3BrB, +LatA
sty1-T97A +LatA
LA-mCh
CRIB-3mCit
-3 min
(+LatA) 0
3
6
15
30
45
60
75
90
105
120
135
150
B
+ IPTG at t=0
+ IPTG at t=0, + CHX at t=180
+ IPTG at t=0, + CHX at t=0
- IPTG
~GFP maturation time
(+ CHX)
Induced GFP fluorescence (arbitrary units)
Time (minutes)
C
plo1-as8 +30μM 3BrB +LatA
wild-type +30μM 3BrB +LatA
plo1-as8 +LatA
plo1-as8 +30μM 3BrB
D
plo1-S402A +LatA
plo1-S402E +LatA

Figure S2 (related to Figure 2). Role of Sty1 in LatA-induced CRIB dispersal.

A. Control experiments showing that inhibition of LatA-induced CRIB dispersal by 3-BrB-PP1 in analog-sensitive *sty1-T97A* cells requires both the *sty1-T97A* allele and 3-BrB-PP1. Still images from movies of Lifeact-mCherry (LA-mCh) and CRIB-3xmCitrine (CRIB-3mCit) in: *sty1-T97A* cells and wild-type cells pre-treated with 5 μM 3-BrB-PP1 (3BrB) for 10 min, prior to addition of 50 μM LatA in the continued presence of 3BrB; and in *sty1-T97A* cells not pre-treated with 3BrB. Cell in left-most panel is reproduced from Figure 2C, with additional time-points. Asterisks indicate dispersal of CRIB from cell tips. Arrowheads indicate examples of ectopic CRIB patches after dispersal. Times shown are relative to addition of LatA.

B. Evidence that 100 μg/mL cycloheximide (CHX) completely inhibits protein synthesis in fission yeast. This provides additional controls for the experiments shown in Figure 2E. Wild-type cells containing an isopropyl β-D-1-thiogalactopyranoside (IPTG)-inducible *GFP* gene were assayed for GFP fluorescence under the conditions shown. Addition of IPTG leads to a large increase in fluorescence over time (green curve), but no increase occurs if CHX is added simultaneously with IPTG (blue curve). If CHX is added after the increase of fluorescence has begun (red curve), fluorescence continues to increase for about 45 min, which should correspond to the maturation time of already-synthesized GFP [S4]; however, subsequently there is no further increase. Error bars indicate SD.

C. Inhibition of polo kinase Plo1 does not affect LatA-induced CRIB dispersal. Still images from movies of CRIB-3xmCitrine in: analog-sensitive *plo1-as8* cells and wild-type cells pre-treated with 30 μM 3-BrB-PP1 (3BrB) for 10 min, prior to addition of 50 μM LatA in the continued presence of 3BrB; in *plo1-as8* cells not pre-treated with 3BrB prior to LatA addition; and in *plo1-as8* cells treated with 3BrB but not with LatA. Times shown are relative to addition of LatA. Asterisks indicate dispersal of CRIB from cell tips. Arrowheads indicate examples of ectopic CRIB patches after dispersal. In both *plo1-as8* and wild-type cells pre-treated with 30 μM 3BrB, CRIB dispersal and ectopic patch formation occur later than in non-pre-treated cells (see Figures 1A and S1B), and also later than in cells pre-treated with lower concentrations of 3BrB (see Figure S2A). This was reproducibly observed and may be due to weak off-target effects resulting from the high concentration of analog required to inhibit Plo1 [S5]. Right-most panel shows that addition of 30 μM 3BrB to *plo1-as8* cells prevents cytokinesis/septation, a characteristic feature of *plo1* loss-of-function [S6].

D. Mutation of a Sty1-dependent phosphorylation site (Ser 402) in Plo1 does not affect LatA-induced CRIB dispersal. Still images from movies of CRIB-3xmCitrine (CRIB-3mCit) in non-phosphorylatable *plo1-S402A* and phosphomimetic *plo1-S402E* cells [S7] after addition of 50 μM LatA. Times shown are relative to addition of LatA. Asterisks indicate dispersal of CRIB from cell tips. Arrowheads indicate examples of ectopic CRIB patches after dispersal. Note that in *plo1-S402A* and *plo1-S402E* cells, LatA-induced CRIB dispersal is indistinguishable from that observed in wild-type cells (see Figures 1A and S1B, and Movie S1), as well as that observed both in non-inhibited *plo1-as8* cells (Figure S2C) and in analog-inhibited *plo1-as8* cells (apart from the small 3BrB-dependent delay described above; Figure S2C). This is important because Ser 402 of Plo1 has been shown to be phosphorylated in a Sty1-dependent manner during recovery after heat stress (although this residue is not phosphorylated after oxidative or osmotic stress), and S402 phosphorylation has further been implicated to play a role in regulation of cell elongation after heat stress [S7]. However, our experiments shown here demonstrate that Plo1 Ser 402 does not play a role in CRIB dispersal after LatA treatment.

Scale bars, 5 μm.

Figure S3

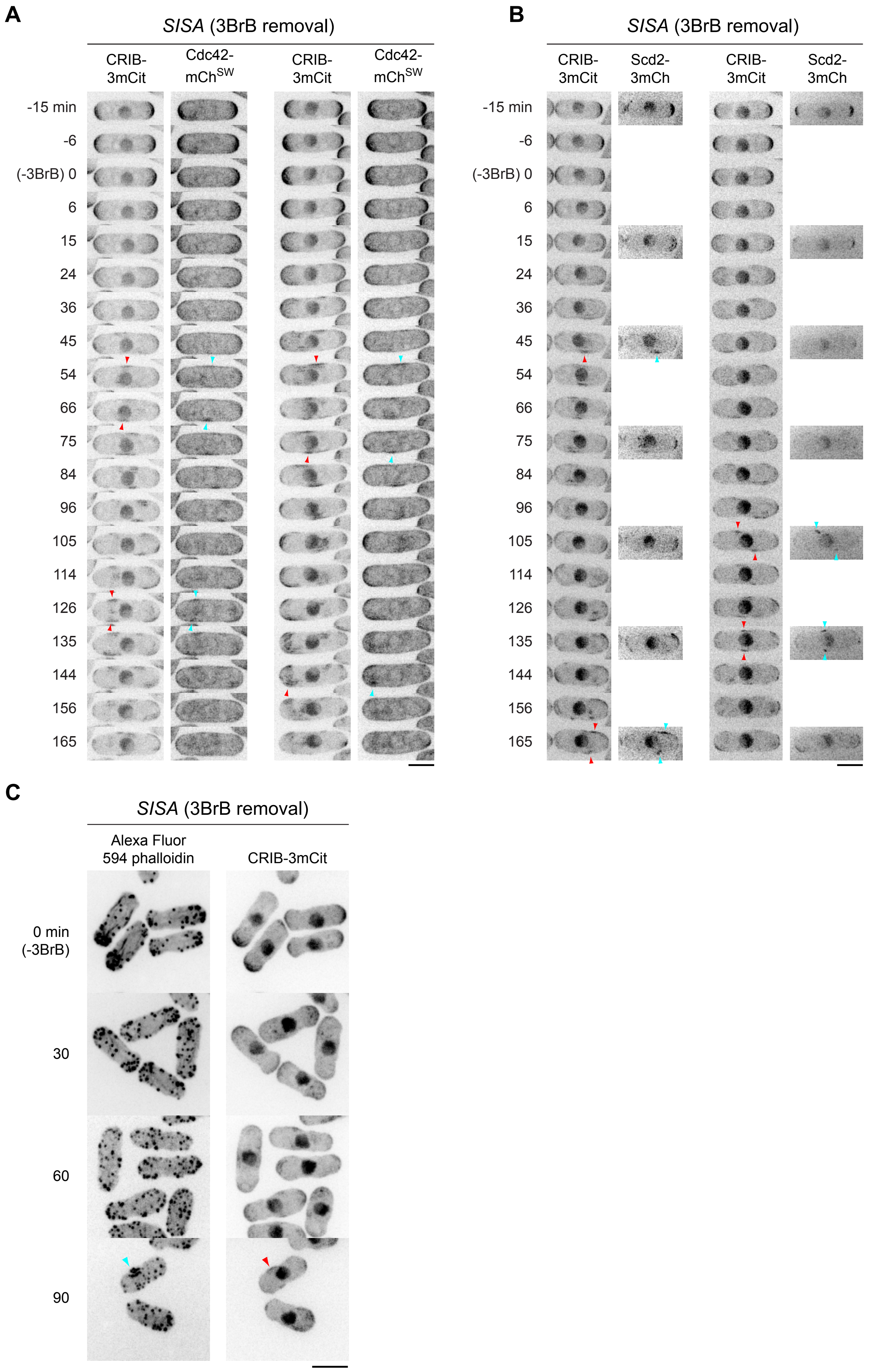
A
SISA (3BrB removal)
CRIB-3mCit
Cdc42-mCh^SW
CRIB-3mCit
Cdc42-mCh^SW
-15 min
-6
(-3BrB) 0
6
15
24
36
45
54
66
75
84
96
105
114
126
135
144
156
165
B
SISA (3BrB removal)
CRIB-3mCit
Scd2-3mCh
CRIB-3mCit
Scd2-3mCh
-15 min
-6
(-3BrB) 0
6
15
24
36
45
54
66
75
84
96
105
114
126
135
144
156
165
C
SISA (3BrB removal)
Alexa Fluor 594 phalloidin
CRIB-3mCit
0 min (-3BrB)
30
60
90

Figure S3 (related to Figure 3). Remodeling of cell polarity after Sty1 activation in *SISA* cells.

A. Still images from movies of CRIB-3mCit and internally ("sandwich")-tagged Cdc42-mCherry (Cdc42-mCh^{SW}; [S3]) in two *SISA* cells after Sty1 activation by removal of ATP-competitive analog 3-BrB-PP1 (3BrB). Times shown are relative to 3BrB removal. Red and blue arrowheads indicate examples of ectopic patches of CRIB and Cdc42, respectively, which co-localize. This co-localization demonstrates that CRIB dispersal and patches in Sty1-activated *SISA* cells indicates re-localization of the Cdc42 polarity module at large rather than a specific disruption of the interaction between CRIB and the Cdc42 module.

B. Still images from movies of CRIB-3mCit and the Cdc42 polarity module scaffold protein Scd2-3xmCherry (Scd2-3mCh) in two *SISA* cells after Sty1 activation by removal of 3BrB. Times shown are relative to 3BrB removal. Red and blue arrowheads indicate examples of ectopic patches of CRIB and Scd2, respectively, which co-localize. Together with results shown in Figure S3A, this co-localization demonstrates that CRIB dispersal and patches in Sty1-activated *SISA* cells indicates re-localization of the Cdc42 polarity module at large rather than a specific disruption of the interaction between CRIB and the Cdc42 module. Because Scd2-3mCh is extremely faint and sensitive to photobleaching, it was imaged only at 30 min intervals, and Z-series in both channels (i.e. CRIB-3mCit and Scd2-3mCh) were acquired at 1 μm intervals.

C. Depolarization of the actin cytoskeleton after Sty1 activation in *SISA* cells (triggered by removal of 3BrB), shown by Alexa Fluor 594 phalloidin staining of fixed cells, rather than by Lifeact-mCherry (LA-mCh) in live cells (which is shown in Figure 3). CRIB-3xmCitrine (CRIB-3mCit) fluorescence in the same cells is also shown. Times shown are relative to 3BrB removal. This control experiment demonstrates that actin depolarization in Sty1-activated *SISA* cells is not dependent on the presence of LA-mCh. Also note that clusters of cortical actin patches (blue arrowhead) are often associated with ectopic CRIB patches (red arrowhead). Because fixation and staining methods are optimized for actin preservation [S1], CRIB localization and cell morphology may be slightly altered relative to live cells.

Scale bars, 5 μm.

Supplemental Experimental Procedures

Yeast strain construction and cultures

Standard fission yeast techniques were used throughout for cell culture and genetic crosses [S8]. Cells were grown, as indicated, in: YE5S rich medium (using Difco yeast extract); EMM2 minimal medium; low-nitrogen (low-N) EMM2 minimal medium, which contains 0.5 g/L ammonium chloride instead of 5 g/L; EMM2-N, which is identical to EMM2 but lacks ammonium chloride altogether; or PMG minimal medium, which is identical to EMM2 but contains 4 g/L sodium glutamate instead of ammonium chloride as nitrogen source. Nutritional supplements were used at 175 mg/L. Solid media used 2% Difco Bacto agar. When working with *SISA* strains, to prevent activation of Sty1, 3BrB-PP1 (see below) was added to liquid media (5μM 3BrB-PP1 final concentration) and to solid media (5μM, or 30μM 3BrB-PP1 when waking strains, to prevent possible suppressor mutations). Mating for genetic crosses was performed on SPAS plates with nutritional supplements at 45 mg/L [S8]. Crosses were performed using tetrad dissection or random spore analysis, with confirmation by yeast colony PCR as appropriate. Tagging and deletion of genes were performed using PCR-based methods [S9]. Strains used in this study are listed below.

To generate the CRIB-3xmCitrine strain, CRIB and 3xmCitrine nucleic acid sequences were synthesized as two separate fragments by GeneArt (ThermoFisher). For the CRIB component, the sequence encoding the CRIB domain of the *S. cerevisiae* Gic2 protein (amino acids 1-208, with an intentional mutation W23A; [S10, S11]) was codon-optimized for *S. pombe* expression, and additional sequence was added to the 3' end to encode a C-terminal linker GDGAGLIN. The CRIB-plus-linker fragment was cloned into pMA-T (GeneArt) to generate the plasmid pKS1296. For the 3xmCitrine component, codon usage was varied in each of the three mCitrine sequences, so that each mCitrine nucleic acid sequence is only ~70% identical to the other two, although all three encode the same amino-acid sequence. This decreases the likelihood of undesired recombination occurring between tandem mCitrine sequences during genetic crosses. The 3xmCitrine fragment was cloned into pMK-RQ (GeneArt) to generate plasmid pKS1295. The 3xmCitrine fragment was excised from pKS1295 using SalI and BamHI and cloned into SalI and BamHI sites in pKS1296, placing the 3xmCitrine tag downstream of and in frame with the CRIB-plus-linker fragment, to generate plasmid pKS1305. Next, the entire CRIB-3xmCitrine fusion gene was excised from pKS1305 using NdeI and BamHI and cloned into NdeI and BamHI sites in the vector pRAD13 (kind gift from Y. Watanabe) which places the fusion gene under control of the *adh13* promoter (a weakened *adh1* promoter; [S12]), to generate plasmid pKS1374. pKS1374 was linearized using BlpI to allow for stable integration into the fission yeast *ars1* locus, using LEU2 as a selection marker. Cells expressing the *adh13*:CRIB-3xmCitrine fusion protein do not have any morphological defects. Like the previously published CRIB-3xGFP fusion protein [S13], the CRIB-3xmCitrine fusion protein shows some localization to the nucleus; this nuclear localization has been shown to be an artifact unrelated to Cdc42 [S3]. Use of a weaker promoter did not alter the CRIB-3xmCitrine nuclear localization.

To generate a strain with IPTG-inducible GFP expression, we used the plasmid pSK173 ([S14]; see important erratum, doi: 10.1002/yea.3136). This plasmid, also known as nmt::lacO*-GFP, contains a copy of GFP under the control of the thiamine-repressible *nmt1* promoter, with a lacO operator sequence inserted five base pairs downstream of the *nmt1* TATA box, as well as a copy of the lac repressor (lacI) fused to a nuclear localization sequence, under the control of an SV40 promoter. pSK173 was linearized using AfeI and stably integrated into the *ars1* genomic locus using *ura4+* as a selection marker.

Strains used in this study:

**Strain**	**Genotype**	**Source**
KS7305	*h- ars1(Blp*I*):*P*adh13:CRIB-3xmCitrine:LEU2 ade6-M216 leu1-32 ura4-D18*	This study
KS7311	*h- sty1Δ::kanMX ars1(Blp*I*):*P*adh13:CRIB-3xmCitrine:LEU2 ade6-M216 leu1-32 ura4-D18*	This study
KS7566	*h+ ars1(Blp*I*):*P*adh13:CRIB-3xmCitrine:LEU2* P*act1:lifeact-mCherry::leu1+ ade6-M216 leu1-32 ura4-D18*	This study
KS7660	*h+ sty1Δ::kanMX ars1(Blp*I*):*P*adh13:CRIB-3xmCitrine:LEU2* P*act1:lifeact-mCherry::leu1+ ade6-M210 leu1-32 ura4-D18*	This study
KS7737	*h- ars1(Blp*I*):*P*adh13:CRIB-3xmCitrine:LEU2 atf1Δ::kanMX6 ade6-M216 leu1-32 ura4-D18*	This study
KS7830	*h+ sty1-T97A ars1(Blp*I*):*P*adh13:CRIB-3xmCitrine:LEU2*	This study

	Pact1:lifeact-mCherry::leu1+ ade6-M216 leu1-32 ura4-D18	
KS8116	*h- ars1(AfeI):nmt:lacO*:GFP:lacI-NLS:ura4+ ade6-M216 leu1-32 ura4-D18*	This study
KS8140	*h- sty1-mECitrine:KanMX6 ade6-M210 leu1-32 ura4-D18*	This study
KS8164	*h- ars1(BlpI):*P*adh13:CRIB-3xmCitrine:LEU2*	This study
KS8217	*h- ars1(BlpI):*P*adh13:CRIB-3xmCitrine:LEU2 plo1.as8:ura4+*	This study
KS8226	*h+ sty1-T97A wis1DD:12myc:ura4+ pyp1Δ::ura4+ pyp2Δ::LEU2 ars1(BlpI):*P*adh13:CRIB-3xmCitrine:LEU2 leu1-32 ura4-D18*	This study
KS8276	*h- wis1Δ::ura4+ ars1(BlpI):*P*adh13:CRIB-3xmCitrine:LEU2 ade6-M210 leu1-32 ura4-D18*	This study
KS8311	*h+ sty1-T97A wis1DD:12myc:ura4+ pyp1Δ::ura4+ pyp2Δ::LEU2 ars1(BlpI):*P*adh13:CRIB-3xmCitrine:LEU2* P*act1:lifeact-mCherry::leu1+ leu1-32 ura4-D18*	This study
KS 8323	*h- sty1-T97A ars1(BlpI):*P*adh13:CRIB-3xmCitrine:LEU2*	This study
KS8332	*h- sty1-T97A wis1DD:12myc:ura4+ pyp1Δ::ura4+ pyp2Δ::LEU2 ars1(BlpI):*P*adh13:CRIB-3xmCitrine:LEU2 cdc42-mCherry*^{SW}*:kanMX6 leu1-32 ura4-D18*	This study
KS8764	*h- Scd2-3xmCherry:kanMX6 ars1(BlpI):*P*adh13:CRIB-3xmCitrine:LEU2 ade6-M216 leu1-32 ura4-D18*	This study
KS8765	*h- Scd2-3xmCherry:kanMX6 sty1Δ::kanMX ars1(BlpI):*P*adh13:CRIB-3xmCitrine:LEU2 ade6-M216 leu1-32 ura4-D18*	This study
KS8773	*h+ sty1-T97A wis1DD:12myc:ura4+ pyp1Δ::ura4+ pyp2Δ::LEU2 ars1(BlpI):*P*adh13:CRIB-3xmCitrine:LEU2*	This study
KS8779	*h- plo1-S402A ars1(BlpI):*P*adh13:CRIB-3xmCitrine:LEU2*	This study
KS8782	*h+plo1-S402E ars1(BlpI):*P*adh13:CRIB-3xmCitrine:LEU2*	This study

Base strains and strains from other laboratories:

**Strain**	**Genotype**	**Source**
KS1	*h-*	Lab stock
KS2	*h+*	Lab stock
KS515	*h+ ade6-M210 leu1-32 ura4-D18*	Lab stock
KS516	*h- ade6-M216 leu1-32 ura4-D18*	Lab stock
MBY6843 (KS6696)	*h+* P*act1:lifeact-mCherry::leu1+ ade6-M216 leu1-32 ura4-D18*	M. Balasubramanian
AV15 (KS7644)	*h- atf1Δ::kanMX6*	E. Hidalgo
AZ107 (KS7671)	*h+ sty1-T97A ura4-D18*	E. Hidalgo
KS2086 (KS7902)	*h- wis1DD:12myc::ura4+ leu1-32 ura4-D18*	K. Shiozaki
IH10368 (KS7918)	*h- plo1.as8:ura4+ ura4-D18*	I. Hagan
IH3758 (KS8735)	*h- plo1.S402A*	I. Hagan
IH3759 (KS8736)	*h- plo1.S402E*	I. Hagan
JM 281 (KS 7944)	*h- pyp1Δ::ura4+ leu1-32 ura4-D18*	J. Millar
JM 655 (KS 7945)	*h+ pyp2Δ::LEU2 his7-366 leu1-32 ura4-D18*	J. Millar
YSM2446 (KS8284)	*h- cdc42-mCherry*^{SW}*:kanMX6*	S. Martin

Physiological experiments, cell lysate preparation and western blotting

For all physiological experiments, cells were grown in a shaking water bath at 25°C. Latrunculin A (LatA) was obtained from either Alpha Laboratories (129-04361) or Abcam (ab144290) and dissolved in DMSO to make a 5 mM stock solution. 3-BrB-PP1 (4-Amino-1-tert-butyl-3-(3-bromobenzyl)pyrazolo[3,4-d]pyrimidine; A602985) was obtained from Toronto Research Chemicals and dissolved in methanol to make a 50 mM stock solution. Stock solutions were stored -80°C until use.

For growth curves of the *SISA* strain, cells were initially grown to a cell density of 2 x 10^6/mL in YE5S containing 5µM 3BrB-PP1. Cells were then filtered through a 0.45 µm membrane filter (Millipore, HVLP04700). Filters were then either transferred to one culture volume of YE5S containing 5µM 3BrB-PP1, or washed twice with YE5S (one culture volume per wash) and then transferred to one culture volume of YE5S. The resuspended cultures were returned to the water bath and samples were collected hourly. Each sample was fixed by addition of 37% formaldehyde (formalin) solution to a final concentration of 3.7% formaldehyde. Dilutions of the fixed samples were measured using Z2 Coulter Particle counter (Beckman-Coulter) using a 4-12 µm particle range setting.

To prepare cell lysates for measuring Sty1 activation on western blots after stress, cell cultures were grown in YE5S to a cell density of 1.25 x 10^7 cells/mL. Next, DMSO (1% final), LatA (50 µM final, with 1% DMSO final), or KCl (0.6 M final, from 3M stock in YE5S) was pipetted and mixed into the cell cultures. Samples of 1.6 mL (or 1.9 mL for KCl, because of increased volume) were collected into 2 mL screw-cap microcentrifuge tubes at the indicated times and immediately centrifuged at 4000 rpm at 4°C for 1 min. The supernatant was quickly removed and cell pellets placed on ice. Cell pellets were resuspended in 1 mL ice-cold 10mM $NaPO_4$ 0.5mM EDTA pH 7.5 buffer and centrifuged again at 13,000 rpm at 4°C for 1 min. The supernatant was removed, and the cell pellets were flash-frozen in liquid nitrogen and stored at -80°C until further processing. For cell lysis, 0.5 mm zirconium/silica beads pre-chilled to -20°C (BioSpec; 11079105z)) were added to the cell pellet, together with 40 µL ice-cold lysis buffer containing: 150 mM NaCl, 20 mM Tris-HCl pH 7.5, 0.05% Triton X-100, 10 µg/mL each of 'CLAAPE' protease inhibitors (chymostatin, leupeptin, antipain, pepstatin, E64), 2 mM DTT, 6 mM $MgCl_2$ 1 mM PMSF, 1 mM Benzamidine, 50 mM β-glycerophosphate, 50 mM EDTA, 1 mM NaF, 50 nM Calyculin A, 50 nM Okadaic acid, 100 nM Na_3VO_4, 2 mM AEBSF, 10% Glycerol. The suspension was put in a Ribolyser bead-beater (Hybaid) and four cycles of bead-beating were run: one cycle of 60 sec and three subsequent cycles of 30 sec each, all at speed setting "4". Between each cycle, samples were chilled in an ice-water slurry for 2 min. After bead-beating, the lysate in the screw-cap tubes was recovered by puncturing the tubes and centrifuging into a second tube. Cell lysates were then clarified by centrifugation at 13,000 rpm at 4°C for 10 min. One-third volume of 4X Laemmli sample buffer was then added to clarified lysates, which were then boiled at 100°C for 5 min and flash-frozen in liquid nitrogen.

To prepare *SISA* cell lysates for measuring Atf1 on western blots, parallel cell cultures were grown in YE5S containing 5µM 3BrB-PP1 to a cell density of 1.25 x 10^7 cells/mL. Cells were then filtered as described above, and washed twice, either with YE5S (one culture volume per wash) or with YE5S containing 5µM 3BrB-PP1 (one culture volume per wash), as appropriate. Cells were then resuspended in one culture volume of YE5S or YE5S containing 5µM 3BrB-PP1, as appropriate, and returned to the water bath. Samples of 1.9 mL were collected into 2 mL screw-cap microcentrifuge tubes at the indicated time points and processed as described above.

Cell lysates were separated by SDS-PAGE and transferred by western blotting to 0.2 µm nitrocellulose filters (Biorad). Western blots were first stained with Ponceau S and scanned to normalize for protein content between lanes. For measuring Sty1 activation, blots were probed with monoclonal rabbit anti-phospho-p38 primary antibody (Cell Signaling Technology; #4511) and IRDye 800CW Donkey anti-Rabbit secondary antibody (Licor; 925-32213). For measuring Atf1, blots were probed with monoclonal mouse anti-ATF1 primary antibody (Abcam; ab18123) and IRDye 800CW Donkey anti-Mouse secondary antibody (Licor; 925-32212). Blots were imaged using an Odyssey fluorescence imager (Licor) and quantified using Image Studio Lite (Licor). Ponceau S scans of blots were converted to 8-bit images using ImageJ and imported into Image Studio Lite for quantification.

Microscopy sample preparation and imaging

All imaging experiments were performed with exponentially growing cells grown at 25°C. Experiments were performed in YE5S medium, with the exception of: nitrogen-starvation (N-starvation) experiments, which were performed in low-N EMM2 and EMM2-N; and GFP IPTG-induction experiments, which were performed in PMG, with glucose added to the medium after autoclaving.

For preparation of imaging slides, coverslip dishes (MatTek; P35G-0.170-14-C.s) or 4-chamber glass bottom micro-slides (Ibidi; 80427) were placed on a 25°C heat block, coated with 1mg/mL soybean lectin (Sigma; L1395), left for 10 min, and washed with appropriate medium to remove excess lectin. Cell cultures were added to dishes/slides and left to settle for 15 minutes. Next, the cells were washed extensively with appropriate media using aspiration with at least 3 full exchanges of media (at least 1 mL each). Finally, 400µL (or 600 µL for N-starvation) of medium was used to cover the cells and the preparations were quickly placed in the microscope chamber at 25°C.

Live-cell fluorescence imaging was performed using a custom spinning-disk confocal microscope setup consisting of a Nikon TE2000 microscope base, attached to a modified Yokogawa CSU-10 unit (Visitech) and an iXon+ Du888 EMCCD camera (Andor). The microscope was also equipped with a 100x/1.45 NA Plan Apo objective (Nikon), Optospin IV filter wheel (Cairn Research), MS-2000 automated stage with CRISP autofocus (ASI), and temperature-controlled chamber maintained at 25°C (OKOlab). The microscope was controlled using Metamorph software (Molecular Devices).

All drug additions during imaging, except for N-starvation experiments, were performed by two consecutive medium exchanges with 400μl of drug-containing medium, using a 1mL polyethylene transfer pipette (Fisher Scientific, 1346-9118). All treatments were completed between the imaging intervals so that the time-lapse acquisition is not disrupted. Due to the mechanical force exerted during the medium exchanges, re-focusing of the sample between acquisitions was sometimes needed.

For conventional LatA addition experiments, YE5S containing 50 μM LatA and 1% DMSO (all final concentrations) was used. YE5S containing 1% DMSO was used as a negative control. Exchange of medium was performed between frames 4 and 5 of imaging (i.e. 9 -12 min after start of imaging).

For experiments involving 3-BrB-PP1 pre-treatment prior to LatA addition, exchange of medium to YE5S containing 5 μM (or 30 μM) 3-BrB-PP1 was performed 30 s before the start of imaging. Subsequent exchange of medium to YE5S containing 5 μM (or 30 μM) 3BrB-PP1 plus 50 μM LatA was performed between frames 4 and 5 of imaging, as above.

For cycloheximide (CHX) pre-treatments, exchange of medium to YE5S containing 100 μg/mL CHX (from 10 mg/mL CHX stock in water; Sigma; C1988) was performed 30 seconds before start of imaging. Subsequent exchange of medium to YE5S containing 100 μg/mL CHX plus 50 μM LatA was performed between frames 4 and 5 of imaging, as above.

For 3-BrB-PP1 removal experiments in *SISA* cells, cells were first grown and prepared for imaging in YE5S containing 5 μM 3-BrB-PP1. MatTek dishes were used for imaging. 3-BrB-PP1 removal was performed by three separate rounds of medium exchange after frames 3, 4 and 5 (i.e. 6 -15 min after start of imaging). Each round of medium exchange involved 6 x 1mL exchanges to YE5S. These additional exchanges (18 in total, far greater than in drug-addition experiments) were necessary for complete removal of 3-BrB-PP1. For this reason, the stated time of 3-BrB-PP1 removal in these experiments is approximate (we chose frame 5 to represent the “0 min” time). For the 3-BrB-PP1 re-addition experiments, a conventional medium exchange to YE5S containing 5 μM 3-BrB-PP1 was performed between frames 35 and 36 (102 -105 min after start of imaging).

For N-starvation experiments, cells were first grown as pre-cultures overnight in low-N EMM2 at 25°C, and then diluted into fresh low-N EMM2 and grown for another 12-16 hours at 25°C to a density of 1.5-5 x 10^6 cells/mL. To exchange to EMM2-N medium, 1 mL of cells was washed twice by 1 min centrifugation at 4,000 rpm and resuspension in an equal volume of EMM2-N medium. Cells were then placed on a lectin-coated MatTek coverslip dish as described above and covered with 600μL of EMM2-N. Prior to imaging, the cells were incubated on the dish for 10 hours at 25 °C, during which time most cells had divided twice to generate chains of four small cells. For addition of 3-BrB-PP1, the medium from the dish was withdrawn using a 1mL transfer pipette and quickly transferred to a microfuge tube already containing 0.6 μL of 5 mM 3-BrB-PP1 in EMM2-N, thereby converting the existing culture medium to EMM2-N containing 5μM 3-BrB-PP1. This medium was then returned to the cell dish, withdrawn once more and then returned again to the dish; this ensures good mixing without perturbing the cells. To mimic this treatment for control mock-treated cells (i.e. cells not treated with 3-BrB-PP1), medium was withdrawn from the dish, returned to the dish, withdrawn once more, and then returned again. The medium changes were performed between frames 11 and 12 (60-66 min after start of imaging). Medium changes of this nature were used instead of using fresh EMM2-N because fresh, “non-conditioned” EMM2-N appeared to cause a shock to the cells.

For IPTG-GFP induction experiments, cells were initially grown in PMG (with appropriate supplements), and exchange of medium was performed as described above for drug addition. PMG was exchanged either to: fresh PMG; or to fresh PMG containing 5 mM IPTG (from 1M stock in water); or to fresh PMG containing 5 mM IPTG plus 100 μg/mL CHX. Exchange of medium was performed 30 s before the start of imaging. In an additional experiment, to assay inhibition of protein synthesis after GFP expression was already induced, medium was exchanged from PMG to PMG containing 5 mM IPTG at 30 s before the start of imaging, and then later from PMG containing 5 mM IPTG to PMG containing 5mM IPTG plus 100 μg/mL CHX, between frames 36 - 37 (175 -180 min after start of imaging).

For DIC images of *SISA* cells after 3-BrB-PP1 removal, 1mL of exponentially growing cells in YE5S containing 5μM 3-BrB-PP1 was washed twice by 1 min centrifugation at 4,000 rpm and resuspension in an equal volume of YE5S. Washed cells were then further diluted with an additional 4 mL of YE5S and incubated at 25° for 24 hours, prior to imaging. Images were taken using a

DeltaVision Elite system using Olympus 60x / 1.42 Oil Plan APO objective. Control cells grown in YE5S containing 5μM 3-BrB-PP1 were imaged during exponential growth.

For phalloidin staining of fixed cells after LatA treatment, cells *not* expressing Lifeact-mCherry were grown to exponential phase in YE5S, and LatA was added to a final concentration of 50 μM LatA. Fixation with formaldehyde and staining with Alexa Fluor 594 phalloidin (Molecular Probes) were exactly as described previously [S1]. The zero time point represents cells just prior to LatA addition.

For phalloidin staining of Sty1-activated *SISA* cells, *SISA* cells (*not* expressing Lifeact-mCherry) were grown in YE5S containing 5μM 3-BrB-PP1, and then washed twice by filtration and resuspension in YE5S medium lacking 5μM 3-BrB-PP1, as described above for physiological experiments. Fixation and staining procedures were exactly as described previously [S1]. The zero time point represents cells just prior to filtration.

Image acquisition parameters:

Image Type	Laser power (%)*, exposure time (ms)			Z-series	Time-lapse
	488 nm	514 nm	594 nm		
CRIB-3xmCitrine		2%, 40 ms		11x 0.6 μm	60 x 3 min
Lifeact-mCherry			30%, 100 ms	11x 0.6 μm	60 x 3 min
CRIB-3xmCitrine (N-starvation)		2%, 100 ms		11x 0.6 μm	40 x 6 min
GFP-IPTG	5%, 150 ms			11x 0.9 μm	72 x 5 min
Sty1-mECitrine		2%, 200 ms		9x 0.7 μm	30 x 5 min
Cdc42-mCherry^{SW}			30%, 100ms	11x 0.6 μm	60 x 3 min
CRIB-3xmCitrine (with Scd2-3xmCherry)		2%, 40 ms		5x 1 μm	60 x 3 min
Scd2-3xmCherry			80%, 300ms	5x 1 μm	6 x 30 min
Alexa Fluor 594 phalloidin (fixed cells)			60%, 200ms	11x 0.6 μm	N/A
CRIB-3xmCitrine (fixed cells)		10%, 100ms		11x 0.6 μm	N/A

* "100%" power = 50 mW for 488 nm laser, 40 mW for 514 nm laser, and 50mW for 594 nm laser.

Processing of the acquired raw images was done using ImageJ (NIH), using the StackReg plugin for Rigid Body registration (http://bigwww.epfl.ch/publications/thevenaz9801.html) and KymoResliceWide plugin for kymographs (http://fiji.sc/KymoResliceWide). Images were adjusted using linear contrast enhancement and are presented as maximum or sum projections containing the entire cell volume. In one image sequence (corresponding to the wild-type cells shown in Figure 4B, and the same cells in Movie S4), a small manual linear contrast adjustment was necessary for three frames of the image sequence, due to a brief change in ambient light conditions; this did not affect the outcome of the experiment or its interpretation. Formatting of images for publication was done using Illustrator CS3 (Adobe). Movies were assembled using ImageJ and QuickTime (Apple).

Quantification of Sty1-mECitrine levels in the nucleus was obtained from movies by automated image analysis. For DMSO treatment, 14 cells were analyzed. For LatA treatment, 27 cells were analyzed. First, segmentation algorithms were used to identify cells and nuclei from maximum projections of z-stacks. Cell segmentation was carried out by the active contour Chan-Vese method, implemented in MATLAB (R2013a; Mathworks). Initial binary masks for cell segmentation were obtained using multi-threshold Otsu's method (*multithresh* in MATLAB) with nine thresholds. Bitmaps containing only those pixels that were above the fifth threshold were then processed using additional morphological operations to: 1) first, identify pixel clusters ("connected components"; *bwconncomp* in MATLAB); 2) then, join nearby clusters by removing holes between neighboring clusters; and 3) finally, remove small pixel clusters (= out of focus cells) and pixel clusters touching frame borders (= partial, incomplete cells). Nucleus segmentation was carried out starting with the masks obtained by cell segmentation. First, pixel intensity values in each individual cell were separately normalized to an interval from 0 to 1. Then, multi-threshold Otsu's method with five thresholds was used to construct bitmaps containing only those pixels that were above the third threshold. Identification of morphologically connected clusters was then used to generate a mask for the nucleus belonging to a given cell. Cells that contained two nuclei (i.e. dividing cells) were rejected from analysis. From the cell segmentation and nucleus segmentation we obtained binary masks of nucleus and corresponding cytosol (by subtracting nucleus mask from cell mask) for every cell, as well as a binary mask for extracellular space (background). Sum projections of z-stacks were used for

quantification of signals inside these masks. The nucleus-specific fluorescence intensity was calculated as $\left[\frac{(I_n - I_b)}{(I_c - I_b)} - 1\right]$, where I_n, I_b and I_c are average intensities in nucleus, cytosol and background, respectively. We note that in these experiments, a small, transient increase in Sty1-mECitrine nuclear fluorescence was observed in DMSO-treated control cells upon addition of DMSO. This is reproducible and appears to be a consequence of acute DMSO addition, from which the cell rapidly recovers.

For quantification of GFP expression under different conditions in IPTG-induction experiments, movies were acquired at multiple stage positions for each condition. For each condition, the time-course of expression was then determined as follows: First, sum projections of images were made for all time-points of movies, and background extracellular fluorescence was subtracted to calculate the value of the fluorescent signal $S_{c,t}$ for a given cell c (or small group of cells) at a given time-point t. These values were then normalized to the fluorescent signal $S_{c,1}$ for the same cell (or small group of cells) at the first time-point. Then, for each time-point t, the mean value of normalized fluorescence was calculated by averaging over all n cells imaged. We then subtracted 1 from mean values in order to generate a baseline of zero at the first time-point, and we then multiplied this result by an arbitrary constant (100) to generate a final value $100\times\left[\frac{1}{n}\left(\sum_{c=1}^{n}\frac{S_{c,t}}{S_{c,1}}\right) - 1\right]$, which represents induced GFP fluorescence (in arbitrary units) for each time-point t. For each of the different conditions, the number of cells (n) imaged was as follows: -IPTG (n =26); +IPTG at t=0, +CHX at t=0 (n =23); +IPTG at t=0, +CHX at t=180 min (n =19); +IPTG (n =16).

Supplemental References

S1. Sawin, K.E., and Nurse, P. (1998). Regulation of cell polarity by microtubules in fission yeast. J Cell Biol *142*, 457-471.

S2. Kelly, F.D., and Nurse, P. (2011). Spatial control of Cdc42 activation determines cell width in fission yeast. Mol Biol Cell *22*, 3801-3811.

S3. Bendezu, F.O., Vincenzetti, V., Vavylonis, D., Wyss, R., Vogel, H., and Martin, S.G. (2015). Spontaneous Cdc42 polarization independent of GDI-mediated extraction and actin-based trafficking. PLoS Biol *13*, e1002097.

S4. Gordon, A., Colman-Lerner, A., Chin, T.E., Benjamin, K.R., Yu, R.C., and Brent, R. (2007). Single-cell quantification of molecules and rates using open-source microscope-based cytometry. Nat Methods *4*, 175-181.

S5. Grallert, A., Patel, A., Tallada, V.A., Chan, K.Y., Bagley, S., Krapp, A., Simanis, V., and Hagan, I.M. (2013). Centrosomal MPF triggers the mitotic and morphogenetic switches of fission yeast. Nat Cell Biol *15*, 88-95.

S6. Ohkura, H., Hagan, I.M., and Glover, D.M. (1995). The conserved Schizosaccharomyces pombe kinase plo1, required to form a bipolar spindle, the actin ring, and septum, can drive septum formation in G1 and G2 cells. Genes Dev *9*, 1059-1073.

S7. Petersen, J., and Hagan, I.M. (2005). Polo kinase links the stress pathway to cell cycle control and tip growth in fission yeast. Nature *435*, 507-512.

S8. Forsburg, S.L., and Rhind, N. (2006). Basic methods for fission yeast. Yeast *23*, 173-183.

S9. Bahler, J., Wu, J.Q., Longtine, M.S., Shah, N.G., McKenzie, A., 3rd, Steever, A.B., Wach, A., Philippsen, P., and Pringle, J.R. (1998). Heterologous modules for efficient and versatile PCR-based gene targeting in Schizosaccharomyces pombe. Yeast *14*, 943-951.

S10. Jaquenoud, M., and Peter, M. (2000). Gic2p may link activated Cdc42p to components involved in actin polarization, including Bni1p and Bud6p (Aip3p). Mol Cell Biol *20*, 6244-6258.

S11. Okada, S., Leda, M., Hanna, J., Savage, N.S., Bi, E., and Goryachev, A.B. (2013). Daughter cell identity emerges from the interplay of Cdc42, septins, and exocytosis. Dev Cell *26*, 148-161.

S12. Sakuno, T., Tada, K., and Watanabe, Y. (2009). Kinetochore geometry defined by cohesion within the centromere. Nature *458*, 852-858.

S13. Tatebe, H., Nakano, K., Maximo, R., and Shiozaki, K. (2008). Pom1 DYRK regulates localization of the Rga4 GAP to ensure bipolar activation of Cdc42 in fission yeast. Curr Biol *18*, 322-330.

S14. Kjaerulff, S., and Nielsen, O. (2015). An IPTG-inducible derivative of the fission yeast nmt promoter. Yeast *32*, 469-478.
