# Supplementary figures and images for: Remodeling of the Fission Yeast Cdc42 Cell-Polarity Module via the Sty1 p38 Stress-Activated Protein Kinase Pathway
Source: Curr Biol. 2016 Nov 7;26(21):2921–8. doi: 10.1016/j.cub.2016.08.048 (PMC5106388; doi:10.1016/j.cub.2016.08.048)

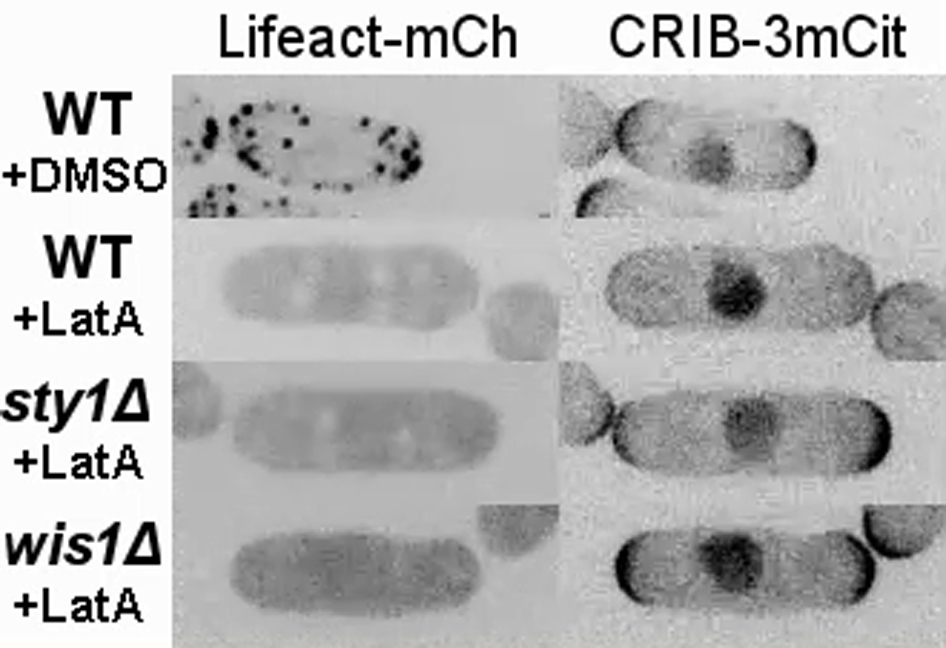

Supplement: Movie S1. CRIB Dispersal after Latrunculin A-Induced Actin Depolymerization Requires the Sty1 MAP Kinase Pathway — Lifeact-mCherry and CRIB-3xmCitrine in wild-type cells after addition of DMSO, and in wild-type, sty1Δ, and wis1Δ cells after addition of 50 μM latrunculin A (LatA). Cells correspond to those shown in Figures 1A and 2A. In wild-type cells, actin depolymerization by LatA leads to CRIB dispersal from cell tips and formation of ectopic CRIB patches on cell sides. In sty1Δ and wis1Δ cells, CRIB remains at cell tips in spite of actin depolymerization, and sty1Δ and wis1Δ cells continue to elongate. Images are at 3-min intervals; total elapsed time is 180 min; time compression is 4,500×. [file mmc2.jpg]

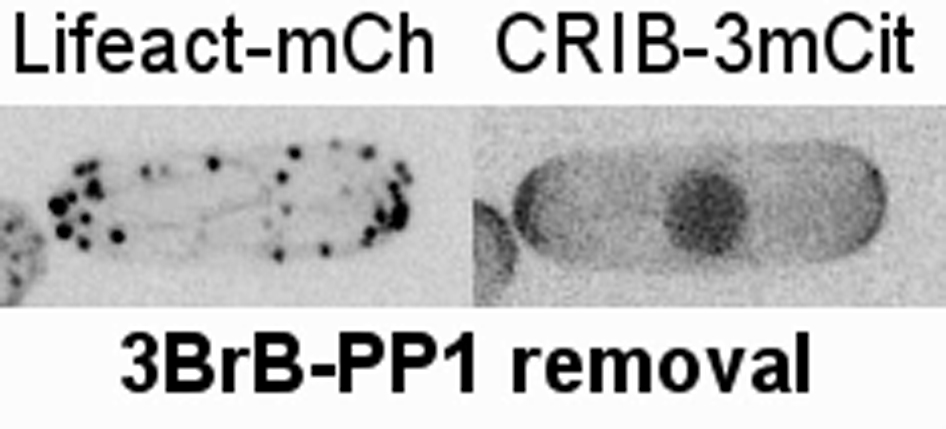

Supplement: Movie S2. Stress-Independent Activation of Sty1 Leads to CRIB Dispersal and Cell Depolarization — Lifeact-mCherry and CRIB-3xmCitrine in SISA cells after 3-BrB-PP1 removal, which leads to Sty1 activation in the absence of external stress. The cell corresponds to that shown in Figure 3E. The timing of 3-BrB-PP1 removal is as indicated; the first few frames shown are prior to removal. Upon 3-BrB-PP1 removal, CRIB disperses from cell tips and forms ectopic patches on cell sides, actin becomes depolarized, but not depolymerized, and cell elongation ceases. Images are at 3-min intervals; total elapsed time is 180 min; time compression is 4,500×. [file mmc3.jpg]

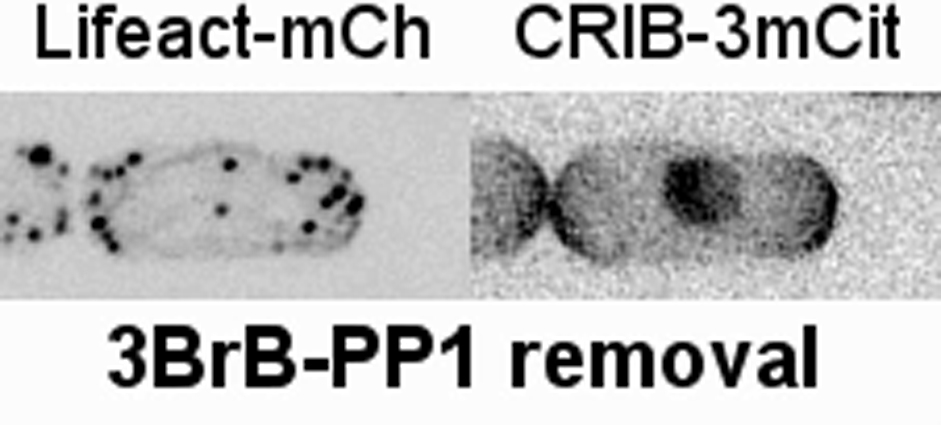

Supplement: Movie S3. Rapid Restoration of Cell Polarity by Inhibition of Sty1 in Sty1-Activated SISA Cells — Lifeact-mCherry and CRIB-3xmCitrine in SISA cells after 3-BrB-PP1 removal, followed by 3-BrB-PP1 re-addition 90 min later. The cell corresponds to that shown in Figure 3F. The timing of 3-BrB-PP1 removal and re-addition is as indicated; the first few frames shown are prior to removal. Just before 3-BrBPP1 re-addition, the movie pauses to show the state of CRIB and actin prior to re-addition. As in Movie S2, 3-BrB-PP1 removal leads to dispersal of CRIB from cell tips, ectopic CRIB patch formation, depolarized actin cytoskeleton, and cessation of cell elongation. 3-BrB-PP1 re-addition quickly reverses all of these effects, with the return of CRIB to cell tips preceding repolarization of the actin cytoskeleton. Images are at 3-min intervals; total elapsed time is 180 min; time compression is 4,500×. [file mmc4.jpg]

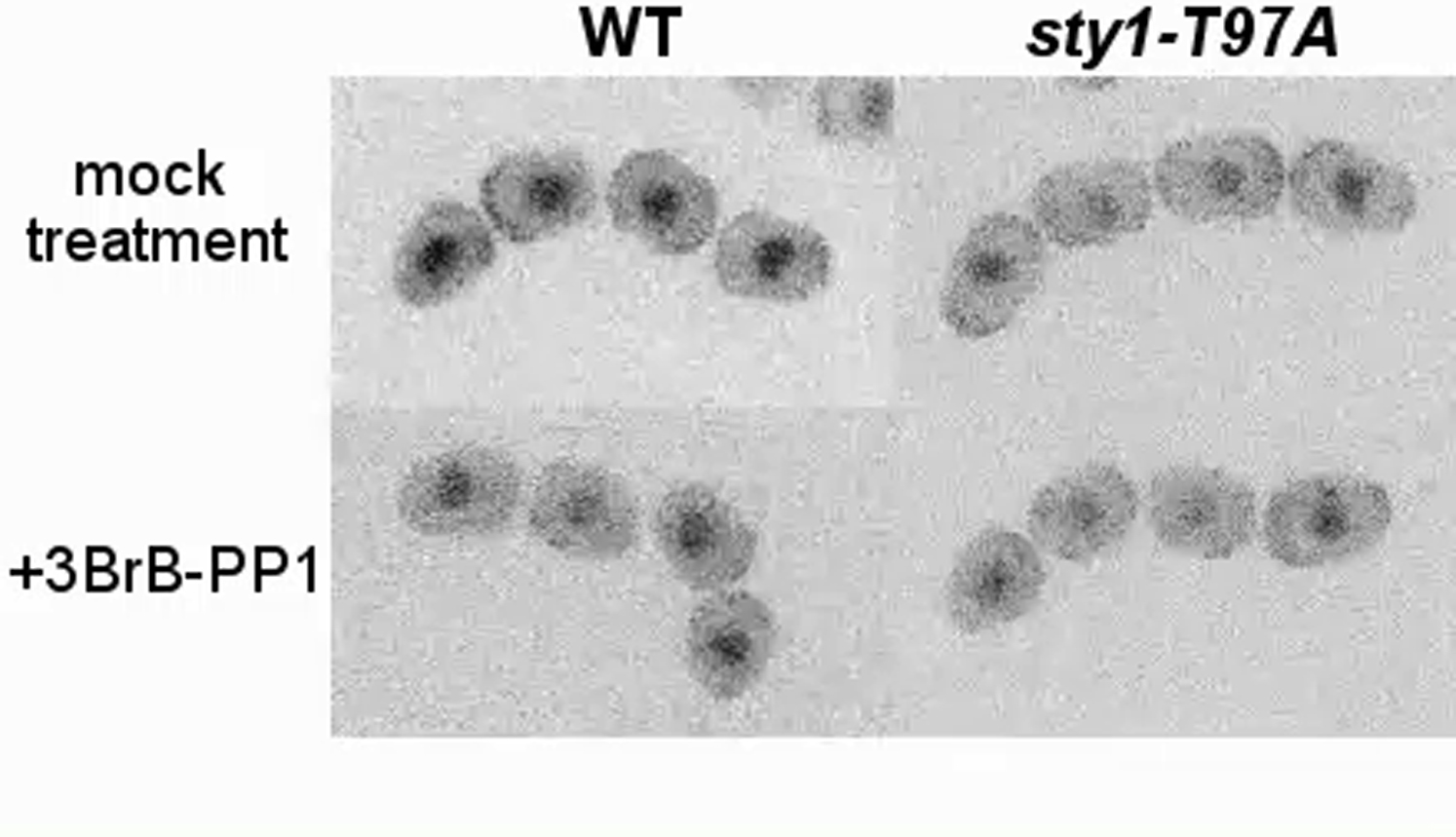

Supplement: Movie S4. Inhibition of Sty1 Causes Repolarization of Depolarized, Quiescent Cells — CRIB-3xmCitrine in nitrogen-starved wild-type and sty1-T97A cells in the absence of 3-BrB-PP1 and after addition of 3-BrB-PP1. Chains of four granddaughter cells are shown. The timing of the addition of 3-BrB-PP1 (or mock treatment) is as indicated. At this time, cells have already been nitrogen starved for 11 hr. 3-BrB-PP1 addition leads to repolarization of CRIB to cell tips and cell elongation in sty1-T97A cells, but not in wild-type cells. Mock treatment maintains the depolarized state in both. Images are at 6-min intervals; total elapsed time is 240 min; time compression is 9,000×. [file mmc5.jpg]
