# Supplementary material for: Remodeling of the Fission Yeast Cdc42 Cell-Polarity Module via the Sty1 p38 Stress-Activated Protein Kinase Pathway
Source: Curr Biol. 2016 Nov 7;26(21):2921–8. doi: 10.1016/j.cub.2016.08.048 (PMC5106388; doi:10.1016/j.cub.2016.08.048)
Supplement: Document S2. Article plus Supplemental Information [file mmc6.pdf]

Current Biology

Report

Remodeling of the Fission Yeast Cdc42 Cell-Polarity Module via the Sty1 p38 Stress-Activated Protein Kinase Pathway

Highlights

- LatA depolymerizes actin, disperses cell-polarity proteins, and activates MAPK Sty1
- There is no cell-polarity dispersal after LatA treatment in *sty1* and *wis1* mutants
- Sty1 activation alone is sufficient for cell-polarity dispersal
- Sty1 inactivation repolarizes quiescent cells

Authors

Delyan R. Mutavchiev, Marcin Leda, Kenneth E. Sawin

Correspondence

ken.sawin@ed.ac.uk

In Brief

Mutavchiev et al. uncover a link between stress signaling and Cdc42-dependent cell polarity in fission yeast. They show that dispersal of the Cdc42 polarity module from growth sites after treatment with the actin drug latrunculin A depends on Sty1, a homolog of mammalian p38 MAP kinase. Activation of Sty1 alone is also sufficient for dispersal.

CrossMark

Mutavchiev et al., 2016, Current Biology *26*, 2921–2928
November 7, 2016 © 2016 The Authors. Published by Elsevier Ltd.
http://dx.doi.org/10.1016/j.cub.2016.08.048

CellPress

Current Biology
Report

CellPress

Remodeling of the Fission Yeast Cdc42 Cell-Polarity Module via the Sty1 p38 Stress-Activated Protein Kinase Pathway

Delyan R. Mutavchiev,[1] Marcin Leda,[2] and Kenneth E. Sawin[1,3,*]
[1]Wellcome Trust Centre for Cell Biology, School of Biological Sciences, University of Edinburgh, Michael Swann Building, Max Born Crescent, Edinburgh EH9 3BF, UK
[2]SynthSys (Centre for Synthetic and Systems Biology), School of Biological Sciences, University of Edinburgh, C.H. Waddington Building, Max Born Crescent, Edinburgh EH9 3BF, UK
[3]Lead Contact
*Correspondence: ken.sawin@ed.ac.uk
http://dx.doi.org/10.1016/j.cub.2016.08.048

SUMMARY

The Rho family GTPase Cdc42 is a key regulator of eukaryotic cellular organization and cell polarity [1]. In the fission yeast *Schizosaccharomyces pombe*, active Cdc42 and associated effectors and regulators (the "Cdc42 polarity module") coordinate polarized growth at cell tips by controlling the actin cytoskeleton and exocytosis [2–4]. Localization of the Cdc42 polarity module to cell tips is thus critical for its function. Here we show that the fission yeast stress-activated protein kinase Sty1, a homolog of mammalian p38 MAP kinase, regulates localization of the Cdc42 polarity module. In wild-type cells, treatment with latrunculin A, a drug that leads to actin depolymerization, induces dispersal of the Cdc42 module from cell tips and cessation of polarized growth [5, 6]. We show that latrunculin A treatment also activates the Sty1 MAP kinase pathway and, strikingly, we find that loss of Sty1 MAP kinase signaling prevents latrunculin A-induced dispersal of the Cdc42 module, allowing polarized growth even in complete absence of the actin cytoskeleton. Regulation of the Cdc42 module by Sty1 is independent of Sty1's role in stress-induced gene expression. We also describe a system for activation of Sty1 kinase "on demand" in the absence of any external stress, and use this to show that Sty1 activation alone is sufficient to disperse the Cdc42 module from cell tips in otherwise unperturbed cells. During nitrogen-starvation-induced quiescence, inhibition of Sty1 converts non-growing, depolarized cells into growing, polarized cells. Our results place MAP kinase Sty1 as an important physiological regulator of the Cdc42 polarity module.

RESULTS AND DISCUSSION

Fluorescent-protein fusions with CRIB (Cdc42/Rac interactive binding motif)-containing domains of Cdc42 effectors are widely used as reporters of active (GTP-bound) Cdc42 localization in vivo in both budding and fission yeasts [7–9]. In fission yeast, after treatment with the actin monomer-binding drug latrunculin A (LatA), which leads to acute depolymerization of the actin cytoskeleton, CRIB-3xGFP disperses from cell tips and forms transient ectopic patches on cell sides, as does Cdc42 itself and other components of the Cdc42 polarity module [5, 6, 10]. Because actin filaments can play a role, for example, in vectorial transport of endomembrane-associated Cdc42 or its regulators to cell tips or in endocytic recycling of Cdc42 from the plasma membrane [4, 11–13], the most straightforward interpretation of this result has been that dispersal of the Cdc42 polarity module from cell tips is a direct consequence of the loss of actin filaments [5, 6].

However, several years ago, in the context of a putative "spindle orientation checkpoint" in fission yeast [14], it was briefly reported that treatment with latrunculin B, a compound related to LatA, leads to activation of the conserved mitogen-activated protein (MAP) kinase Sty1 (also known as Spc1/Phh1 [15–17]). Sty1 is the fission yeast homolog of budding yeast MAP kinase Hog1 and of mammalian stress-activated protein kinase (SAPK) p38 [18–20]. Although there is now considerable evidence against the concept of a spindle orientation checkpoint [21–23], the initial observation of Sty1 activation after latrunculin B treatment was never investigated further. We therefore decided to revisit the question of how the Cdc42 polarity module disperses from cell tips after LatA treatment, and whether Sty1 plays a role in this behavior.

Latrunculin A Treatment Leads Both to CRIB Dispersal and to Sty1 MAP Kinase Activation

We constructed a CRIB-3xmCitrine probe (referred to here simply as CRIB) for long-term time-lapse fluorescence imaging of the Cdc42 polarity module (see the Supplemental Experimental Procedures). Using this together with the F-actin reporter Lifeact-mCherry (here referred to as Lifeact) [24, 25], we confirmed that LatA treatment leads both to actin depolymerization and to CRIB dispersal from cell tips and ectopic patch formation on cell sides (Figures 1A and S1A; Movie S1) [5, 6]. In parallel experiments, the Cdc42 module scaffold protein Scd2 (Scd2-3xmCherry) also dispersed from cell tips after LatA treatment and colocalized with ectopic CRIB patches (Figure S1B)

This is an open access article under the CC BY license (http://creativecommons.org/licenses/by/4.0/).

CellPress

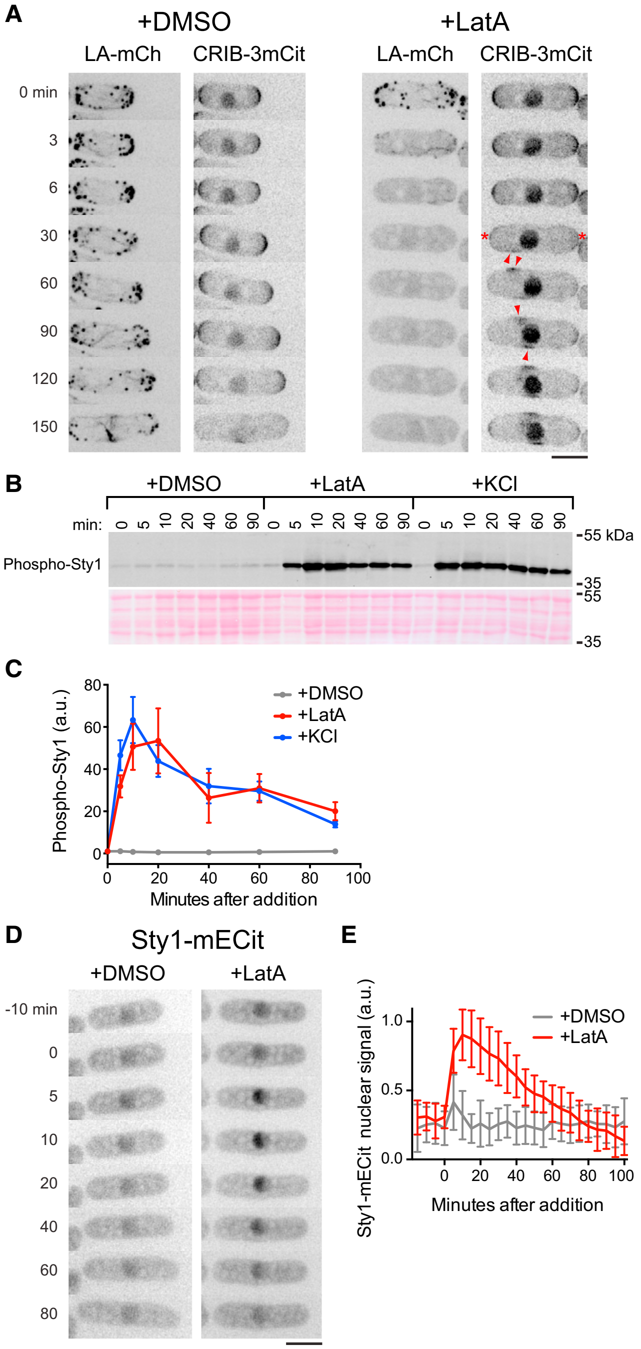

Figure 1. Latrunculin A Treatment Causes Both CRIB Dispersal from Cell Tips and Sty1 MAP Kinase Activation

(A) Still images from movies of Lifeact-mCherry (LA-mCh) and CRIB-3xmCitrine (CRIB-3mCit) in wild-type cells after addition of DMSO or 50 μM latrunculin A (LatA). Asterisks indicate dispersal of CRIB from cell tips. Arrowheads indicate examples of ectopic CRIB patches after dispersal. Nuclear CRIB signal is unrelated to Cdc42 (see the Supplemental Experimental Procedures).

(B) Anti-phospho-Sty1 western blot of wild-type cell extracts after addition of DMSO, 50 μM LatA, or 0.6 M KCl for the indicated times. Ponceau S stain of the same region of the blot is shown below.

(C) Quantification of phospho-Sty1 (a.u.) from experiments of the type shown in (B). Mean values are from three independent experiments. Error bars indicate SEM.

(D) Still images from movies of Sty1-mECitrine in wild-type cells after addition of DMSO or LatA. LatA addition leads to net import of Sty1-mECitrine into the nucleus.

(E) Quantification of Sty1-mECitrine nuclear fluorescence after addition of DMSO or LatA. Values shown are mean relative intensity (a.u.) of nuclear fluorescence above cytoplasmic fluorescence. Error bars indicate SD.

All times shown are relative to addition of DMSO, LatA, or KCl. Scale bars, 5 μm. See also Figure S1 and Movie S1.

[6, 10]. CRIB dispersal from cell tips was considerably slower than actin depolymerization itself, suggesting that dispersal may not be a direct effect of actin depolymerization. In addition, rather than appearing as spontaneous random patches, ectopic CRIB patches generally moved in a concerted fashion away from cell tips, toward the cell center.

In parallel with these experiments, we demonstrated in two independent assays that LatA treatment leads to activation of Sty1 (Figures 1B–1E). As a component of the fission yeast SAPK pathway, Sty1 is normally activated through phosphorylation of Thr171 and Tyr173 by dual-specificity MAP kinase kinase (MAPKK) Wis1 after a variety of stresses, including hyperosmotic and oxidative stress [26, 27]. Western blotting revealed a strong increase in Sty1 phosphorylation after LatA treatment, comparable in amplitude and duration to that observed after a conventional hyperosmotic salt stress (Figures 1B and 1C). Phosphorylation of Sty1 by Wis1 leads to increased Sty1 in the nucleus, where Sty1 functions to promote stress-activated gene expression (although some Sty1 remains cytoplasmic) [26, 28]. We found that after LatA addition, Sty1-mECitrine accumulated in the nucleus with similar kinetics to those observed for Sty1 phosphorylation (Figures 1D and 1E). We conclude that LatA treatment leads to Sty1 activation at a level comparable to that seen after hyperosmotic salt stress.

Sty1 Activity Is Necessary for CRIB Dispersal after LatA Treatment

We next asked whether Sty1 plays a role in LatA-induced CRIB dispersal. We imaged CRIB and Lifeact in *sty1Δ* cells and *wis1Δ* cells treated with LatA. Remarkably, in these cells, CRIB remained at cell tips for the duration of imaging (several hours), despite rapid and complete actin depolymerization (Figures 2A and S1A; Movie S1). Scd2 also remained at cell tips (Figure S1B). Moreover, cell elongation continued after actin depolymerization, unlike wild-type cells, in which elongation ceased immediately (Figures 2A and 2B; Movie S1). These results lead to several important conclusions. First, they demonstrate that the SAPK pathway is required for CRIB dispersal after LatA treatment. Second, and in contrast to interpretations of previous experiments [5, 6], they show that the actin cytoskeleton per se is not required for stability of the Cdc42 polarity module at cell tips. Finally, they show that cell elongation can occur in the complete absence of the actin cytoskeleton. Kymograph analysis revealed that cell elongation in LatA-treated *sty1Δ* and *wis1Δ* cells gradually declines over time (Figure 2B). This could be explained as follows: (1) in the initial period after LatA treatment, tip-localized active Cdc42 can drive cell elongation through positive regulation of exocytosis [29]; (2) however, after LatA treatment, membrane proteins involved in exocytosis would no longer be recycled by endocytic retrieval from the plasma membrane, because

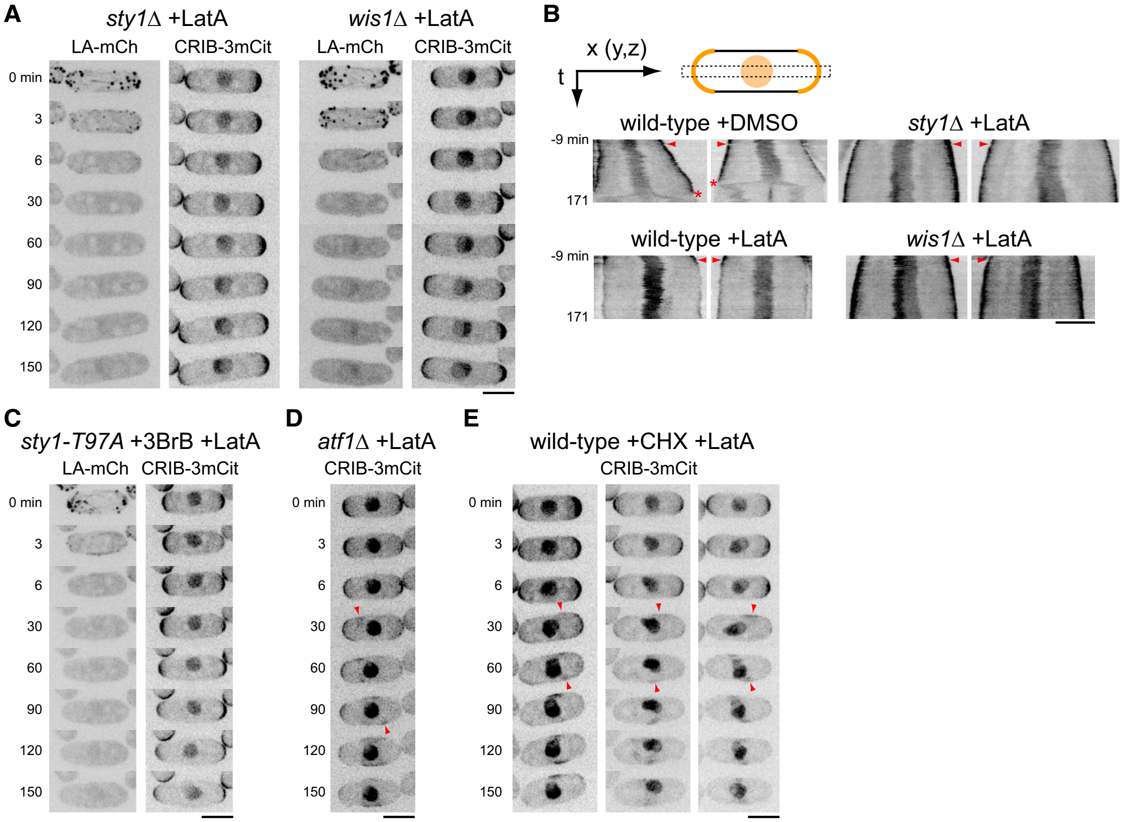

Figure 2. CRIB Dispersal by Latrunculin A Requires the Sty1 MAPK Pathway but Not Sty1-Dependent Gene Expression

(A) Still images from movies of Lifeact-mCherry (LA-mCh) and CRIB-3xmCitrine (CRIB-3mCit) in *sty1Δ* and *wis1Δ* cells after addition of 50 μM latrunculin A (LatA). Although LatA depolymerizes the actin cytoskeleton, CRIB does not disperse, and cells continue to elongate.

(B) Kymographs from movies of CRIB-3mCit showing rates of cell elongation in the indicated strains after addition of DMSO or LatA. The cartoon summarizes kymograph construction (see also Supplemental Experimental Procedures). The orange curves and circle represent CRIB-3mCit fluorescence at cell tips and in nucleus, respectively. The dashed box represents the region used for kymograph scans along the x-axis. Images used for kymograph analysis are z-projections, and kymograph scans measured average intensity values along a line that is five pixels wide on the y-axis (line width corresponds to height of dashed box); therefore, information from y- and z-dimensions is implicit in kymographs. Arrows indicate orientation of x and time (t) axes in kymographs. Left-hand panels in each pair of kymographs represent cells shown in Figures 1A and 2A and Movie S1. Right-hand panels show additional cells. Arrowheads indicate time of DMSO or LatA addition. Asterisks indicate disappearance of CRIB from cell tips in DMSO-treated cells due to cell division.

(C) Still images from movies of LA-mCh and CRIB-3mCit in *sty1-T97A* cells pre-treated with 5 μM 3-BrB-PP1 (3BrB) for 10 min prior to addition of 50 μM LatA in the continued presence of 3BrB.

(D) Still images from movies of CRIB-3mCit in *atf1Δ* cells after addition of 50 μM LatA. Arrowheads indicate examples of ectopic CRIB patches.

(E) Still images from movies of CRIB-3mCit in wild-type cells pre-treated with 100 μg/mL cycloheximide (CHX) for 10 min prior to addition of 50 μM LatA in the continued presence of CHX. Arrowheads indicate examples of ectopic CRIB patches. CHX treatment causes contortions of nuclei to varying extents, and therefore multiple example cells are shown.

All times shown are relative to addition of DMSO or LatA. Scale bars, 5 μm. See also Figures S1 and S2 and Movie S1.

endocytosis in yeasts depends on the actin cytoskeleton [30]; and therefore (3) such proteins will eventually be depleted from cytoplasmic pools, ultimately leading to cessation of elongation.

Our results suggest a model in which activation of Sty1 by LatA treatment leads to dispersal of the Cdc42 polarity module from cell tips. An alternative view, at least in principle, could be that because Sty1 contributes to multiple cellular pathways [26], *sty1* deletion might lead to a long-term physiological adaptation that fundamentally alters behavior of the Cdc42 module, even prior to any stress (according to this view, LatA-induced activation of Sty1 would be purely coincidental). To rule out this possibility, we imaged CRIB and Lifeact in *sty1-T97A* cells, in which mutation of Thr97 within Sty1's ATP-binding pocket allows kinase activity to be specifically inhibited by ATP-competitive analogs [31, 32] (*T97A* is the equivalent of an "*as2*" mutation). We treated *sty1-T97A* cells with the analog 3-BrB-PP1 (4-Amino-1-tert-butyl-3-(3-bromobenzyl)pyrazolo[3,4-d]pyrimidine) for less than 10 min, so that no long-term adaptation could occur, and then added LatA in the continued presence of 3-BrB-PP1. In these cells, LatA addition led to actin depolymerization but CRIB remained at cell tips, just as in *sty1Δ* and *wis1Δ* cells, and cells also continued to elongate (Figures 2C and S2A). Collectively, these results demonstrate that LatA-induced CRIB dispersal is not a passive process (e.g., a simple consequence of actin depolymerization) but rather an active process that depends on the SAPK pathway and Sty1 kinase activity.

To our knowledge, this is the first indication of such regulation of the Cdc42 polarity module by a MAP kinase pathway.

The best-studied role of Sty1 in response to stress is in the regulation of gene expression, and a key Sty1 substrate is the conserved basic leucine zipper domain (bZIP) transcription factor Atf1 [26, 28, 33–35]. We found that LatA treatment in *atf1Δ* cells still led to CRIB dispersal (Figure 2D), suggesting that Sty1-dependent changes in gene expression are unlikely to be required for CRIB dispersal. To strengthen these findings, we pre-treated wild-type cells with cycloheximide to inhibit all protein synthesis prior to LatA addition and imaging. In these cells, LatA treatment still led to CRIB dispersal (Figures 2E and S2B). We conclude that the role of Sty1 in promoting CRIB dispersal is independent of stress-induced gene expression.

Polo kinase Plo1, a downstream target of the Sty1 SAPK pathway (phosphorylated on Ser402 after some, but not all, types of stress [36]), has been implicated in regulation of cell polarity [36, 37]. We used 3-BrB-PP1 together with analog-sensitive *plo1-as8* cells [37] as well as *plo1-402A* and *plo1-S402E* mutants [36] to test whether Plo1 is involved in LatA-induced CRIB dispersal. In all cases, LatA treatment led to CRIB dispersal (Figures S1C and S1D), suggesting that Plo1 is not a critical Sty1 target for CRIB dispersal.

Sty1 Activation Is Sufficient for CRIB Dispersal in the Absence of External Stress

Thus far, our results show that Sty1 is activated by LatA treatment and that Sty1 activity is necessary for LatA-induced CRIB dispersal from cell tips. We next asked whether Sty1 activation alone (without LatA treatment) is sufficient to drive CRIB dispersal.

To test this, we developed a system to rapidly switch on Sty1 activity in vivo in the absence of any external stress. We combined a constitutively active MAPKK allele (*wis1-DD* [38]) with the analog-sensitive *sty1-T97A* allele, together with deletion of two genes encoding tyrosine phosphatases, *pyp1+* and *pyp2+*, which normally attenuate Sty1 MAPK signaling via dephosphorylation of Sty1 Tyr173 [15, 16] (see the Supplemental Experimental Procedures). For simplicity, we will refer to this combination of mutations as *SISA* (stress-independent Sty1 activation). We reasoned that in *SISA* cells grown in the presence of 3-BrB-PP1, Sty1 will be "poised" to be active (because it is phosphorylated by constitutively active Wis1-DD) but nevertheless inhibited by 3-BrB-PP1. Accordingly, upon removal of 3-BrB-PP1, Sty1 activity should rapidly increase.

SISA cells displayed normal growth and morphology when grown in 5 μM 3-BrB-PP1 but stopped dividing upon removal of 3-BrB-PP1, eventually becoming large and swollen in the middle (Figures 3A and 3B). *SISA* cells were also unable to form colonies on solid media lacking 3-BrB-PP1. To confirm that these phenotypes were associated with increased Sty1 activity, we assayed Atf1 in *SISA* cells by western blotting. Removal of 3-BrB-PP1 led to a small but reproducible shift in Atf1 migration

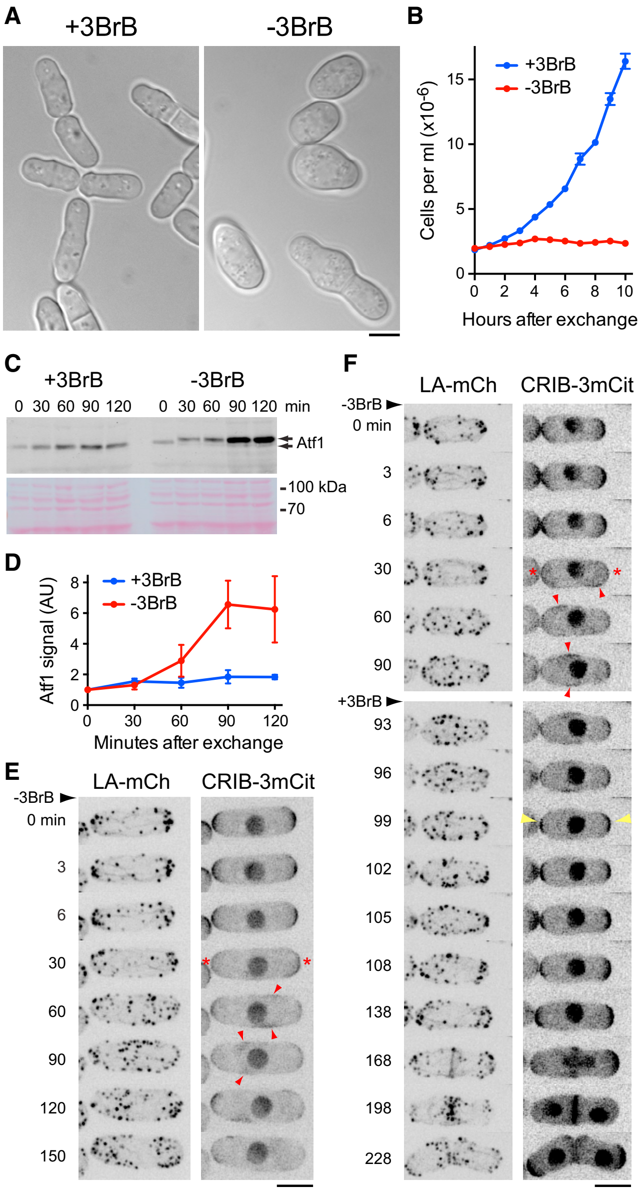

Figure 3. Sty1 Activation Drives CRIB Dispersal in the Absence of Any External Stress

(A) DIC images of *SISA* (*wis1-DD sty1-T97A pyp1Δ pyp2Δ*) cells grown in the presence of 3-BrB-PP1 (+3BrB) and 24 hr after 3-BrB-PP1 removal (−3BrB).

(B) Cell-number growth curves of *SISA* cells in the presence of 3-BrB-PP1 and after 3-BrB-PP1 removal. Error bars indicate SEM.

(C) Anti-Atf1 western blot of extracts from *SISA* cells in the presence of 3-BrB-PP1 (+3BrB) and after removal (−3BrB). Times are relative to initiation of 3-BrB-PP1 removal. After removal, there is a discrete migration shift of Atf1 from 30 min onward, as well as increased levels of Atf1, especially at 90 and 120 min. Ponceau S stain of the same region of the blot is shown below.

(D) Quantification of Atf1 levels in *SISA* cells from experiments of the type shown in (C). Mean values are from three independent experiments. Error bars indicate SEM.

(E) Still images from movies of Lifeact-mCherry (LA-mCh) and CRIB-3xmCitrine (CRIB-3mCit) in *SISA* cells after 3-BrB-PP1 removal. Asterisks indicate CRIB dispersal from cell tips. Arrowheads indicate ectopic CRIB patches. Times are relative to initiation of removal.

(F) Still images from movies of LA-mCh and CRIB-3mCit in *SISA* cells after 3-BrB-PP1 removal and subsequent 3-BrB-PP1 re-addition 90 min later. Asterisks indicate CRIB dispersal from cell tips. Red arrowheads indicate ectopic CRIB patches. Yellow arrowheads indicate recovery of CRIB to cell tips upon 3-BrB-PP1 re-addition. Times are relative to initiation of removal.

Scale bars, 5 μm. See also Figure S3 and Movie S2.

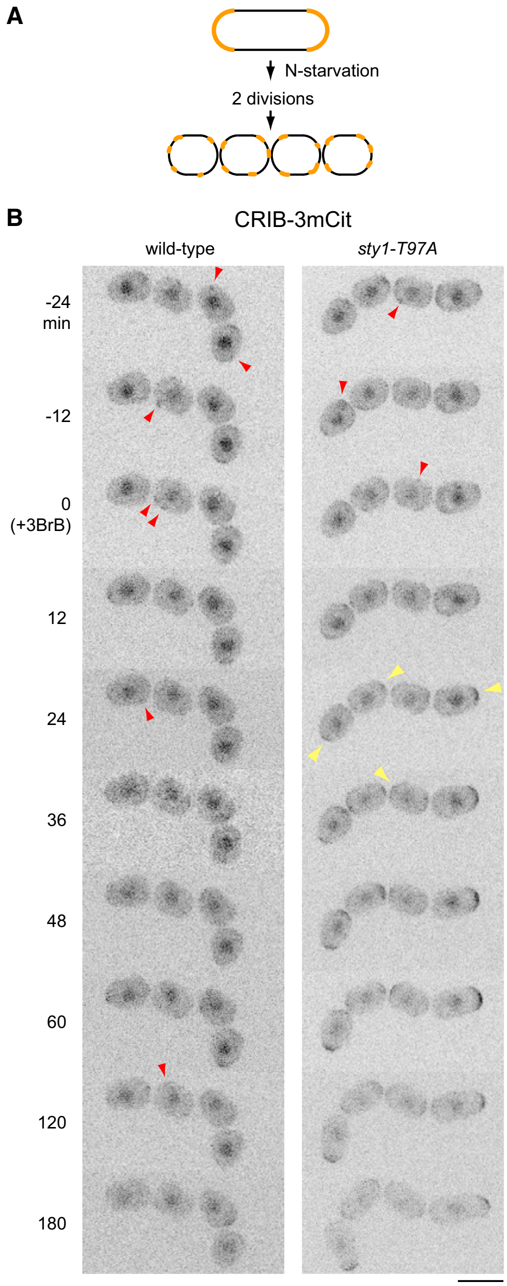

Figure 4. Inhibition of Sty1 Leads to Repolarization of Quiescent Cells

(A) Cartoon of cell divisions after nitrogen starvation (N starvation) in heterothallic cells. Upon N starvation, cells divide twice en route to quiescence, with relatively little elongation during interphase. This produces a chain of four small granddaughter cells that lack any overall polarization. Cell-polarity machinery (e.g., CRIB-3xmCitrine) is shown in orange.

(B) Still images from movies of CRIB-3xmCitrine (CRIB-3mCit) in N-starved wild-type and *sty1-T97A* cells before and after addition of 3-BrB-PP1 (3BrB). Chains of four granddaughter cells are shown. Red arrowheads indicate ectopic patches of CRIB-3mCit. Yellow arrowheads indicate repolarization of CRIB-3mCit. Note the elongation of *sty1-T97A* cells after 3BrB-induced repolarization, and the absence of repolarization/elongation in wild-type cells. Times shown are relative to 3BrB addition; at 0 min, cells have already been N starved for 11 hr, including 1 hr of imaging.

Scale bar, 5 μm. See also Movie S4, in which ectopic CRIB patches are more apparent.

on SDS-PAGE, as well as significantly increased Atf1 levels, matching what occurs after stress-induced Sty1 activation (Figures 3C and 3D) [34, 39, 40].

We next imaged CRIB and Lifeact in *SISA* cells (Figures 3E and 3F; Movie S2). In the presence of 3-BrB-PP1, CRIB and Lifeact distributions were indistinguishable from wild-type cells. However, upon removal of 3-BrB-PP1, CRIB dispersed from cell tips within ~30 min, and ectopic CRIB patches appeared on cell sides, as in LatA-treated wild-type cells. Other components of the Cdc42 polarity module also dispersed from cell tips and formed ectopic patches (Figures S3A and S3B), and concomitantly the actin cytoskeleton became depolarized (but not depolymerized; Figures 3E and S3C). These results indicate that an acute increase in Sty1 activity is sufficient to promote dispersal of the Cdc42 polarity module from cell tips, even in the absence of any external stress. In conjunction with our previous observations, this provides extremely strong evidence that the Cdc42 polarity module is specifically regulated by the Sty1 MAP kinase pathway.

We also used *SISA* cells to investigate how Sty1 inactivation affects CRIB localization in cells containing already-dispersed CRIB. Sty1 was activated by 3-BrB-PP1 removal and then inactivated 90 min later by re-addition of 3-BrB-PP1 (Figure 3F; Movie S3). Strikingly, upon re-addition of 3-BrB-PP1, the dispersed CRIB rapidly returned to cell tips, and this preceded the return of actin to cell tips. This suggests that key polarity landmarks are retained at cell tips after Sty1-activated CRIB dispersal and that, upon Sty1 inactivation, the Cdc42 polarity module can follow these cues independent of the polarization of the actin cytoskeleton.

Inhibition of Sty1 during Quiescence Leads to Cell Repolarization

The experiments described thus far involve activation of Sty1 either via external stress (LatA) or by use of *SISA* cells. We therefore investigated a role for Sty1 in cell polarity in an additional physiological setting, nitrogen starvation (N starvation), which is a prerequisite to mating and meiosis in fission yeast [41, 42]. Upon N starvation, cells normally divide twice in succession without significant interphase cell elongation, leading to the generation of four small cells (Figure 4A). Homothallic (self-mating) cells then mate, whereas heterothallic cells in the absence of a mating partner enter a long-term, quiescent G0-like state [43] in which the Cdc42 polarity module appears to become depolarized [44]. Because *sty1Δ* mutants are partially sterile [17, 34], we hypothesized that Sty1 may be involved in maintaining this depolarized state, which might be important for an exploratory phase of cell polarity when mating partners are present [44].

We imaged CRIB in N-starved heterothallic wild-type and *sty1-T97A* cells before and after addition of 3-BrB-PP1 (Figure 4B; Movie S4). In the absence of 3-BrB-PP1, all cells showed disperse, dynamic patches of CRIB, and these patches remained dynamic, without any cell elongation, for the duration of imaging (4 hr; Movie S4). This dynamic behavior was unchanged by addition of 3-BrB-PP1 to wild-type cells. However, after 3-BrB-PP1 addition to *sty1-T97A* cells, CRIB rapidly localized to cell tips, and cells resumed elongation, even though this is futile, as *sty1Δ* cells rapidly lose viability upon N starvation [15–17].

The recovery of CRIB to cell tips and resumption of cell elongation upon Sty1 inhibition demonstrate that Sty1 activity is critical for maintaining a non-polarized Cdc42 module in N-starved quiescent cells. Although many mutants in the SAPK pathway have defects in mating and meiosis, this result may help to explain why *sty1Δ* and *wis1Δ* mutants in particular continue to elongate upon N starvation, unlike other mutants in the pathway [45, 46].

The Fission Yeast SAPK Pathway in Regulation of Cell Polarity

The fission yeast SAPK pathway is activated by a wide variety of stresses, including hyperosmotic stress, oxidative stress, temperature stress, nutritional stress, heavy metals, and hypergravity [26, 27]. Our demonstration that Sty1 activation is sufficient for CRIB dispersal independent of any external stress is particularly important because each type of SAPK-activating stress is likely to have additional type-specific effects on cell physiology. With the exception of oxidative stress, in essentially all cases the route from initial stress to Sty1 activation is poorly understood [27]. Currently, it is not clear exactly how LatA treatment leads to Sty1 activation. One interesting possibility is that LatA treatment provokes a specific "actin stress response" that signals to the SAPK pathway; this certainly merits further investigation, as it would suggest the possibility of a checkpoint that disrupts cell polarity in response to cytoskeleton depolymerization. Alternatively, the pathway from LatA treatment to Sty1 activation could involve additional, non-actin targets of LatA, independent of actin depolymerization, although this would not affect our conclusions concerning Sty1 regulation of the Cdc42 polarity module. Future work will help to illuminate how LatA activates the SAPK pathway.

The system of SAPK-dependent cell-polarity regulation in fission yeast described here does not appear to have a direct counterpart in budding yeast. Hog1, the budding yeast homolog of Sty1 and mammalian p38, is strongly activated by osmotic stress but only weakly/moderately by several other stresses (and with significantly different kinetics [19]). Consistent with this, LatA treatment does not activate Hog1 [47]. In addition, in spite of extensive genetic and biochemical analysis over many years, there is currently no evidence that the Hog1 pathway regulates the Cdc42 polarity module, mating efficiency, or responses to nutrient deprivation [18, 19]. Treatment of budding yeast with the related compound latrunculin B has been shown to activate a different MAP kinase, Slt2 (also known as Mpk1 [48]), but it is not clear what relation, if any, this system bears to our work. Both the kinetics and other qualitative aspects of Slt2 activation after latrunculin B treatment [48] are markedly different from what we observe for Sty1 after LatA treatment. Moreover, Slt2 is part of the cell-integrity MAPK pathway—a different pathway altogether from the SAPK pathway—and rather than regulating cell polarity, Slt2 promotes G2 cell-cycle arrest. We speculate that in spite of a largely conserved "parts list" for MAPK signaling and cell-polarity regulation in budding and fission yeast, the different modes of polarized growth specific to each yeast [3] have led to different wiring patterns for MAP kinase activation and downstream targets. Further understanding of SAPK-dependent control of cell polarity in fission yeast may help to determine whether similar control exists in metazoan cells. An additional consequence of our results is that, for some cell types, it may be necessary to re-evaluate experiments that use LatA to investigate how the actin cytoskeleton contributes to various cellular functions, because observed effects may be the result of a stress response rather than actin depolymerization per se.

Although the molecular mechanisms by which Sty1 regulates the Cdc42 polarity module remain to be elucidated, two observations in particular provide some insight. First, although Sty1 activation leads to dispersal of CRIB and other components of the Cdc42 module from cell tips, these nevertheless remain largely concentrated in patches on the plasma membrane [5]. This indicates that the Cdc42 module is at least partially intact under these conditions, albeit misplaced and more dynamic. Second, in our experiments in which 3-BrB-PP1 is first removed from *SISA* cells and then later added back, the recovery of CRIB to cell tips is very rapid, occurring within a few minutes. Collectively, these results suggest that Sty1 may act to negatively regulate the coupling of the Cdc42 module to cell-polarity landmarks [3], some of which may remain at cell tips under stress conditions. Further confirmation of this view will rely on the identification of the proteins involved in coupling, together with an understanding of how they are directly or indirectly regulated by Sty1.

EXPERIMENTAL PROCEDURES

Standard fission yeast genetics and imaging techniques were used throughout. Detailed descriptions of strain construction, physiological experiments, and microscopy methods are provided in the Supplemental Experimental Procedures.

SUPPLEMENTAL INFORMATION

Supplemental Information includes Supplemental Experimental Procedures, three figures, and four movies and can be found with this article online at http://dx.doi.org/10.1016/j.cub.2016.08.048.

AUTHOR CONTRIBUTIONS

D.R.M. and K.E.S. designed the experiments, D.R.M. performed the experiments; M.L. created analytical tools and performed the analysis; and D.R.M. and K.E.S. wrote the paper.

ACKNOWLEDGMENTS

We thank M. Balasubramanian, I. Hagan, E. Hidalgo, S. Martin, J. Millar, O. Nielsen, and K. Shiozaki for strains and reagents; E. Bi, A. Kerr, and S. Okada for help in designing CRIB-3xmCitrine; and D. Kelly and S. White for help with microscopy and cell counting. We thank A. Goryachev, H. Ohkura, and members of our laboratory for discussions. This work was supported by grants from the Wellcome Trust (099842 and 094517) and BBRSC (BB/K021699/1). The Wellcome Trust Centre for Cell Biology is supported by core funding from the Wellcome Trust (092076).

Received: May 7, 2016
Revised: July 24, 2016
Accepted: August 19, 2016
Published: October 13, 2016

REFERENCES

1. Etienne-Manneville, S. (2004). Cdc42—the centre of polarity. J. Cell Sci. *117*, 1291–1300.

2. Rincón, S.A., Estravís, M., and Pérez, P. (2014). Cdc42 regulates polarized growth and cell integrity in fission yeast. Biochem. Soc. Trans. *42*, 201–205.

3. Martin, S.G., and Arkowitz, R.A. (2014). Cell polarization in budding and fission yeasts. FEMS Microbiol. Rev. *38*, 228–253.

4. Martin, S.G. (2015). Spontaneous cell polarization: feedback control of Cdc42 GTPase breaks cellular symmetry. Bioessays *37*, 1193–1201.

5. Bendezú, F.O., and Martin, S.G. (2011). Actin cables and the exocyst form two independent morphogenesis pathways in the fission yeast. Mol. Biol. Cell *22*, 44–53.

6. Bendezú, F.O., Vincenzetti, V., Vavylonis, D., Wyss, R., Vogel, H., and Martin, S.G. (2015). Spontaneous Cdc42 polarization independent of GDI-mediated extraction and actin-based trafficking. PLoS Biol. *13*, e1002097.

7. Jaquenoud, M., and Peter, M. (2000). Gic2p may link activated Cdc42p to components involved in actin polarization, including Bni1p and Bud6p (Aip3p). Mol. Cell. Biol. *20*, 6244–6258.

8. Tong, Z., Gao, X.D., Howell, A.S., Bose, I., Lew, D.J., and Bi, E. (2007). Adjacent positioning of cellular structures enabled by a Cdc42 GTPase-activating protein-mediated zone of inhibition. J. Cell Biol. *179*, 1375–1384.

9. Tatebe, H., Nakano, K., Maximo, R., and Shiozaki, K. (2008). Pom1 DYRK regulates localization of the Rga4 GAP to ensure bipolar activation of Cdc42 in fission yeast. Curr. Biol. *18*, 322–330.

10. Kelly, F.D., and Nurse, P. (2011). Spatial control of Cdc42 activation determines cell width in fission yeast. Mol. Biol. Cell *22*, 3801–3811.

11. Wedlich-Soldner, R., Altschuler, S., Wu, L., and Li, R. (2003). Spontaneous cell polarization through actomyosin-based delivery of the Cdc42 GTPase. Science *299*, 1231–1235.

12. Marco, E., Wedlich-Soldner, R., Li, R., Altschuler, S.J., and Wu, L.F. (2007). Endocytosis optimizes the dynamic localization of membrane proteins that regulate cortical polarity. Cell *129*, 411–422.

13. Orlando, K., Sun, X., Zhang, J., Lu, T., Yokomizo, L., Wang, P., and Guo, W. (2011). Exo-endocytic trafficking and the septin-based diffusion barrier are required for the maintenance of Cdc42p polarization during budding yeast asymmetric growth. Mol. Biol. Cell *22*, 624–633.

14. Gachet, Y., Tournier, S., Millar, J.B., and Hyams, J.S. (2001). A MAP kinase-dependent actin checkpoint ensures proper spindle orientation in fission yeast. Nature *412*, 352–355.

15. Millar, J.B., Buck, V., and Wilkinson, M.G. (1995). Pyp1 and Pyp2 PTPases dephosphorylate an osmosensing MAP kinase controlling cell size at division in fission yeast. Genes Dev. *9*, 2117–2130.

16. Shiozaki, K., and Russell, P. (1995). Cell-cycle control linked to extracellular environment by MAP kinase pathway in fission yeast. Nature *378*, 739–743.

17. Kato, T., Jr., Okazaki, K., Murakami, H., Stettler, S., Fantes, P.A., and Okayama, H. (1996). Stress signal, mediated by a Hog1-like MAP kinase, controls sexual development in fission yeast. FEBS Lett. *378*, 207–212.

18. Brewster, J.L., and Gustin, M.C. (2014). Hog1: 20 years of discovery and impact. Sci. Signal. *7*, re7.

19. Saito, H., and Posas, F. (2012). Response to hyperosmotic stress. Genetics *192*, 289–318.

20. Cuadrado, A., and Nebreda, A.R. (2010). Mechanisms and functions of p38 MAPK signalling. Biochem. J. *429*, 403–417.

21. Zimmerman, S., Daga, R.R., and Chang, F. (2004). Intra-nuclear microtubules and a mitotic spindle orientation checkpoint. Nat. Cell Biol. *6*, 1245–1246.

22. Sawin, K.E., Lourenco, P.C., and Snaith, H.A. (2004). Microtubule nucleation at non-spindle pole body microtubule-organizing centers requires fission yeast centrosomin-related protein mod20p. Curr. Biol. *14*, 763–775.

23. Meadows, J.C., and Millar, J. (2008). Latrunculin A delays anaphase onset in fission yeast by disrupting an Ase1-independent pathway controlling mitotic spindle stability. Mol. Biol. Cell *19*, 3713–3723.

24. Riedl, J., Crevenna, A.H., Kessenbrock, K., Yu, J.H., Neukirchen, D., Bista, M., Bradke, F., Jenne, D., Holak, T.A., Werb, Z., et al. (2008). Lifeact: a versatile marker to visualize F-actin. Nat. Methods *5*, 605–607.

25. Huang, J., Huang, Y., Yu, H., Subramanian, D., Padmanabhan, A., Thadani, R., Tao, Y., Tang, X., Wedlich-Soldner, R., and Balasubramanian, M.K. (2012). Nonmedially assembled F-actin cables incorporate into the actomyosin ring in fission yeast. J. Cell Biol. *199*, 831–847.

26. Pérez, P., and Cansado, J. (2010). Cell integrity signaling and response to stress in fission yeast. Curr. Protein Pept. Sci. *11*, 680–692.

27. Smith, D.A., Morgan, B.A., and Quinn, J. (2010). Stress signalling to fungal stress-activated protein kinase pathways. FEMS Microbiol. Lett. *306*, 1–8.

28. Gaits, F., Degols, G., Shiozaki, K., and Russell, P. (1998). Phosphorylation and association with the transcription factor Atf1 regulate localization of Spc1/Sty1 stress-activated kinase in fission yeast. Genes Dev. *12*, 1464–1473.

29. He, B., and Guo, W. (2009). The exocyst complex in polarized exocytosis. Curr. Opin. Cell Biol. *21*, 537–542.

30. Goode, B.L., Eskin, J.A., and Wendland, B. (2015). Actin and endocytosis in budding yeast. Genetics *199*, 315–358.

31. Bishop, A.C., Ubersax, J.A., Petsch, D.T., Matheos, D.P., Gray, N.S., Blethrow, J., Shimizu, E., Tsien, J.Z., Schultz, P.G., Rose, M.D., et al. (2000). A chemical switch for inhibitor-sensitive alleles of any protein kinase. Nature *407*, 395–401.

32. Zuin, A., Carmona, M., Morales-Ivorra, I., Gabrielli, N., Vivancos, A.P., Ayté, J., and Hidalgo, E. (2010). Lifespan extension by calorie restriction relies on the Sty1 MAP kinase stress pathway. EMBO J. *29*, 981–991.

33. Wilkinson, M.G., Samuels, M., Takeda, T., Toone, W.M., Shieh, J.C., Toda, T., Millar, J.B., and Jones, N. (1996). The Atf1 transcription factor is a target for the Sty1 stress-activated MAP kinase pathway in fission yeast. Genes Dev. *10*, 2289–2301.

34. Shiozaki, K., and Russell, P. (1996). Conjugation, meiosis, and the osmotic stress response are regulated by Spc1 kinase through Atf1 transcription factor in fission yeast. Genes Dev. *10*, 2276–2288.

35. Kanoh, J., Watanabe, Y., Ohsugi, M., Iino, Y., and Yamamoto, M. (1996). *Schizosaccharomyces pombe* gad7^{+} encodes a phosphoprotein with a bZIP domain, which is required for proper G1 arrest and gene expression under nitrogen starvation. Genes Cells *1*, 391–408.

36. Petersen, J., and Hagan, I.M. (2005). Polo kinase links the stress pathway to cell cycle control and tip growth in fission yeast. Nature *435*, 507–512.

37. Grallert, A., Patel, A., Tallada, V.A., Chan, K.Y., Bagley, S., Krapp, A., Simanis, V., and Hagan, I.M. (2013). Centrosomal MPF triggers the mitotic and morphogenetic switches of fission yeast. Nat. Cell Biol. *15*, 88–95.

38. Shiozaki, K., Shiozaki, M., and Russell, P. (1998). Heat stress activates fission yeast Spc1/StyI MAPK by a MEKK-independent mechanism. Mol. Biol. Cell *9*, 1339–1349.

39. Lawrence, C.L., Maekawa, H., Worthington, J.L., Reiter, W., Wilkinson, C.R., and Jones, N. (2007). Regulation of *Schizosaccharomyces pombe* Atf1 protein levels by Sty1-mediated phosphorylation and heterodimerization with Pcr1. J. Biol. Chem. *282*, 5160–5170.

40. Sansó, M., Gogol, M., Ayté, J., Seidel, C., and Hidalgo, E. (2008). Transcription factors Pcr1 and Atf1 have distinct roles in stress- and Sty1-dependent gene regulation. Eukaryot. Cell *7*, 826–835.

41. Yamamoto, M., Imai, Y., and Watanabe, Y. (1997). Mating and sporulation in *Schizosaccharomyces pombe*. In The Molecular and Cellular Biology of the Yeast *Saccharomyces*, Volume 3: Cell Cycle and Cell Biology, J.R. Pringle, J.R. Broach, and E.W. Jones, eds. (Cold Spring Harbor Laboratory Press), pp. 1037–1106.

42. Harigaya, Y., and Yamamoto, M. (2007). Molecular mechanisms underlying the mitosis-meiosis decision. Chromosome Res. *15*, 523–537.

43. Su, S.S., Tanaka, Y., Samejima, I., Tanaka, K., and Yanagida, M. (1996). A nitrogen starvation-induced dormant G0 state in fission yeast: the establishment from uncommitted G1 state and its delay for return to proliferation. J. Cell Sci. *109*, 1347–1357.
44. Bendezú, F.O., and Martin, S.G. (2013). Cdc42 explores the cell periphery for mate selection in fission yeast. Curr. Biol. *23*, 42–47.
45. Sajiki, K., Hatanaka, M., Nakamura, T., Takeda, K., Shimanuki, M., Yoshida, T., Hanyu, Y., Hayashi, T., Nakaseko, Y., and Yanagida, M. (2009). Genetic control of cellular quiescence in *S. pombe*. J. Cell Sci. *122*, 1418–1429.
46. Yanagida, M., Ikai, N., Shimanuki, M., and Sajiki, K. (2011). Nutrient limitations alter cell division control and chromosome segregation through growth-related kinases and phosphatases. Philos. Trans. R. Soc. Lond. B Biol. Sci. *366*, 3508–3520.
47. Reiser, V., Raitt, D.C., and Saito, H. (2003). Yeast osmosensor Sln1 and plant cytokinin receptor Cre1 respond to changes in turgor pressure. J. Cell Biol. *161*, 1035–1040.
48. Harrison, J.C., Bardes, E.S., Ohya, Y., and Lew, D.J. (2001). A role for the Pkc1p/Mpk1p kinase cascade in the morphogenesis checkpoint. Nat. Cell Biol. *3*, 417–420.

Current Biology, Volume *26*

Supplemental Information

Remodeling of the Fission Yeast Cdc42 Cell-Polarity Module via the Sty1 p38 Stress-Activated Protein Kinase Pathway

Delyan R. Mutavchiev, Marcin Leda, and Kenneth E. Sawin

Figure S1

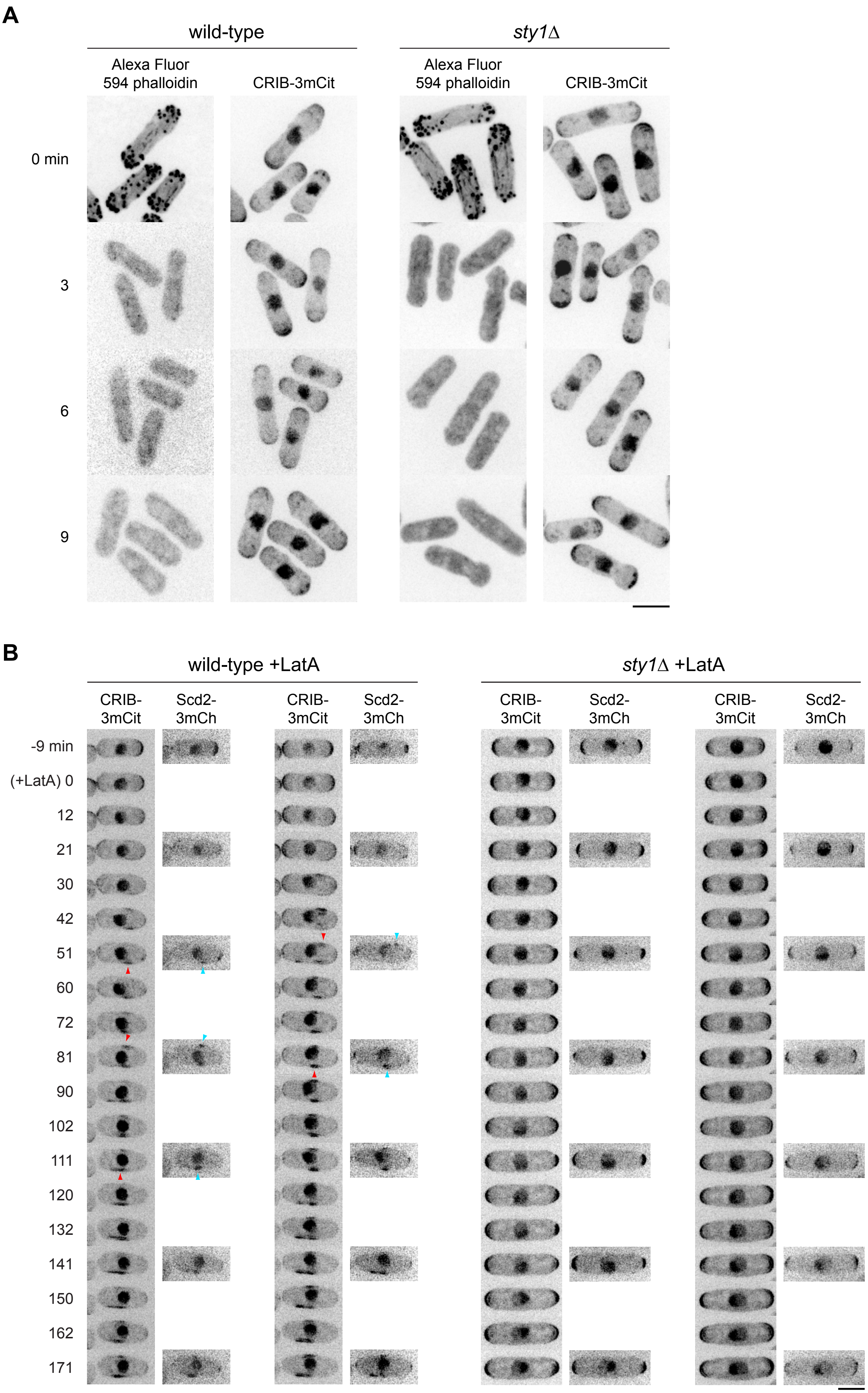
A
wild-type
sty1Δ
Alexa Fluor 594 phalloidin
CRIB-3mCit
Alexa Fluor 594 phalloidin
CRIB-3mCit
0 min
3
6
9
B
wild-type +LatA
sty1Δ +LatA
CRIB-3mCit
Scd2-3mCh
CRIB-3mCit
Scd2-3mCh
CRIB-3mCit
Scd2-3mCh
CRIB-3mCit
Scd2-3mCh
-9 min
(+LatA) 0
12
21
30
42
51
60
72
81
90
102
111
120
132
141
150
162
171

Figure S1 (related to Figures 1 and 2). LatA-induced actin depolymerization and dispersal of the Cdc42 polarity module.

A. Latrunculin A (LatA)-induced actin depolymerization in wild-type and *sty1*Δ cells, shown by Alexa Fluor 594 phalloidin staining of fixed cells, rather than by Lifeact-mCherry in live cells (which is shown in Figures 1 and 2). CRIB-3xmCitrine (CRIB-3mCit) fluorescence in the same cells is also shown. Times shown are relative to addition of LatA. These control experiments demonstrate that the presence/absence of Lifeact-mCherry does not alter the extent or rate of actin depolymerization by LatA. Because fixation and staining methods are optimized for actin preservation [S1], CRIB localization and cell morphology may be slightly altered relative to live cells.

B. Still images from movies of CRIB-3mCit and the Cdc42 polarity module scaffold protein Scd2-3xmCherry (Scd2-3mCh) in two wild-type cells and two *sty1*Δ cells after addition of 50 µM LatA. Times shown are relative to addition of LatA. Red and blue arrowheads indicate examples of ectopic patches of CRIB and Scd2, respectively, which co-localize. Together with previous work showing similar dispersal and patches for Scd2-3xGFP and for Cdc42 itself in response to LatA treatment [S2, S3], this co-localization demonstrates that CRIB dispersal and patches indicates re-localization of the Cdc42 polarity module at large rather than a specific disruption of the interaction between CRIB and the Cdc42 module. Because Scd2-3mCh is extremely faint and sensitive to photobleaching, it was imaged only at 30 min intervals, and Z-series in both channels (i.e. CRIB-3mCit and Scd2-3mCh) were acquired at 1 µm intervals.

Scale bars, 5 µm.

Figure S2

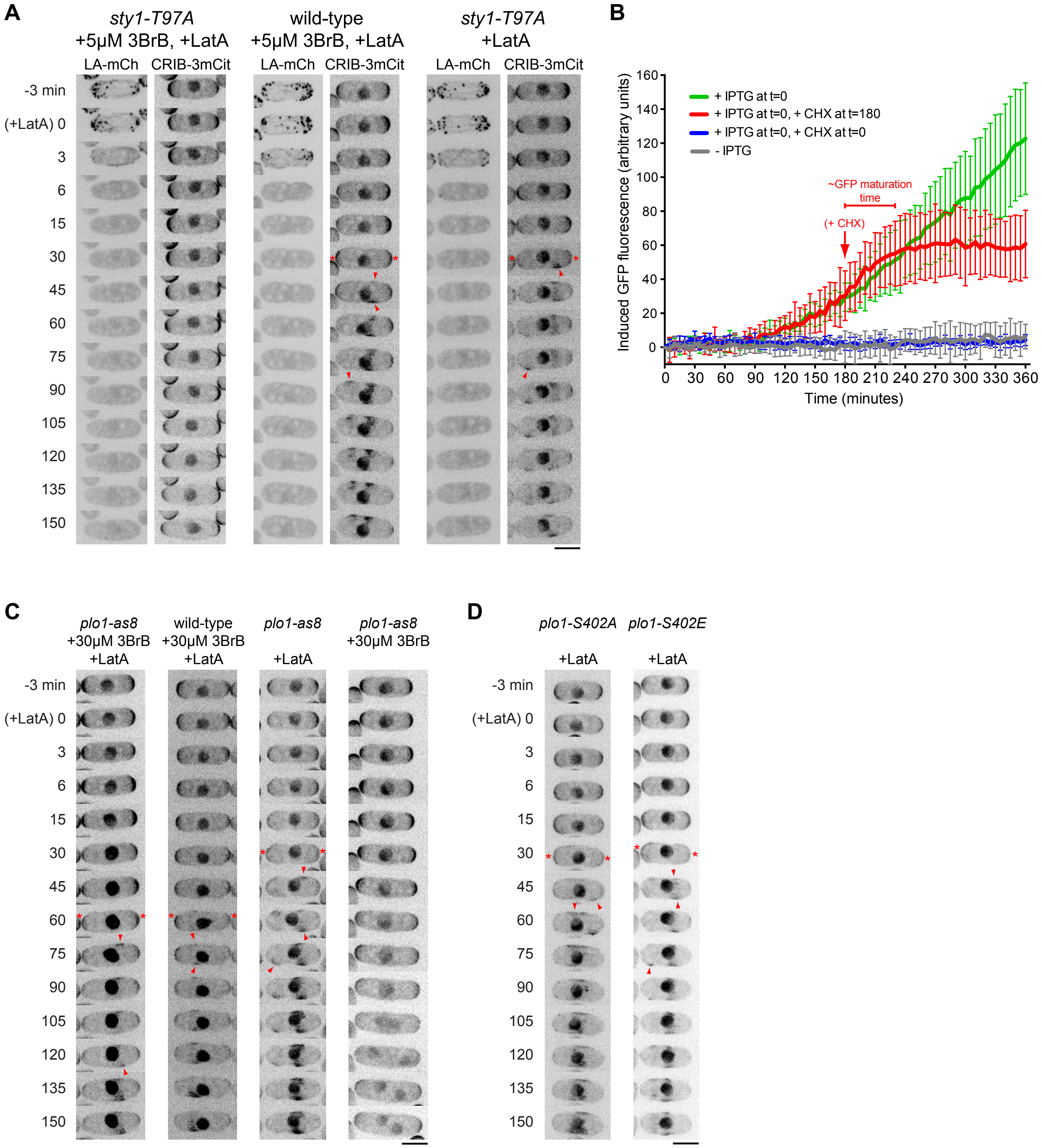
A
sty1-T97A +5μM 3BrB, +LatA
wild-type +5μM 3BrB, +LatA
sty1-T97A +LatA
LA-mCh
CRIB-3mCit
-3 min
(+LatA) 0
3
6
15
30
45
60
75
90
105
120
135
150
B
+ IPTG at t=0
+ IPTG at t=0, + CHX at t=180
+ IPTG at t=0, + CHX at t=0
- IPTG
~GFP maturation time
(+ CHX)
Induced GFP fluorescence (arbitrary units)
Time (minutes)
C
plo1-as8 +30μM 3BrB +LatA
wild-type +30μM 3BrB +LatA
plo1-as8 +LatA
plo1-as8 +30μM 3BrB
D
plo1-S402A +LatA
plo1-S402E +LatA

Figure S2 (related to Figure 2). Role of Sty1 in LatA-induced CRIB dispersal.

A. Control experiments showing that inhibition of LatA-induced CRIB dispersal by 3-BrB-PP1 in analog-sensitive *sty1-T97A* cells requires both the *sty1-T97A* allele and 3-BrB-PP1. Still images from movies of Lifeact-mCherry (LA-mCh) and CRIB-3xmCitrine (CRIB-3mCit) in: *sty1-T97A* cells and wild-type cells pre-treated with 5 μM 3-BrB-PP1 (3BrB) for 10 min, prior to addition of 50 μM LatA in the continued presence of 3BrB; and in *sty1-T97A* cells not pre-treated with 3BrB. Cell in left-most panel is reproduced from Figure 2C, with additional time-points. Asterisks indicate dispersal of CRIB from cell tips. Arrowheads indicate examples of ectopic CRIB patches after dispersal. Times shown are relative to addition of LatA.

B. Evidence that 100 μg/mL cycloheximide (CHX) completely inhibits protein synthesis in fission yeast. This provides additional controls for the experiments shown in Figure 2E. Wild-type cells containing an isopropyl β-D-1-thiogalactopyranoside (IPTG)-inducible *GFP* gene were assayed for GFP fluorescence under the conditions shown. Addition of IPTG leads to a large increase in fluorescence over time (green curve), but no increase occurs if CHX is added simultaneously with IPTG (blue curve). If CHX is added after the increase of fluorescence has begun (red curve), fluorescence continues to increase for about 45 min, which should correspond to the maturation time of already-synthesized GFP [S4]; however, subsequently there is no further increase. Error bars indicate SD.

C. Inhibition of polo kinase Plo1 does not affect LatA-induced CRIB dispersal. Still images from movies of CRIB-3xmCitrine in: analog-sensitive *plo1-as8* cells and wild-type cells pre-treated with 30 μM 3-BrB-PP1 (3BrB) for 10 min, prior to addition of 50 μM LatA in the continued presence of 3BrB; in *plo1-as8* cells not pre-treated with 3BrB prior to LatA addition; and in *plo1-as8* cells treated with 3BrB but not with LatA. Times shown are relative to addition of LatA. Asterisks indicate dispersal of CRIB from cell tips. Arrowheads indicate examples of ectopic CRIB patches after dispersal. In both *plo1-as8* and wild-type cells pre-treated with 30 μM 3BrB, CRIB dispersal and ectopic patch formation occur later than in non-pre-treated cells (see Figures 1A and S1B), and also later than in cells pre-treated with lower concentrations of 3BrB (see Figure S2A). This was reproducibly observed and may be due to weak off-target effects resulting from the high concentration of analog required to inhibit Plo1 [S5]. Right-most panel shows that addition of 30 μM 3BrB to *plo1-as8* cells prevents cytokinesis/septation, a characteristic feature of *plo1* loss-of-function [S6].

D. Mutation of a Sty1-dependent phosphorylation site (Ser 402) in Plo1 does not affect LatA-induced CRIB dispersal. Still images from movies of CRIB-3xmCitrine (CRIB-3mCit) in non-phosphorylatable *plo1-S402A* and phosphomimetic *plo1-S402E* cells [S7] after addition of 50 μM LatA. Times shown are relative to addition of LatA. Asterisks indicate dispersal of CRIB from cell tips. Arrowheads indicate examples of ectopic CRIB patches after dispersal. Note that in *plo1-S402A* and *plo1-S402E* cells, LatA-induced CRIB dispersal is indistinguishable from that observed in wild-type cells (see Figures 1A and S1B, and Movie S1), as well as that observed both in non-inhibited *plo1-as8* cells (Figure S2C) and in analog-inhibited *plo1-as8* cells (apart from the small 3BrB-dependent delay described above; Figure S2C). This is important because Ser 402 of Plo1 has been shown to be phosphorylated in a Sty1-dependent manner during recovery after heat stress (although this residue is not phosphorylated after oxidative or osmotic stress), and S402 phosphorylation has further been implicated to play a role in regulation of cell elongation after heat stress [S7]. However, our experiments shown here demonstrate that Plo1 Ser 402 does not play a role in CRIB dispersal after LatA treatment.

Scale bars, 5 μm.

Figure S3

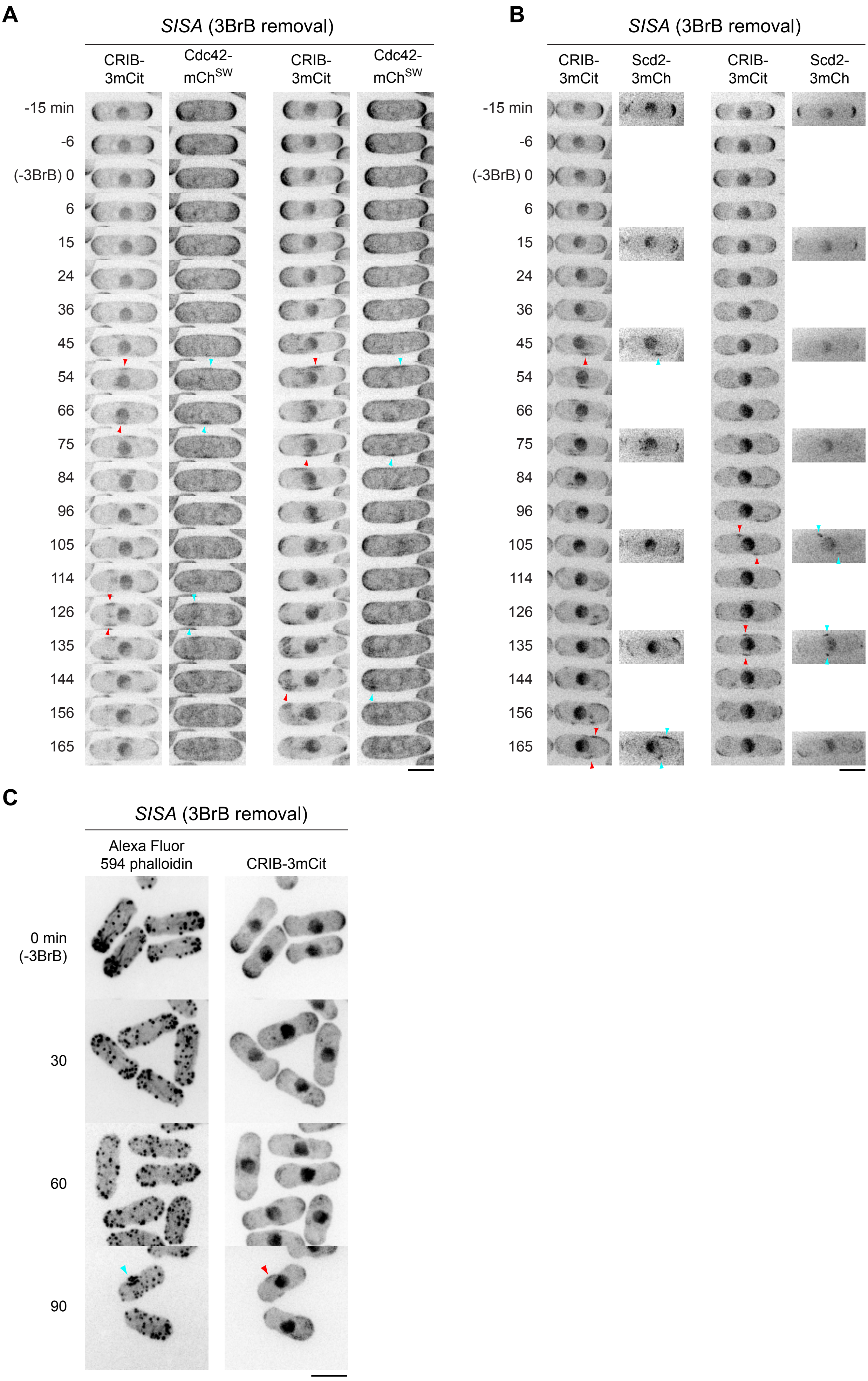

A
SISA (3BrB removal)
CRIB-3mCit
Cdc42-mCh[SW]
CRIB-3mCit
Cdc42-mCh[SW]
-15 min
-6
(-3BrB) 0
6
15
24
36
45
54
66
75
84
96
105
114
126
135
144
156
165
B
SISA (3BrB removal)
CRIB-3mCit
Scd2-3mCh
CRIB-3mCit
Scd2-3mCh
-15 min
-6
(-3BrB) 0
6
15
24
36
45
54
66
75
84
96
105
114
126
135
144
156
165
C
SISA (3BrB removal)
Alexa Fluor 594 phalloidin
CRIB-3mCit
0 min (-3BrB)
30
60
90

Figure S3 (related to Figure 3). Remodeling of cell polarity after Sty1 activation in *SISA* cells.

A. Still images from movies of CRIB-3mCit and internally ("sandwich")-tagged Cdc42-mCherry (Cdc42-mCh^{SW}; [S3]) in two *SISA* cells after Sty1 activation by removal of ATP-competitive analog 3-BrB-PP1 (3BrB). Times shown are relative to 3BrB removal. Red and blue arrowheads indicate examples of ectopic patches of CRIB and Cdc42, respectively, which co-localize. This co-localization demonstrates that CRIB dispersal and patches in Sty1-activated *SISA* cells indicates re-localization of the Cdc42 polarity module at large rather than a specific disruption of the interaction between CRIB and the Cdc42 module.

B. Still images from movies of CRIB-3mCit and the Cdc42 polarity module scaffold protein Scd2-3xmCherry (Scd2-3mCh) in two *SISA* cells after Sty1 activation by removal of 3BrB. Times shown are relative to 3BrB removal. Red and blue arrowheads indicate examples of ectopic patches of CRIB and Scd2, respectively, which co-localize. Together with results shown in Figure S3A, this co-localization demonstrates that CRIB dispersal and patches in Sty1-activated *SISA* cells indicates re-localization of the Cdc42 polarity module at large rather than a specific disruption of the interaction between CRIB and the Cdc42 module. Because Scd2-3mCh is extremely faint and sensitive to photobleaching, it was imaged only at 30 min intervals, and Z-series in both channels (i.e. CRIB-3mCit and Scd2-3mCh) were acquired at 1 μm intervals.

C. Depolarization of the actin cytoskeleton after Sty1 activation in *SISA* cells (triggered by removal of 3BrB), shown by Alexa Fluor 594 phalloidin staining of fixed cells, rather than by Lifeact-mCherry (LA-mCh) in live cells (which is shown in Figure 3). CRIB-3xmCitrine (CRIB-3mCit) fluorescence in the same cells is also shown. Times shown are relative to 3BrB removal. This control experiment demonstrates that actin depolarization in Sty1-activated *SISA* cells is not dependent on the presence of LA-mCh. Also note that clusters of cortical actin patches (blue arrowhead) are often associated with ectopic CRIB patches (red arrowhead). Because fixation and staining methods are optimized for actin preservation [S1], CRIB localization and cell morphology may be slightly altered relative to live cells.

Scale bars, 5 μm.

Supplemental Experimental Procedures

Yeast strain construction and cultures

Standard fission yeast techniques were used throughout for cell culture and genetic crosses [S8]. Cells were grown, as indicated, in: YE5S rich medium (using Difco yeast extract); EMM2 minimal medium; low-nitrogen (low-N) EMM2 minimal medium, which contains 0.5 g/L ammonium chloride instead of 5 g/L; EMM2-N, which is identical to EMM2 but lacks ammonium chloride altogether; or PMG minimal medium, which is identical to EMM2 but contains 4 g/L sodium glutamate instead of ammonium chloride as nitrogen source. Nutritional supplements were used at 175 mg/L. Solid media used 2% Difco Bacto agar. When working with *SISA* strains, to prevent activation of Sty1, 3BrB-PP1 (see below) was added to liquid media (5μM 3BrB-PP1 final concentration) and to solid media (5μM, or 30μM 3BrB-PP1 when waking strains, to prevent possible suppressor mutations). Mating for genetic crosses was performed on SPAS plates with nutritional supplements at 45 mg/L [S8]. Crosses were performed using tetrad dissection or random spore analysis, with confirmation by yeast colony PCR as appropriate. Tagging and deletion of genes were performed using PCR-based methods [S9]. Strains used in this study are listed below.

To generate the CRIB-3xmCitrine strain, CRIB and 3xmCitrine nucleic acid sequences were synthesized as two separate fragments by GeneArt (ThermoFisher). For the CRIB component, the sequence encoding the CRIB domain of the *S. cerevisiae* Gic2 protein (amino acids 1-208, with an intentional mutation W23A; [S10, S11]) was codon-optimized for *S. pombe* expression, and additional sequence was added to the 3' end to encode a C-terminal linker GDGAGLIN. The CRIB-plus-linker fragment was cloned into pMA-T (GeneArt) to generate the plasmid pKS1296. For the 3xmCitrine component, codon usage was varied in each of the three mCitrine sequences, so that each mCitrine nucleic acid sequence is only ~70% identical to the other two, although all three encode the same amino-acid sequence. This decreases the likelihood of undesired recombination occurring between tandem mCitrine sequences during genetic crosses. The 3xmCitrine fragment was cloned into pMK-RQ (GeneArt) to generate plasmid pKS1295. The 3xmCitrine fragment was excised from pKS1295 using SalI and BamHI and cloned into SalI and BamHI sites in pKS1296, placing the 3xmCitrine tag downstream of and in frame with the CRIB-plus-linker fragment, to generate plasmid pKS1305. Next, the entire CRIB-3xmCitrine fusion gene was excised from pKS1305 using NdeI and BamHI and cloned into NdeI and BamHI sites in the vector pRAD13 (kind gift from Y. Watanabe) which places the fusion gene under control of the *adh13* promoter (a weakened *adh1* promoter; [S12]), to generate plasmid pKS1374. pKS1374 was linearized using BlpI to allow for stable integration into the fission yeast *ars1* locus, using LEU2 as a selection marker. Cells expressing the *adh13*:CRIB-3xmCitrine fusion protein do not have any morphological defects. Like the previously published CRIB-3xGFP fusion protein [S13], the CRIB-3xmCitrine fusion protein shows some localization to the nucleus; this nuclear localization has been shown to be an artifact unrelated to Cdc42 [S3]. Use of a weaker promoter did not alter the CRIB-3xmCitrine nuclear localization.

To generate a strain with IPTG-inducible GFP expression, we used the plasmid pSK173 ([S14]; see important erratum, doi: 10.1002/yea.3136). This plasmid, also known as nmt::lacO*-GFP, contains a copy of GFP under the control of the thiamine-repressible *nmt1* promoter, with a lacO operator sequence inserted five base pairs downstream of the *nmt1* TATA box, as well as a copy of the lac repressor (lacI) fused to a nuclear localization sequence, under the control of an SV40 promoter. pSK173 was linearized using AfeI and stably integrated into the *ars1* genomic locus using *ura4+* as a selection marker.

Strains used in this study:

**Strain**	**Genotype**	**Source**
KS7305	*h- ars1(Blp*I*):*P*adh13:CRIB-3xmCitrine:LEU2 ade6-M216 leu1-32 ura4-D18*	This study
KS7311	*h- sty1Δ::kanMX ars1(Blp*I*):*P*adh13:CRIB-3xmCitrine:LEU2 ade6-M216 leu1-32 ura4-D18*	This study
KS7566	*h+ ars1(Blp*I*):*P*adh13:CRIB-3xmCitrine:LEU2* P*act1:lifeact-mCherry::leu1+ ade6-M216 leu1-32 ura4-D18*	This study
KS7660	*h+ sty1Δ::kanMX ars1(Blp*I*):*P*adh13:CRIB-3xmCitrine:LEU2* P*act1:lifeact-mCherry::leu1+ ade6-M210 leu1-32 ura4-D18*	This study
KS7737	*h- ars1(Blp*I*):*P*adh13:CRIB-3xmCitrine:LEU2 atf1Δ::kanMX6 ade6-M216 leu1-32 ura4-D18*	This study
KS7830	*h+ sty1-T97A ars1(Blp*I*):*P*adh13:CRIB-3xmCitrine:LEU2*	This study

	Pact1:lifeact-mCherry::leu1+ ade6-M216 leu1-32 ura4-D18	
KS8116	h- ars1(AfeI):nmt:lacO*:GFP:lacI-NLS:ura4+ ade6-M216 leu1-32 ura4-D18	This study
KS8140	h- sty1-mECitrine:KanMX6 ade6-M210 leu1-32 ura4-D18	This study
KS8164	h- ars1(BlpI):Padh13:CRIB-3xmCitrine:LEU2	This study
KS8217	h- ars1(BlpI):Padh13:CRIB-3xmCitrine:LEU2 plo1.as8:ura4+	This study
KS8226	h+ sty1-T97A wis1DD:12myc:ura4+ pyp1Δ::ura4+ pyp2Δ::LEU2 ars1(BlpI):Padh13:CRIB-3xmCitrine:LEU2 leu1-32 ura4-D18	This study
KS8276	h- wis1Δ::ura4+ ars1(BlpI):Padh13:CRIB-3xmCitrine:LEU2 ade6-M210 leu1-32 ura4-D18	This study
KS8311	h+ sty1-T97A wis1DD:12myc:ura4+ pyp1Δ::ura4+ pyp2Δ::LEU2 ars1(BlpI):Padh13:CRIB-3xmCitrine:LEU2 Pact1:lifeact-mCherry::leu1+ leu1-32 ura4-D18	This study
KS 8323	h- sty1-T97A ars1(BlpI):Padh13:CRIB-3xmCitrine:LEU2	This study
KS8332	h- sty1-T97A wis1DD:12myc:ura4+ pyp1Δ::ura4+ pyp2Δ::LEU2 ars1(BlpI):Padh13:CRIB-3xmCitrine:LEU2 cdc42-mCherry^{SW}:kanMX6 leu1-32 ura4-D18	This study
KS8764	h- Scd2-3xmCherry:kanMX6 ars1(BlpI):Padh13:CRIB-3xmCitrine:LEU2 ade6-M216 leu1-32 ura4-D18	This study
KS8765	h- Scd2-3xmCherry:kanMX6 sty1Δ::kanMX ars1(BlpI):Padh13:CRIB-3xmCitrine:LEU2 ade6-M216 leu1-32 ura4-D18	This study
KS8773	h+ sty1-T97A wis1DD:12myc:ura4+ pyp1Δ::ura4+ pyp2Δ::LEU2 ars1(BlpI):Padh13:CRIB-3xmCitrine:LEU2	This study
KS8779	h- plo1-S402A ars1(BlpI):Padh13:CRIB-3xmCitrine:LEU2	This study
KS8782	h+plo1-S402E ars1(BlpI):Padh13:CRIB-3xmCitrine:LEU2	This study

Base strains and strains from other laboratories:

**Strain**	**Genotype**	**Source**
KS1	h-	Lab stock
KS2	h+	Lab stock
KS515	h+ ade6-M210 leu1-32 ura4-D18	Lab stock
KS516	h- ade6-M216 leu1-32 ura4-D18	Lab stock
MBY6843 (KS6696)	h+ Pact1:lifeact-mCherry::leu1+ ade6-M216 leu1-32 ura4-D18	M. Balasubramanian
AV15 (KS7644)	h- atf1Δ::kanMX6	E. Hidalgo
AZ107 (KS7671)	h+ sty1-T97A ura4-D18	E. Hidalgo
KS2086 (KS7902)	h- wis1DD:12myc::ura4+ leu1-32 ura4-D18	K. Shiozaki
IH10368 (KS7918)	h- plo1.as8:ura4+ ura4-D18	I. Hagan
IH3758 (KS8735)	h- plo1.S402A	I. Hagan
IH3759 (KS8736)	h- plo1.S402E	I. Hagan
JM 281 (KS 7944)	h- pyp1Δ::ura4+ leu1-32 ura4-D18	J. Millar
JM 655 (KS 7945)	h+ pyp2Δ::LEU2 his7-366 leu1-32 ura4-D18	J. Millar
YSM2446 (KS8284)	h- cdc42-mCherry^{SW}:kanMX6	S. Martin

Physiological experiments, cell lysate preparation and western blotting

For all physiological experiments, cells were grown in a shaking water bath at 25°C. Latrunculin A (LatA) was obtained from either Alpha Laboratories (129-04361) or Abcam (ab144290) and dissolved in DMSO to make a 5 mM stock solution. 3-BrB-PP1 (4-Amino-1-tert-butyl-3-(3-bromobenzyl)pyrazolo[3,4-d]pyrimidine; A602985) was obtained from Toronto Research Chemicals and dissolved in methanol to make a 50 mM stock solution. Stock solutions were stored -80°C until use.

For growth curves of the *SISA* strain, cells were initially grown to a cell density of 2 x 10^6/mL in YE5S containing 5µM 3BrB-PP1. Cells were then filtered through a 0.45 µm membrane filter (Millipore, HVLP04700). Filters were then either transferred to one culture volume of YE5S containing 5µM 3BrB-PP1, or washed twice with YE5S (one culture volume per wash) and then transferred to one culture volume of YE5S. The resuspended cultures were returned to the water bath and samples were collected hourly. Each sample was fixed by addition of 37% formaldehyde (formalin) solution to a final concentration of 3.7% formaldehyde. Dilutions of the fixed samples were measured using Z2 Coulter Particle counter (Beckman-Coulter) using a 4-12 µm particle range setting.

To prepare cell lysates for measuring Sty1 activation on western blots after stress, cell cultures were grown in YE5S to a cell density of 1.25 x 10^7 cells/mL. Next, DMSO (1% final), LatA (50 µM final, with 1% DMSO final), or KCl (0.6 M final, from 3M stock in YE5S) was pipetted and mixed into the cell cultures. Samples of 1.6 mL (or 1.9 mL for KCl, because of increased volume) were collected into 2 mL screw-cap microcentrifuge tubes at the indicated times and immediately centrifuged at 4000 rpm at 4°C for 1 min. The supernatant was quickly removed and cell pellets placed on ice. Cell pellets were resuspended in 1 mL ice-cold 10mM $NaPO_4$ 0.5mM EDTA pH 7.5 buffer and centrifuged again at 13,000 rpm at 4°C for 1 min. The supernatant was removed, and the cell pellets were flash-frozen in liquid nitrogen and stored at -80°C until further processing. For cell lysis, 0.5 mm zirconium/silica beads pre-chilled to -20°C (BioSpec; 11079105z)) were added to the cell pellet, together with 40 µL ice-cold lysis buffer containing: 150 mM NaCl, 20 mM Tris-HCl pH 7.5, 0.05% Triton X-100, 10 µg/mL each of 'CLAAPE' protease inhibitors (chymostatin, leupeptin, antipain, pepstatin, E64), 2 mM DTT, 6 mM $MgCl_2$ 1 mM PMSF, 1 mM Benzamidine, 50 mM β-glycerophosphate, 50 mM EDTA, 1 mM NaF, 50 nM Calyculin A, 50 nM Okadaic acid, 100 nM Na_3VO_4, 2 mM AEBSF, 10% Glycerol. The suspension was put in a Ribolyser bead-beater (Hybaid) and four cycles of bead-beating were run: one cycle of 60 sec and three subsequent cycles of 30 sec each, all at speed setting "4". Between each cycle, samples were chilled in an ice-water slurry for 2 min. After bead-beating, the lysate in the screw-cap tubes was recovered by puncturing the tubes and centrifuging into a second tube. Cell lysates were then clarified by centrifugation at 13,000 rpm at 4°C for 10 min. One-third volume of 4X Laemmli sample buffer was then added to clarified lysates, which were then boiled at 100°C for 5 min and flash-frozen in liquid nitrogen.

To prepare *SISA* cell lysates for measuring Atf1 on western blots, parallel cell cultures were grown in YE5S containing 5µM 3BrB-PP1 to a cell density of 1.25 x 10^7 cells/mL. Cells were then filtered as described above, and washed twice, either with YE5S (one culture volume per wash) or with YE5S containing 5µM 3BrB-PP1 (one culture volume per wash), as appropriate. Cells were then resuspended in one culture volume of YE5S or YE5S containing 5µM 3BrB-PP1, as appropriate, and returned to the water bath. Samples of 1.9 mL were collected into 2 mL screw-cap microcentrifuge tubes at the indicated time points and processed as described above.

Cell lysates were separated by SDS-PAGE and transferred by western blotting to 0.2 µm nitrocellulose filters (Biorad). Western blots were first stained with Ponceau S and scanned to normalize for protein content between lanes. For measuring Sty1 activation, blots were probed with monoclonal rabbit anti-phospho-p38 primary antibody (Cell Signaling Technology; #4511) and IRDye 800CW Donkey anti-Rabbit secondary antibody (Licor; 925-32213). For measuring Atf1, blots were probed with monoclonal mouse anti-ATF1 primary antibody (Abcam; ab18123) and IRDye 800CW Donkey anti-Mouse secondary antibody (Licor; 925-32212). Blots were imaged using an Odyssey fluorescence imager (Licor) and quantified using Image Studio Lite (Licor). Ponceau S scans of blots were converted to 8-bit images using ImageJ and imported into Image Studio Lite for quantification.

Microscopy sample preparation and imaging

All imaging experiments were performed with exponentially growing cells grown at 25°C. Experiments were performed in YE5S medium, with the exception of: nitrogen-starvation (N-starvation) experiments, which were performed in low-N EMM2 and EMM2-N; and GFP IPTG-induction experiments, which were performed in PMG, with glucose added to the medium after autoclaving.

For preparation of imaging slides, coverslip dishes (MatTek; P35G-0.170-14-C.s) or 4-chamber glass bottom micro-slides (Ibidi; 80427) were placed on a 25°C heat block, coated with 1mg/mL soybean lectin (Sigma; L1395), left for 10 min, and washed with appropriate medium to remove excess lectin. Cell cultures were added to dishes/slides and left to settle for 15 minutes. Next, the cells were washed extensively with appropriate media using aspiration with at least 3 full exchanges of media (at least 1 mL each). Finally, 400µL (or 600 µL for N-starvation) of medium was used to cover the cells and the preparations were quickly placed in the microscope chamber at 25°C.

Live-cell fluorescence imaging was performed using a custom spinning-disk confocal microscope setup consisting of a Nikon TE2000 microscope base, attached to a modified Yokogawa CSU-10 unit (Visitech) and an iXon+ Du888 EMCCD camera (Andor). The microscope was also equipped with a 100x/1.45 NA Plan Apo objective (Nikon), Optospin IV filter wheel (Cairn Research), MS-2000 automated stage with CRISP autofocus (ASI), and temperature-controlled chamber maintained at 25°C (OKOlab). The microscope was controlled using Metamorph software (Molecular Devices).

All drug additions during imaging, except for N-starvation experiments, were performed by two consecutive medium exchanges with 400µl of drug-containing medium, using a 1mL polyethylene transfer pipette (Fisher Scientific, 1346-9118). All treatments were completed between the imaging intervals so that the time-lapse acquisition is not disrupted. Due to the mechanical force exerted during the medium exchanges, re-focusing of the sample between acquisitions was sometimes needed.

For conventional LatA addition experiments, YE5S containing 50 µM LatA and 1% DMSO (all final concentrations) was used. YE5S containing 1% DMSO was used as a negative control. Exchange of medium was performed between frames 4 and 5 of imaging (i.e. 9 -12 min after start of imaging).

For experiments involving 3-BrB-PP1 pre-treatment prior to LatA addition, exchange of medium to YE5S containing 5 µM (or 30 µM) 3-BrB-PP1 was performed 30 s before the start of imaging. Subsequent exchange of medium to YE5S containing 5 µM (or 30 µM) 3BrB-PP1 plus 50 µM LatA was performed between frames 4 and 5 of imaging, as above.

For cycloheximide (CHX) pre-treatments, exchange of medium to YE5S containing 100 µg/mL CHX (from 10 mg/mL CHX stock in water; Sigma; C1988) was performed 30 seconds before start of imaging. Subsequent exchange of medium to YE5S containing 100 µg/mL CHX plus 50 µM LatA was performed between frames 4 and 5 of imaging, as above.

For 3-BrB-PP1 removal experiments in *SISA* cells, cells were first grown and prepared for imaging in YE5S containing 5 µM 3-BrB-PP1. MatTek dishes were used for imaging. 3-BrB-PP1 removal was performed by three separate rounds of medium exchange after frames 3, 4 and 5 (i.e. 6 -15 min after start of imaging). Each round of medium exchange involved 6 x 1mL exchanges to YE5S. These additional exchanges (18 in total, far greater than in drug-addition experiments) were necessary for complete removal of 3-BrB-PP1. For this reason, the stated time of 3-BrB-PP1 removal in these experiments is approximate (we chose frame 5 to represent the “0 min” time). For the 3-BrB-PP1 re-addition experiments, a conventional medium exchange to YE5S containing 5 µM 3-BrB-PP1 was performed between frames 35 and 36 (102 -105 min after start of imaging).

For N-starvation experiments, cells were first grown as pre-cultures overnight in low-N EMM2 at 25°C, and then diluted into fresh low-N EMM2 and grown for another 12-16 hours at 25°C to a density of 1.5-5 x 10^6 cells/mL. To exchange to EMM2-N medium, 1 mL of cells was washed twice by 1 min centrifugation at 4,000 rpm and resuspension in an equal volume of EMM2-N medium. Cells were then placed on a lectin-coated MatTek coverslip dish as described above and covered with 600µL of EMM2-N. Prior to imaging, the cells were incubated on the dish for 10 hours at 25 °C, during which time most cells had divided twice to generate chains of four small cells. For addition of 3-BrB-PP1, the medium from the dish was withdrawn using a 1mL transfer pipette and quickly transferred to a microfuge tube already containing 0.6 µL of 5 mM 3-BrB-PP1 in EMM2-N, thereby converting the existing culture medium to EMM2-N containing 5µM 3-BrB-PP1. This medium was then returned to the cell dish, withdrawn once more and then returned again to the dish; this ensures good mixing without perturbing the cells. To mimic this treatment for control mock-treated cells (i.e. cells not treated with 3-BrB-PP1), medium was withdrawn from the dish, returned to the dish, withdrawn once more, and then returned again. The medium changes were performed between frames 11 and 12 (60-66 min after start of imaging). Medium changes of this nature were used instead of using fresh EMM2-N because fresh, “non-conditioned” EMM2-N appeared to cause a shock to the cells.

For IPTG-GFP induction experiments, cells were initially grown in PMG (with appropriate supplements), and exchange of medium was performed as described above for drug addition. PMG was exchanged either to: fresh PMG; or to fresh PMG containing 5 mM IPTG (from 1M stock in water); or to fresh PMG containing 5 mM IPTG plus 100 µg/mL CHX. Exchange of medium was performed 30 s before the start of imaging. In an additional experiment, to assay inhibition of protein synthesis after GFP expression was already induced, medium was exchanged from PMG to PMG containing 5 mM IPTG at 30 s before the start of imaging, and then later from PMG containing 5 mM IPTG to PMG containing 5mM IPTG plus 100 µg/mL CHX, between frames 36 - 37 (175 -180 min after start of imaging).

For DIC images of *SISA* cells after 3-BrB-PP1 removal, 1mL of exponentially growing cells in YE5S containing 5µM 3-BrB-PP1 was washed twice by 1 min centrifugation at 4,000 rpm and resuspension in an equal volume of YE5S. Washed cells were then further diluted with an additional 4 mL of YE5S and incubated at 25° for 24 hours, prior to imaging. Images were taken using a

DeltaVision Elite system using Olympus 60x / 1.42 Oil Plan APO objective. Control cells grown in YE5S containing 5μM 3-BrB-PP1 were imaged during exponential growth.

For phalloidin staining of fixed cells after LatA treatment, cells *not* expressing Lifeact-mCherry were grown to exponential phase in YE5S, and LatA was added to a final concentration of 50 μM LatA. Fixation with formaldehyde and staining with Alexa Fluor 594 phalloidin (Molecular Probes) were exactly as described previously [S1]. The zero time point represents cells just prior to LatA addition.

For phalloidin staining of Sty1-activated *SISA* cells, *SISA* cells (*not* expressing Lifeact-mCherry) were grown in YE5S containing 5μM 3-BrB-PP1, and then washed twice by filtration and resuspension in YE5S medium lacking 5μM 3-BrB-PP1, as described above for physiological experiments. Fixation and staining procedures were exactly as described previously [S1]. The zero time point represents cells just prior to filtration.

Image acquisition parameters:

**Image Type**	**Laser power (%)*, exposure time (ms)**			**Z-series**	**Time-lapse**
	**488 nm**	**514 nm**	**594 nm**		
CRIB-3xmCitrine		2%, 40 ms		11x 0.6 μm	60 x 3 min
Lifeact-mCherry			30%, 100 ms	11x 0.6 μm	60 x 3 min
CRIB-3xmCitrine (N-starvation)		2%, 100 ms		11x 0.6 μm	40 x 6 min
GFP-IPTG	5%, 150 ms			11x 0.9 μm	72 x 5 min
Sty1-mECitrine		2%, 200 ms		9x 0.7 μm	30 x 5 min
Cdc42-mCherry^{SW}			30%, 100ms	11x 0.6 μm	60 x 3 min
CRIB-3xmCitrine (with Scd2-3xmCherry)		2%, 40 ms		5x 1 μm	60 x 3 min
Scd2-3xmCherry			80%, 300ms	5x 1 μm	6 x 30 min
Alexa Fluor 594 phalloidin (fixed cells)			60%, 200ms	11x 0.6 μm	N/A
CRIB-3xmCitrine (fixed cells)		10%, 100ms		11x 0.6 μm	N/A

* "100%" power = 50 mW for 488 nm laser, 40 mW for 514 nm laser, and 50mW for 594 nm laser.

Processing of the acquired raw images was done using ImageJ (NIH), using the StackReg plugin for Rigid Body registration (http://bigwww.epfl.ch/publications/thevenaz9801.html) and KymoResliceWide plugin for kymographs (http://fiji.sc/KymoResliceWide). Images were adjusted using linear contrast enhancement and are presented as maximum or sum projections containing the entire cell volume. In one image sequence (corresponding to the wild-type cells shown in Figure 4B, and the same cells in Movie S4), a small manual linear contrast adjustment was necessary for three frames of the image sequence, due to a brief change in ambient light conditions; this did not affect the outcome of the experiment or its interpretation. Formatting of images for publication was done using Illustrator CS3 (Adobe). Movies were assembled using ImageJ and QuickTime (Apple).

Quantification of Sty1-mECitrine levels in the nucleus was obtained from movies by automated image analysis. For DMSO treatment, 14 cells were analyzed. For LatA treatment, 27 cells were analyzed. First, segmentation algorithms were used to identify cells and nuclei from maximum projections of z-stacks. Cell segmentation was carried out by the active contour Chan-Vese method, implemented in MATLAB (R2013a; Mathworks). Initial binary masks for cell segmentation were obtained using multi-threshold Otsu's method (*multithresh* in MATLAB) with nine thresholds. Bitmaps containing only those pixels that were above the fifth threshold were then processed using additional morphological operations to: 1) first, identify pixel clusters ("connected components"; *bwconncomp* in MATLAB); 2) then, join nearby clusters by removing holes between neighboring clusters; and 3) finally, remove small pixel clusters (= out of focus cells) and pixel clusters touching frame borders (= partial, incomplete cells). Nucleus segmentation was carried out starting with the masks obtained by cell segmentation. First, pixel intensity values in each individual cell were separately normalized to an interval from 0 to 1. Then, multi-threshold Otsu's method with five thresholds was used to construct bitmaps containing only those pixels that were above the third threshold. Identification of morphologically connected clusters was then used to generate a mask for the nucleus belonging to a given cell. Cells that contained two nuclei (i.e. dividing cells) were rejected from analysis. From the cell segmentation and nucleus segmentation we obtained binary masks of nucleus and corresponding cytosol (by subtracting nucleus mask from cell mask) for every cell, as well as a binary mask for extracellular space (background). Sum projections of z-stacks were used for

quantification of signals inside these masks. The nucleus-specific fluorescence intensity was calculated as $\left[\frac{(I_n - I_b)}{(I_c - I_b)} - 1\right]$, where I_n, I_b and I_c are average intensities in nucleus, cytosol and background, respectively. We note that in these experiments, a small, transient increase in Sty1-mECitrine nuclear fluorescence was observed in DMSO-treated control cells upon addition of DMSO. This is reproducible and appears to be a consequence of acute DMSO addition, from which the cell rapidly recovers.

For quantification of GFP expression under different conditions in IPTG-induction experiments, movies were acquired at multiple stage positions for each condition. For each condition, the time-course of expression was then determined as follows: First, sum projections of images were made for all time-points of movies, and background extracellular fluorescence was subtracted to calculate the value of the fluorescent signal $S_{c,t}$ for a given cell c (or small group of cells) at a given time-point t. These values were then normalized to the fluorescent signal $S_{c,1}$ for the same cell (or small group of cells) at the first time-point. Then, for each time-point t, the mean value of normalized fluorescence was calculated by averaging over all n cells imaged. We then subtracted 1 from mean values in order to generate a baseline of zero at the first time-point, and we then multiplied this result by an arbitrary constant (100) to generate a final value $100\times\left[\frac{1}{n}\left(\sum_{c=1}^{n}\frac{S_{c,t}}{S_{c,1}}\right) - 1\right]$, which represents induced GFP fluorescence (in arbitrary units) for each time-point t. For each of the different conditions, the number of cells (n) imaged was as follows: -IPTG (n =26); +IPTG at t=0, +CHX at t=0 (n =23); +IPTG at t=0, +CHX at t=180 min (n =19); +IPTG (n =16).

Supplemental References

S1. Sawin, K.E., and Nurse, P. (1998). Regulation of cell polarity by microtubules in fission yeast. J Cell Biol *142*, 457-471.

S2. Kelly, F.D., and Nurse, P. (2011). Spatial control of Cdc42 activation determines cell width in fission yeast. Mol Biol Cell *22*, 3801-3811.

S3. Bendezu, F.O., Vincenzetti, V., Vavylonis, D., Wyss, R., Vogel, H., and Martin, S.G. (2015). Spontaneous Cdc42 polarization independent of GDI-mediated extraction and actin-based trafficking. PLoS Biol *13*, e1002097.

S4. Gordon, A., Colman-Lerner, A., Chin, T.E., Benjamin, K.R., Yu, R.C., and Brent, R. (2007). Single-cell quantification of molecules and rates using open-source microscope-based cytometry. Nat Methods *4*, 175-181.

S5. Grallert, A., Patel, A., Tallada, V.A., Chan, K.Y., Bagley, S., Krapp, A., Simanis, V., and Hagan, I.M. (2013). Centrosomal MPF triggers the mitotic and morphogenetic switches of fission yeast. Nat Cell Biol *15*, 88-95.

S6. Ohkura, H., Hagan, I.M., and Glover, D.M. (1995). The conserved Schizosaccharomyces pombe kinase plo1, required to form a bipolar spindle, the actin ring, and septum, can drive septum formation in G1 and G2 cells. Genes Dev *9*, 1059-1073.

S7. Petersen, J., and Hagan, I.M. (2005). Polo kinase links the stress pathway to cell cycle control and tip growth in fission yeast. Nature *435*, 507-512.

S8. Forsburg, S.L., and Rhind, N. (2006). Basic methods for fission yeast. Yeast *23*, 173-183.

S9. Bahler, J., Wu, J.Q., Longtine, M.S., Shah, N.G., McKenzie, A., 3rd, Steever, A.B., Wach, A., Philippsen, P., and Pringle, J.R. (1998). Heterologous modules for efficient and versatile PCR-based gene targeting in Schizosaccharomyces pombe. Yeast *14*, 943-951.

S10. Jaquenoud, M., and Peter, M. (2000). Gic2p may link activated Cdc42p to components involved in actin polarization, including Bni1p and Bud6p (Aip3p). Mol Cell Biol *20*, 6244-6258.

S11. Okada, S., Leda, M., Hanna, J., Savage, N.S., Bi, E., and Goryachev, A.B. (2013). Daughter cell identity emerges from the interplay of Cdc42, septins, and exocytosis. Dev Cell *26*, 148-161.

S12. Sakuno, T., Tada, K., and Watanabe, Y. (2009). Kinetochore geometry defined by cohesion within the centromere. Nature *458*, 852-858.

S13. Tatebe, H., Nakano, K., Maximo, R., and Shiozaki, K. (2008). Pom1 DYRK regulates localization of the Rga4 GAP to ensure bipolar activation of Cdc42 in fission yeast. Curr Biol *18*, 322-330.

S14. Kjaerulff, S., and Nielsen, O. (2015). An IPTG-inducible derivative of the fission yeast nmt promoter. Yeast *32*, 469-478.
